# Individual-level changes in religious/spiritual beliefs and behaviors over three decades in the parental generation of the ALSPAC cohort, UK

**DOI:** 10.1080/2153599X.2022.2156584

**Published:** 2023-01-06

**Authors:** Daniel Major-Smith, Isaac Halstead, Jimmy Morgan, Hamid Reza Tohidinik, Yasmin Iles-Caven, Jean Golding, Kate Northstone

**Affiliations:** a Centre for Academic Child Health, Population Health Sciences, Bristol Medical School, University of Bristol, Bristol, UK; b MRC Integrative Epidemiology Unit, University of Bristol, Bristol, UK; c Population Health Sciences, Bristol Medical School, University of Bristol, Bristol, UK

**Keywords:** ALSPAC, religion, longitudinal, cohort study, descriptive

## Abstract

Longitudinal data on religious/spiritual beliefs and behaviors (RSBB) are essential for understanding both how religion shapes our lives and the factors determining religiosity. Despite this importance, there are few longitudinal studies with detailed and repeated RSBB data. Using data spanning nearly 30 years from the parental generation of the Avon Longitudinal Study of Parents and Children (ALSPAC) based in the Southwest of England, we describe individual-level changes in various aspects of self-reported RSBB (religious belief, affiliation, and attendance, among others) measured on four occasions (pregnancy, plus 5, 9, and 28 years post-partum; approx. 3600 mothers and 1200 partners have data at all four time-points). Although RSBBs were generally consistent over time, a shift towards non-religiosity was observed; exceptions included Roman Catholic affiliation, which was remarkably stable over three decades, and religious attendance, which increased from pregnancy to 5 years, before declining at 28 years. Most changes in RSBB were minor, e.g., between “yes” and “not sure” regarding religious belief, rather than between “yes” and “no.” We also provide a simple illustrative example of how these longitudinal data can be analyzed. In addition to describing these longitudinal patterns, this paper will help inform future research using ALSPAC’s longitudinal RSBB data.

## Introduction

In recent years there has been increasing recognition that religion is an important suite of beliefs and behaviors which shapes our lives and may impact health and well-being. This has resulted in an increase in the scientific study of religious/spiritual beliefs and behaviors (RSBB), both as an exposure (e.g., the impact of religion on health) and as an outcome (e.g., factors causing religiosity (Gervais et al., 2021; Koenig et al., 2012; VanderWeele, 2017)). Despite this trend, and the potential importance of RSBB for health outcomes, RSBB factors are regularly overlooked in main-stream health research, a key barrier of which is the lack of high-quality prospective studies with RSBB data (Shields & Balboni, 2020). Prospective studies with repeated RSBB measures can also help assess causality, which may be more difficult from cross-sectional data collection (Hernán & Robins, 2020; VanderWeele et al., 2016). However, there are few longitudinal population-based studies with repeated RSBB data; consequently, much of the existing research on health and religion involves small sample sizes, cross-sectional designs, and retrospective data collection (Koenig et al., 2012).

The Avon Longitudinal Study of Parents and Children (ALSPAC) based in the Southwest of England is an exception, as it has RSBB data measured repeatedly in the parental generation since the study’s inception over 30 years ago. While it has previously been shown that overall religiosity has declined over this period (Iles-Caven et al., 2019; Iles-Caven, Bickerstaffe, et al., 2021; Iles-Caven, Gregory, et al., 2021), the publications to date have only described broad population-level statistics, and have not explored how RSBB varied at an individual level. This individual-level focus is necessary to understand how RSBB changes over the life-span (e.g., stability, increases or decreases in belief), and the factors associated with these changes (Ingersoll-Dayton et al., 2002; McCullough et al., 2005).

In addition to providing a platform for future work on religion and health, describing these patterns can also help inform various debates in the scientific study of religion, such as how changes in religiosity over the life-span—if any—contribute to growing societal secularization (Bruce, 2011; Norris & Inglehart, 2011). For instance, using 10 years of longitudinal data from the British House-hold Panel Survey (from 1991 to 2001), and other cross-sectional sources, it has been suggested that RSBB is relatively stable over adulthood, and that increasing secularization is predominantly due to the intergenerational transmission of RSBB (Bruce, 2011; Bullivant, 2019; Crockett & Voas, 2006). Such long-term longitudinal studies with repeated individual-level data are rare, however; studies such as ALSPAC, which has almost 30 years of longitudinal RSBB data, are a valuable resource to explore these, and many other, questions.

The aim of this paper is to describe changes in RSBB among ALSPAC parents at an individual-level, exploring if, and how, religiosity varies over time. We examine a range of RSBB measures, including belief in God/a divine power, religious affiliation, and attendance at a place of worship, among others. We will also present a simple descriptive analysis exploring whether sociodemographic factors are associated with changes in religious belief to highlight how these data can be analyzed (as this analysis is purely illustrative, it is intentionally atheoretical; we leave it to future studies using these data to answer more theoretically-informed questions). In addition to describing these data and assessing the stability or otherwise of religious beliefs and behaviors, this paper will inform future research in this area using ALSPAC’s repeated RSBB data. For instance, many longitudinal modeling approaches—such as structured life-course models (Smith et al., 2015, 2016) or modeling latent longitudinal trajectories (Herle et al., 2020)—require variation over time in order to be applied effectively; if RSBB does not vary over time in this cohort, or varies only slightly, then these methods may not be appropriate. Here we will focus on repeated RSBB data from the parental ALSPAC generation, spanning approximately 30 years.

## Methods

An analysis plan for all analyses reported in this paper was pre-registered on the Open Science Foundation (OSF) website (https://osf.io/w9t2y/), and any deviations from the published protocol are noted in section S1 of the supplementary information.

### Participants

Pregnant women resident in Bristol (UK) and surrounding areas with expected dates of delivery between 1st April 1991 and 31st December 1992 were invited to take part in the study. The initial number of pregnancies enrolled was 14,541, comprising a total of 14,676 fetuses, resulting in 14,062 live births and 13,988 children alive at 1 year of age (Boyd et al., 2013; Fraser et al., 2013). The current research focuses specifically on the parents of the study child (also known as ALSPAC Generation-0, or G0). For this study, one pregnancy was removed if the mother had two pregnancies enrolled in ALSPAC (to avoid repeated data from the same parent), and observations for participants who had withdrawn consent for their data to be used were also excluded.

For each mother, we also included their associated partner, usually the father of the study child. Partners/fathers (hereafter “partners”) were not formally enrolled into ALSPAC but were given partner-based questionnaires by the mother (if she had a partner and chose to invite them). This means that partner-based questionnaires may not have been completed by the same partner over time (although numbers of such cases are relatively small); for the purposes of this study, we assume that the identity of the partner is the same over all waves of data collection used. A total of 14,157 mothers and 14,157 associated partners were included in the final dataset, although only 11,607 of these partners have been in contact with the study since its inception (for more information on these partners, please see the upcoming ALSPAC partners cohort profile paper; Northstone et al., in prep). Please note that the study website contains details of all the data that is available through a fully searchable data dictionary and variable search tool: http://www.bristol.ac.uk/alspac/researchers/our-data/.

### Data

The RSBB variables were assessed during pregnancy (mean mother’s age at birth = 28.0 years [SD = 5.0; range = 16–43]; mean partner’s age in pregnancy = 30.4 years [SD = 5.8; range = 15–70]), and at 5, 6, 9, and 28 years post-partum (Table 1; for a discussion regarding coding decisions for questions which changed over time, as indicated in the footnotes to Table 1, see section S2 of the supplementary information). Note that for brevity, from now on we will refer to these “years post-partum” time-points as simply the number of years since delivery (e.g., “28 years,” rather than “28 years post-partum”). Study data for the 28 years questionnaire were collected and managed using RED-Cap electronic data capture tools hosted at the University of Bristol (Harris et al., 2009).

In addition to these variables, we also used RSBB categories derived by latent class analysis at each time-point (i.e., using RSBB data available at each questionnaire to construct latent classes of religiosity). The latent classes were labeled as: “highly religious” (characterized by believing in God/a divine power, attending a place of worship regularly and obtaining help/support from members of their own or other religious groups), “moderately religious” (believing in God/a divine power, but less likely to attend a place of worship or obtain help/support from members of their own or other religious groups), “agnostics” (not sure if believe in God/a divine power and do not attend a place of worship), and “atheists” (do not believe in God/a divine power or attend a place of worship). The latent classes derived at each time-point are broadly consistent with one another (Table S1; for more details on how these latent classes were constructed, see (Halstead et al., 2022)).

For the simple illustrative analysis of how this longitudinal data could be analyzed, we used the mother’s “belief in God/a divine power” during pregnancy and at 9 years as an example. Given the multitude of possibilities of exploring patterns of change between these two time-points, we considered two methods for coding these variables (Table 2). In the first, we coded mothers into four groups: (i) consistent believers (answered “yes” at both time-points); (ii) consistent non-believers (answered “no” or “not sure” at both time-points); (iii) new believers (answered “no” or “not sure” in pregnancy, but “yes” at 9 years); and (iv) new non-believers (answered “yes” in pregnancy, but “no” or “not sure” at 9 years). In the second method, we coded mothers into five groups: (i) no change (same response at both time-points); (ii) slight increase in RSBB (“not sure” in pregnancy and “yes” at 9 years, or “no” in pregnancy and “not sure” at 9 years); (iii) major increase in RSBB (from “no” in pregnancy to “yes” at 9 years); (iv) slight decrease in RSBB (“yes” in pregnancy to “not sure” at 9 years, or “not sure” in pregnancy to “no” at 9 years); and (v) major decrease in RSBB (from “yes” in pregnancy to “no” at 9 years). The first method distinguishes between consistent believers and non-believers (which are grouped together in the second method), while the second method distinguishes between the size of the transition (small vs large; which are grouped together in the first method).

The following maternal exposures were used to assess whether they were associated with these trajectories of religious belief: age at birth of the study child (in years), ethnicity (White vs other than White), socioeconomic position—as proxied by highest maternal education (measured on a 5-point scale from no/low qualifications to university degree), household income (log GBP income per week), home ownership status (owned/mortgaged vs rented vs council/housing association vs other) and index of multiple deprivation (quintiles)—and whether this was their first pregnancy (yes vs no). All exposures were measured in pregnancy, other than household income which was measured when the study children were approximately three/four years old.

### Analysis

For each of the RSBB variables above (Table 1), in addition to the latent RSBB classes, we describe the individual-level changes in RSBB and visualize these transitions through a Sankey (or alluvial) plot; this graph begins with the first time-point (e.g., during pregnancy), then for each category shows how these participants answered at the next time-point (e.g., 5 years post-partum), and so on forward through time. This allows us to track changes in RSBB over time, explore the stability of RSBB, and identify whether certain transitions are more common than others (e.g., given the overall decline in religiosity over time in the ALSPAC parents, losses of belief are likely to be more common than gains in belief). Given that the 5- and 6-year data are so close together in time, we excluded the 6-year data for this paper because any changes in RSBB over a single year are likely to be minor, and because data from pregnancy, 5 and 9 years are approximately equally spaced apart, making the transitions more comparable. We therefore focused just on pregnancy, 5, 9, and 28 years data (with the exception of the following questions, which were only asked 6, 9, and 28 years: “Do you ‘pray’ even if not in trouble?” and “Are you bringing your child up in this faith?”). This approach was repeated for mothers and partners.

One complication with this analysis is that there must be no missing data for each of the time-points. As ALSPAC participation rates have declined over time (Boyd et al., 2013; Cornish et al., 2020; Fraser et al., 2013), and not every participant completed every questionnaire, the amount of missing data is considerable; for instance, only ~3600 mothers and ~1200 partners have fully-observed pregnancy, 5, 9, and 28 years RSBB data. In addition to reducing the sample size, this attrition may result in bias if RSBBs—or factors associated with RSBB—are related to continued participation in ALSPAC (Morgan et al., 2022). To partially address this issue, we explored both RSBB data from pregnancy through to 28 years, in addition to from pregnancy to just 9 years; as sample sizes are larger in the 9 years data (7983 mothers completed the 9 years questionnaire, while 4819 completed the 28 years questionnaire), the risk of bias is likely to be smaller. However, we note that this is unlikely to fully remove all potential bias due to selection.

In addition to describing this longitudinal RSBB data, we also present a simple example of how this data could be analyzed in future work (as described above; Table 2). As the outcome—”change in RSBB”—is either a four- or five-level categorical variable, we performed a series of multinomial regressions with each of the exposure variables described above (age, ethnicity, socioeconomic position and first-time pregnancy). Other than the age-only model, all models adjusted for maternal age to remove this common source of confounding. These are intended to be simple illustrative examples of how these data can be analyzed, the results of which should not be taken as causal estimates. All analyses were conducted in R version 4.0.4 (R Development Core Team, 2021). Sankey plots were constructed using the R package “ggalluvial” (Brunson & Read, 2020).

## Results

### Mothers’ changes in RSBB over time

**Figure 1:**
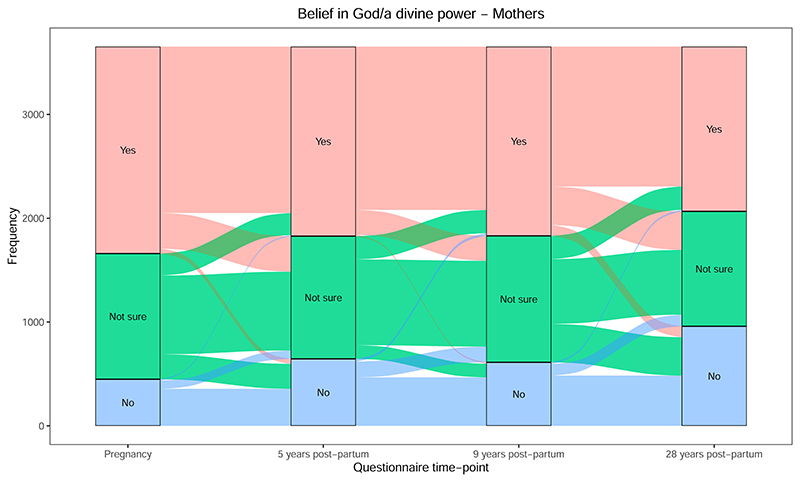
Change in belief in God/a divine power from pregnancy to 28 years post-partum for mothers (*n* = 3653). These and all subsequent Sankey plots begin with the first time-point (here, pregnancy), then for each variable category show how these participants answered at the next time-point (e.g., 5 years post-partum), and so on forward through time. For instance, in the figure here the majority (80%) of participants who answered “yes” in pregnancy also answered “yes” 5 years post-partum (the line between “yes” in pregnancy and “yes” 5 years post-partum), just under 20% changed from “yes” in pregnancy to “not sure” 5 years post-partum (the line between “yes” in pregnancy and “not sure” 5 years post-partum), and a small number (2%) changed from “yes” in pregnancy to “no” 5 years post-partum (the line between “yes” in pregnancy and “no” 5 years post-partum).

Descriptive statistics for all RSBB variables for mothers at each time-point are displayed in Table S2; results are displayed for the whole sample, for mothers with complete pregnancy to 28 year data, and for mothers with complete pregnancy to 9-year data. The change in mothers’ “belief in God/a divine power” over time, from pregnancy to 28 years, is displayed in Figure 1 (for pregnancy to 9 years, see Figure S1). The proportion of non-believers increases—and the proportion of believers decreases—from pregnancy to 5 years, remains stable to 9 years, and then decreases at 28 years. Between each time-point, there is a high degree of cross-over between neighboring beliefs (i.e., between “yes” and “not sure,” or “no” and “not sure”), but relatively few large-scale changes in belief (i.e., between “yes” and “no”). Overall, transitions appear to be toward a reduction in belief (i.e., from “yes” to “not sure,” or “not sure” to “no”). In contrast, “yes” responses to “ever been helped by God/a divine power” remained relatively stable from pregnancy to 28 years, with the increase in “no” responses predominantly coming from the “not sure” category (Figure S2; Figure S3 for pregnancy to 9 years). A similar pattern emerges for the question “would appeal to God if in trouble” (Figure S4; Figure S5 for pregnancy to 9 years). “Would pray, even if not in trouble” was only asked from 6 years onwards, and was similar at 6 and 9 years, but from 9 to 28 years the proportion of mothers who answered “no” increased (Figure S6).

**Figure 2:**
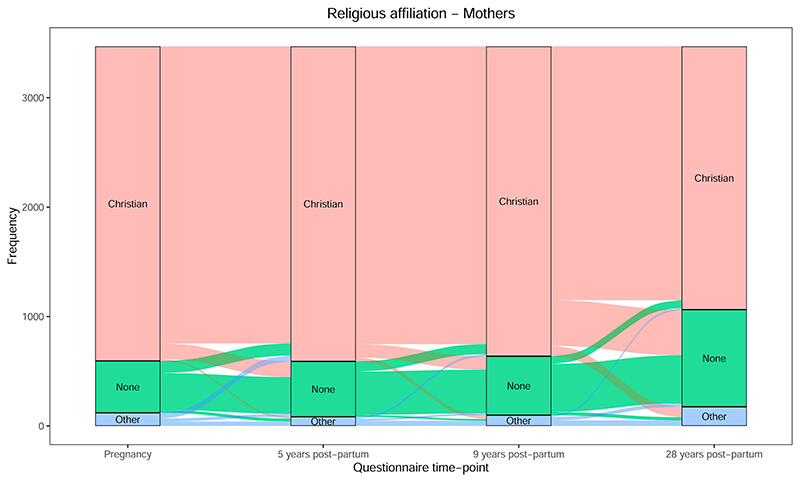
Change in religious affiliation (all Christians grouped together) from pregnancy to 28 years post-partum for mothers (*n* = 3469).

**Figure 3:**
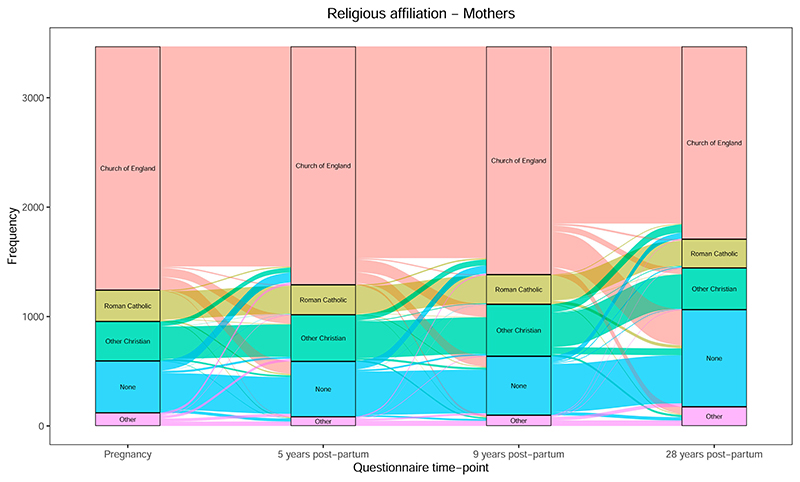
Change in religious affiliation (Christians split into “Church of England,” “Roman Catholic” and “Other Christian”) from pregnancy to 28 years post-partum for mothers (*n* = 3469).

For religious affiliation, we initially coded these responses as Christian, None and Other (Figure 2; Figure S7 for pregnancy to 9 years). The proportion of each was reasonably stable from pregnancy to 9 years, with some fluidity between these different affiliations (i.e., between “None” and “Christian,” “Christian” and “Other,” etc.). By 28 years the proportion of Christians reduced dramatically, as many previous Christians chose either “None” or “Other” at this time-point. When splitting the Christian data by denomination (into “Church of England,” “Roman Catholic” and “Other Christian”), a more nuanced pattern emerged (Figure 3; Figure S8 for pregnancy to 9 years); while there was some interchange between Church of England and Other Christian, the proportion of Roman Catholics remained stable with little in- or out-migration. Additionally, the biggest changes from Christianity to no affiliation were from Church of England, rather than Roman Catholics or Other Christians.

**Figure 4:**
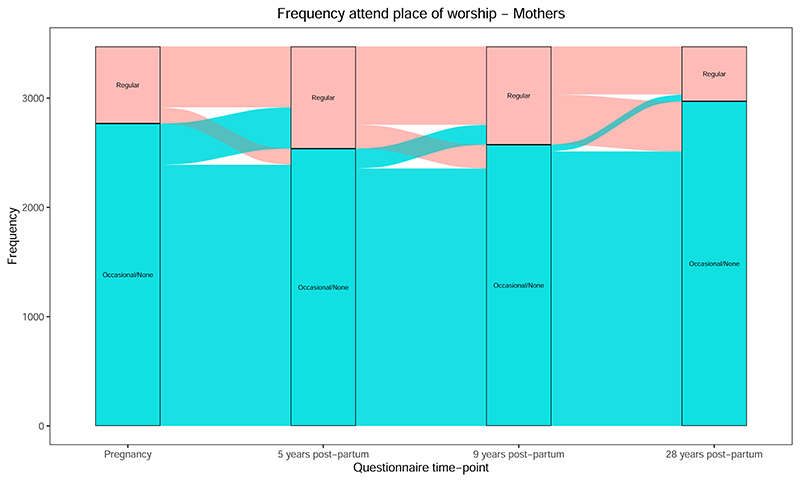
Change in religious attendance (frequency attend a place of worship) from pregnancy to 28 years post-partum for mothers (*n* = 3473).

A different pattern was seen with regular attendance at a place of worship, which increased from pregnancy to 5 years, was stable to 9 years, and then decreased considerably at 28 years (Figure 4; Figure S9 for pregnancy to 9 years). For “length of time had current faith” (Figure S10; Figure S11 for pregnancy to 9 years), there was a slight increase in mothers reporting “>5 years” from “all life” between 9 and 28 years, while the number of mothers reporting current faith for “<5 years” halved between pregnancy and 28 years. The proportion of “yes” responses to “raising study child in said faith,” was stable between 6 and 9 years, but dropped slightly by 28 years (Figure S12). For changes in obtaining help and support from various religious sources, see Figures S13 to S18; overall, these were relatively stable between pregnancy and 9 years, but dropped at 28 years.

**Figure 5:**
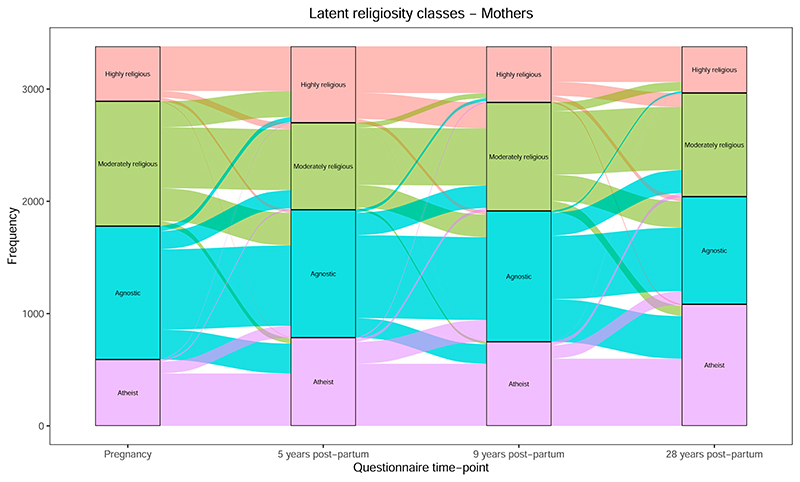
Change in the religiosity latent classes from pregnancy to 28 years post-partum for mothers (*n* = 3381).

Changes in latent classes are displayed in Figure 5 (for pregnancy to 9 years, see Figure S19). Overall, there is an increase in “atheist” classifications over time—especially between 9 and 28 years—with a corresponding decrease in all other categories. Transitions over time were more likely between adjacent categories, e.g., “agnostic” and “atheist,” or “moderately religious” and “highly religious,” again highlighting that shifts in religiosity are predominantly minor rather than major. Despite this general decline in religiosity, at 5 years the size of the “highly religious” class increased compared to in pregnancy; this is most likely because attendance at a place of worship increased from pregnancy to 5 years post-partum (Figure 4), and this variable is strongly weighted to the “highly religious” class. The decline in the “highly religious” group, and expansion of the “moderately religious” class, from 5 to 9 years is more difficult to explain as both attendance at a place of worship (Figure 4) and overall religious belief (Figure 1) were relatively stable over these time-points. This change may be due to differences in the weightings for the latent classes between 5 and 9 years. While the loadings of most variables are broadly consistent across all time-points (Table S1), the loading for “help from religious groups” for the “highly religious” class is lower at 5 years compared to 9 years; this means that being coded as “highly religious” would depend less on this variable at 5 years relative to other ages, and hence an increase in being coded as “highly religious” at this age. Nonetheless, the overall increase in “atheists” and decline in both religiosity and agnosticism from these latent classes is clear.

### Partners’ changes in RSBB over time

Descriptive statistics for partners are presented in Table S3. Although partners were less religious than mothers, patterns of change over time were very similar to those of mothers, so will not be described in detail again here. Sample sizes for partners were also substantially smaller when compared to mothers. For full results, see Figures S20 to S43.

### Illustrative analysis

Descriptive statistics for the two variables summarizing maternal changes in religious belief between pregnancy and 9 years are presented in Table 3. Overall, the majority of mothers (70–80%) were consistent over time. For those that changed, decreases in belief were more common than increases in belief.

We found that sociodemographic factors were associated with the trajectories derived using method 1 (descriptive results in Figures S44–S50; full multinomial results, with “consistent non-believers” as the baseline category, in Table S4). Older age at birth, other than White ethnicity, greater educational attainment, higher income, owning/mortgaging a home (relative to council/housing association) and lower area-level deprivation were all associated with being a “consistent believer,” relative to consistent non-believers. Higher educational attainment, greater income, owning/mortgaging a home (relative to council/housing association) and lower area-level deprivation were associated with being a new non-believer, relative to consistent non-believers. No differences between new believers and consistent non-believers were found. Being a first-time mother was not associated with any of the trajectories.

The method 2 results differed, with fewer associations between the RSBB trajectories reported (although this may in part be due to small numbers who had large gains or losses in belief; descriptive results in Figures S51–S57; full multinomial results in Table S5, with “no change” as the baseline category). Younger age was associated with increases and decreases in religious belief, both large and small. Lower education, lower income, council/housing association accommodation (relative to owned/mortgaged) and higher deprivation were all associated with small increases in religious belief, relative to the baseline category of “no change.” Living in council/housing association and rented accommodation, relative to owned/mortgaged, were both associated with large increases in belief (although cell counts were small—only 11 mothers living in rented accommodation had a large increase in belief, while for council/housing association the corresponding figure was 9—so results should be interpreted with caution). No other factors were associated with large increases, small decreases or large decreases in belief. No associations with ethnicity or being a first-time mother were reported.

## Discussion

We have described individual-level changes in RSBB over nearly a 30-year period in a cohort of UK parents. Consistent with the well-known decline in religiosity and increased secularization of UK society (Office for National Statistics, 2012; Voas & Bruce, 2019), there has been an overall decline in religiosity over time in this population, especially between 9 and 28 years post-partum (approx. 2000–2019). Many other interesting patterns emerged from these data, including; the stability of Catholic affiliation despite a decrease in affiliation of other Christian denominations, somewhat different patterns of change depending on the RSBB measure (e.g., an increase in religious attendance—but not religious belief or affiliation—when the child was young), and the nature of changes in religiosity (e.g., most changes being small-scale, rather than large-scale). While the aim of this paper is predominantly descriptive and to help inform future work, especially regarding religion and health, we will discuss the theoretical implications of some of these results in more detail below, as well as some interesting avenues for future research.

First, these results indicated a decline in religiosity with age. Although the decline in RSBB at a population-level is relatively modest between pregnancy and 9 years—with some interesting exceptions, such as religious attendance—there is a significant drop in religiosity in most measures between 9 and 28 years. This is contrary to previous work, using longitudinal UK data between 1991 and 2001, suggesting that RSBB is relatively stable over adulthood, and that reductions in religiosity are primarily an intergenerational phenomenon (Crockett & Voas, 2006). While requiring replication in independent samples, this suggests that the former stability of religious belief over an adult’s lifespan may no longer hold; if true, this suggests that perhaps something may have changed in recent times (i.e., between 2000 and 2019) to alter this previous stability. Understanding these factors is beyond the scope of this study, but may include factors specific to the study population, such as dependent children leaving the family home (and a concomitant reduction in parental religious adherence), parents adopting the increasingly non-religious norms of their children, and/or wider societal changes occurring at this time, such as increasing exposure to alternative world-views, the continued breakdown of local religious communities, and/or an increasing acceptance of individuals with “no religion” (who may previously have identified as religious (Bullivant, 2019)).

Second, despite overall declines in religiosity, shifts in RSBB are variable and depend on the specific RSBB measure. Taking religious affiliation, for instance, the proportion reporting “Church of England” has declined over time, with changes to and from categories of “other Christian,” “none” and “other religions”; the proportion of Roman Catholics, in contrast, has remained relatively stable, with very few transitions into or out of this faith (Figure 3). One potential explanation for this difference is the additional costly “credibility-enhancing displays” (Henrich, 2009) which the Catholic faith imposes on its followers (baptism, mass, fasting, confession, etc.)—especially when compared to other Christian denominations—which may foster a strong Catholic belief and identity (Finke & Stark, 2005; Iannaccone, 1994). Arguing against this idea, however, is the fact that many of these practices have declined in recent times (since the Second Vatican Council in 1962–1965), and that approximately only one-third of self-identified Catholics attend mass every week (Bullivant, 2019). An alternative explanation may be that, regardless of the specific religious practices and their adherence, Catholic identity is especially ingrained for other reasons, potentially related to identifying as part of a —perhaps historically-persecuted—minority community (e.g., Irish migrants). Distinguishing between these explanations is beyond the scope of this paper, but ALSPAC does have relevant data, including on religious involvement and whether an individual’s ancestors were migrants and where they came from, which can be used to explore this in more detail.

As another example of how these RSBB trajectories are variable, religious attendance increased between pregnancy and 5–9 years, before decreasing at 28 years (Figure 4). This may be due to parents trying to get their child into a faith school—which often have a reputation for good academic achievement (although whether this is warranted is a matter of debate (Gibbons & Silva, 2011))—and usually require parents to attend their local place of worship, regardless of their underlying religious beliefs (which, as we have seen, do not show any such increases over these time-points). ALSPAC is currently processing data on the type of school the child attended, which can be used to test this hypothesis. An alternative reason for this pattern could be that parents participate in religious groups as a source of social support and to help with child-care (Shaver et al., 2020). Regardless of the specific reason, this suggests that different aspects of RSBB may be some-what independent from one another, and perhaps affected by different factors.

This paper has also provided an insight into how RSBB changes over time, with most transitions being small, rather than large-scale (e.g., for belief in God/a divine power, changes between “yes” and “not sure,” or “not sure” and “no,” are much more common than between “yes” and “no”; Figure 1). This pattern corresponds to much previous work suggesting that declines in religiosity are usually gradual, rather than sudden (Bullivant, 2019; Voas, 2009). This interpretation does assume that answers to this religious belief question fall on a continuum of belief from “yes” to “not sure” to “no,” and that shifts between “yes” and “not sure” (or “not sure” and “no”) are minor, while transitions between “yes” and “no” are major. However, it may be argued that “not sure” responses are not a minor change from belief, but rather reflect a growing irrelevance or indifference of religion to individuals’ lives (Bagg & Voas, 2010). Here we assume these responses reflect an ordinal measure of the strength of religious belief. This decision appears somewhat justified by the finding that changes between “yes” and “no” are less common than changes between “yes” and “not sure,” or “not sure” and “no”; while requiring additional exploration, this does suggest that “not sure” may be something of an intermediate state between belief and non-belief. Although the focus of this paper has been on changes in RSBB, it is also important to note that, despite some declines, individual-level RSBBs were relatively stable over the three-decade period observed here, suggesting that religious identities generally do not change dramatically over an individual’s adult lifespan (Crockett & Voas, 2006). While partners were less religious overall than mothers, the patterns of change in RSBB were similar for both. Identifying the reasons behind these patterns is beyond the scope of this paper but does raise interesting questions for future research.

We also conducted an illustrative analysis demonstrating how researchers could analyze these longitudinal data, using religious belief from pregnancy to 9 years in mothers coded in two ways as examples (Tables 2 and 3). These analyses were intentionally simple and largely atheoretical in order to show the kinds of research questions that can be addressed using this data, and results should not be taken as causal estimates without further exploration. Nonetheless, we observed several associations which warrant additional investigation. For instance, relative to “consistent non-believers,” older age was associated with being a “consistent believer,” while higher socioeconomic position was associated with being both a “consistent believer” and a “new non-believer”; none of the factors examined were associated with being a “new believer.” Different RSBB trajectories may therefore be socially and demographically patterned, which may have implications for understanding patterns of secularization. For instance, the observation that individuals from higher socioeconomic positions were more likely to report a decrease in religious belief is consistent with theories of material security which predict a decrease in religiosity with improved living standards (Norris & Inglehart, 2011); although a simple application of this theory is somewhat complicated by evidence that those from higher socioeconomic positions in this population generally had greater overall levels of religiosity at baseline in pregnancy (Major-Smith et al., 2022). Further work is necessary to explore how these and additional factors—such as life events, social factors and cognition/personality (Ingersoll-Dayton et al., 2002; McCullough et al., 2005; Morris Trainor et al., 2019)—may be associated with changes in religiosity over time, whether these associations are causal, and if/how they vary by different aspects of RSBB.

We also acknowledge that these are only two examples of many ways of analyzing these longitudinal data, both of which rest on various assumptions. In method 1, by combining “no” and “not sure” responses together we are assuming that these responses are comparable, while in method 2 the “no change” category assumes that consistent believers and consistent non-believers are comparable. As discussed above, method 2 also assumes that these responses reflect an ordinal measure of religious belief, with “not sure” intermediate between “yes” and “no.” Future work using these data should explore whether such assumptions are plausible and if they impact the study’s results.

These results indicate that, with some exceptions (such as Roman Catholic stability), there is variation in most measures of RSBB over time. It may therefore be possible to employ life-course methods to explore if/how RSBB exposures at different time-points are associated with specific outcomes (Smith et al., 2015, 2016); for instance, these methods could help answer whether there are critical periods for children to adopt the RSBB of their parents, if parental religiosity at specific time-points impacts child health and development, or whether changes in religiosity throughout the life-span impact subsequent health. Given this variation over time, an interesting avenue for future work would be to identify different latent RSBB trajectories over time using longitudinal modeling approaches (Herle et al., 2020), and explore factors associated with these different trajectories.

### Strengths and limitations

A key strength of this research is the nearly 30-year follow-up period (with more RSBB data collections planned) in a large-scale population-based cohort with multiple RSBB measures asked repeatedly. This level of detail of individual-level longitudinal RSBB data is likely unparalleled in a general population cohort. Prospective data collection methods avoid potential biases, such as recall bias (Lash et al., 2021), which may impact other studies relying on retrospective data collection (e.g., (Ingersoll-Dayton et al., 2002)). The wealth of RSBB questions asked also makes it possible to explore different aspects of RSBB and if/how they differ. A further strength is that, with a few exceptions (see section S2 of the supplementary information), all RSBB questions were asked identically at each time-point, making it much easier to examine changes in RSBB over time (not always possible with longitudinal data, see McCullough et al., 2005).

Despite these strengths, there are also important limitations. First, this population reflects a very thin slice of human diversity—namely British, predominantly White, and primarily Christian—and patterns reported here may not generalize to other countries, cultures, ethnicities, religions, or populations. For instance, although a female RSBB bias is found in many Western, Christian countries, this pattern is not universal, and likely depends on sociodemographic factors, the RSBB measure assessed, and other cultural differences (Vardy et al., 2022). Unfortunately, given the demographics of the population it was not possible to explore ethnicities other than White or religions other than Christianity in any more depth than the crude categories of “other than White” and “other religion” adopted here. Different ethnicities and religions are represented in ALSPAC, but their numbers are small and make up less than 5% of the total sample. While broad differences between White and other than White ethnicities, and between Christianity and other religions, are suggestive, given the data available it is not possible to explore these categories at a more granular level. Understanding how and why RSBB changes over the lifespan in a diverse range of societies, religions and ethnicities is therefore a key area for future research. Additionally, as this population only includes parents, it is not clear to what extent these findings are generalizable to adults without children. As the first data collection occurred in pregnancy, the pre-pregnancy RSBB status of these parents is largely unknown (although could be inferred using the question “How long have you had this particular faith?”), meaning it is not clear whether pregnancy and becoming a parent impacts RSBB; it would be possible to assess whether *subsequent* pregnancies are associated with changes in religiosity in this parental cohort, however, while data from the offspring generation can be used to examine whether the transition to parenthood is associated with changes in RSBB.

Second, given that only individuals with complete data for all time-points were included in these analyses, there is the risk that differences in continued participation may result in selection bias (Griffith et al., 2020; Hernán & Robins, 2020; Munafò et al., 2018). This may occur if differences in RSBB, or factors related to RSBB, are associated with continued ALSPAC participation; for instance, if RSBB is associated with continued participation, then this may bias the results reported here towards those with religious beliefs (Morgan et al., 2022). As an example, in the full sample of mothers’ data in pregnancy regarding belief in God/a divine power, 49.9% answered “yes,” 35.3% answered “not sure,” and 14.9% answered “no”; in contrast, of those with complete pregnancy, and 5, 9, and 28 years data, 54.6% answered “yes,” 33.2% answered “not sure,” and 12.3% answered “no.” This suggests that the complete-case sample is somewhat biased towards those with religious beliefs. We attempted to overcome this potential bias by focusing just on data up to 9 years (rather than 28 years), as study attrition would be lower, reducing the risk of selection bias. The majority of results were broadly consistent with those including the 28 years data, but it is possible that selection may have occurred before this 9 year time-point. Whether this selection bias depends on transitions in RSBB—those with a dramatic loss of faith being less likely to continue participating, for instance—is impossible to know from the data here. Future work could use methods such as multiple imputation (Huque et al., 2018; Lee et al., 2021; van Buuren, 2018) or inverse-probability weighting (Seaman & White, 2013) to assess whether complete-case results are robust and attempt to account for potential bias caused by missing data.

Finally, it is unclear to what degree changes in RSBB responses over time are due to actual changes in beliefs/behaviors, as opposed to measurement error, or individuals simply not having a fixed belief or identity (Bullivant, 2019; Lim et al., 2010; Voas, 2009). For example, an agnostic may answer “no” to believing in God/a divine power at one time-point but answer “not sure” at another, without any underlying change in belief; similarly, a non-believing Christian may sometimes report their identity as “Christian,” but other times as “no religion,” without any change in circumstance (Lim et al., 2010). This measurement error has the potential to give the illusion of change occurring over time, and may result in bias (Lash et al., 2021). As data collection was prospective, differential error (i.e., measurement error that depends on values of other variables), which can bias results towards or away from the null, may be less likely, but is still a possibility. If there is non-differential error (i.e., measurement error that does not depend on values of other variables), then results may be biased towards the null. However, these simple heuristics do not apply in every case—for instance, non-differential measurement error in categorical exposures with more than two levels can result in bias away from the null—and quantitative bias analyses can be used as a sensitivity analysis to check the robustness of results to measurement error (Lash et al., 2021; Van Smeden et al., 2020). Relatedly, reported changes in religious affiliation may occur without any underlying change in RSBB due to changing societal factors, which may give the illusion of changes and may also contribute to measurement error. For instance, in the past three decades it has become more acceptable in UK society to identify as having no religion, whereas previously “Church of England” was often a default response, even among atheists or agnostics (Bullivant, 2019). This means that the decline in Christian affiliation (Figure 2) may be due to changing societal attitudes towards those without a religious belief or affiliation, rather than actual changes in religious beliefs and behaviors. As religious beliefs and behaviors appear to have declined over this time period as well, these changing societal norms are unlikely be the sole explanation for the observed decline in religiosity, but may be a contributing factor.

## Conclusion

In this paper, we have described individual-level changes in various RSBB measures over nearly three decades in a cohort of UK parents and provided a simple illustrative example of how these data can be analyzed. Many interesting patterns have been described here—such as the sizeable drop in religiosity between 9 and 28 years post-birth (between 2000 and 2019), the stability of Roman Catholic identity at a time when other Christian faiths are declining, and the apparent increase in religious attendance from pregnancy to 5 and 9 years despite religious belief declining—which can be explored in more detail in future work. We have also provided numerous—but by no means exhaustive—examples of how these data can be used in subsequent research, such as informing theories of secularization, intergenerational transmission of RSBB, life events and RSBB, and associations between religion and health. It is hoped that this paper will inform future research in this area using ALSPAC’s longitudinal RSBB data, and improve our understanding of how religion both shapes, and is shaped by, our lives.

## Supplementary Material

Supplementary Information

## Figures and Tables

**Table 1:** Repeated ALSPAC RSBB variables in the parent generation used in the present study.

		ALSPAC Variable name (mothers; partners)				
Question	Responses	*Pregnancy*	*5 years post-partum*	*6 years post-partum*	*9 years post-partum*	*28 years post-partum*
*Do you believe in God or in some divine power?*	Yes; Not sure; No	d810; pb150	k6240; ph6240	l7040; pj7040	p4040; pm4040	Y3000; FC3000
*Do you feel that God (or some divine power) has helped you at any time?*	Yes; Not sure; No	d811; pb151	k6241; ph6241	l7041; pj7041	p4041; pm4041	Y3010; FC3010
*Would you appeal to God for help if you were in trouble?*	Yes; Not sure; No	d812; pb152	k6242; ph6242	l7042; pj7042	p4042; pm4042	Y3020; FC3020
*Do you “pray” even if not in trouble?*	Yes; No^a^	–	–	l7043; pj7043	p4043; pm4043	Y3030; FC3030
*What sort of religious faith would you say you had?*	None; Christian^b^; Other	d813; pb153	k6243; ph6243	l7044; pj7044	p4044; pm4044	Y3040; FC3040
*How long have you had this particular faith?*	All life; > 5 years; ≤ 5 years	d815; pb154	k6246; ph6246	l7047; ph7047	p4047; pm4047	Y3050; FC3050
*Are you bringing up your child in this faith?* ^c^	Yes; No	–	–	l7048; pj7048	p4048; pm4048	Y3070; FC3070
*Do you go to a place of worship?*	Regular attendance; Occasional/non-attendance^d^	d816; pb155	k6247; ph6247	l7049; pj7049	p4049; pm4049	Y3080; FC3080
*Do you obtain help and support from leaders of your religious group?*	Yes; No^e^	d817; pb156	k6248; ph6248	l7050; pj7050	p4050; pm4050	Y3090; FC3090
*Do you obtain help and support from other members of your religious group?*	Yes; No^e^	d818; pb157	k6249; ph6249	l7051; pj7051	p4051; pm4051	Y3091; FC3091
*Do you obtain help and support from members of other religious groups?*	Yes; No	d819; pb158	k6250; ph6250	l7052; pj7052	p4052; pm4052	Y3093; FC3093

a In the 28 years data collection, a “not sure” option was added to this question.

b In addition to grouping all Christian denominations together, we will also split this group into “Church of England,” “Roman Catholic” and “Other Christian” to explore whether there are changes in self-reported Christian religious affiliation over time.

c In the 28 years data collection, the question changed to past tense: “Did you bring your children up in your current faith/belief (including none)?”

d At all time-points this question had the following response options “Yes, at least once a week,” “Yes, at least once a month,” “Yes, at least once a year” and “Not at all.” However, at 5 years an “Occasional worship” category was added (although it was not chosen by many), at 6 years an “only for special occasions” response was added (this was chosen by many), and at 28 years an “occasionally” response was added (this was chosen by many).

e In the 28 years data collection, a “not applicable” option was added to this question.

**Table 2:** Summary of RSBB trajectories explored in the illustrative analysis examining change in RSBB between pregnancy and 9 years post-partum.

		Belief in God/a divine power in pregnancy		
		Yes	Not sure	No
Belief in God/a divine power 9 years post-partum	Yes	**ConsBel**	**NewBel**	**NewBel**
		=	+	+ +
	Not sure	**NewNon**	**ConsNon**	**ConsNon**
		–	=	+
	No	**NewNon**	**ConsNon**	**ConsNon**
		– –	–	=

Note: The first method of coding is in bold on the first line (ConsBel = consistent believers; ConsNon = consistent non-believers; NewBel = new believers; NewNon = new non-believers), while the second method is in regular font on the lower line (“ = ” = no change; “+” = slight increase; “+ +” = major increase; “–” = slight decrease; “– –” = major decrease).

**Table 3:** Descriptive statistics for the two methods to illustrate how this longitudinal RSBB data could be analyzed (*n* = 7213).

	*N*(%)
*Method 1*	
Consistent non-believers	2865 (39.7%)
Consistent believers	2906 (40.3%)
New believers	587 (8.1%)
New non-believers	855 (11.9%)
*Method 2*	
No change	5088 (70.5%)
Small increase	748 (10.4%)
Large increase	57 (0.8%)
Small decrease	1212 (16.8%)
Large decrease	108 (1.5%)

Note: These derived variables are based on “belief in God/a divine power” from pregnancy and 9 years post-partum for mothers (for details on coding, see Table 2).

## Data Availability

ALSPAC data access is through a system of managed open access. Information about access to ALSPAC data is given on the ALSPAC website (http://www.bristol.ac.uk/alspac/researchers/access/) and in the ALSPAC data management plan (http://www.bristol.ac.uk/alspac/researchers/data-access/documents/alspac-data-management-plan.pdf). Data used for this submission will be made available on request to the Executive (alspac-exec@bristol.ac.uk). The datasets presented in this article are linked to ALSPAC project number B3911, please quote this project number during your application. Analysis code is openly-available on the lead author’s GitHub page: https://github.com/djsmith-90/AnalysisCode_LongitudinalRSBB_B3911.

## References

[R1] Bagg S, Voas D, Zuckerman P (2010). Atheism and secularity (volume 2): Global expressions.

[R2] Boyd A, Golding J, Macleod J, Lawlor DA, Fraser A, Henderson J, Molloy L, Ness A, Ring S, Smith GD (2013). Cohort profile: The “children of the 90s”—the index offspring of the Avon Longitudinal Study of Parents and Children. International Journal of Epidemiology.

[R3] Bruce S (2011). Secularization: In defence of an unfashionable theory.

[R4] Brunson JC, Read QC (2020). ggalluvial: Alluvial plots in “ggplot2” (R package version 0123).

[R5] Bullivant S (2019). Mass exodus: Catholic disaffiliation in Britain and America since Vatican II.

[R6] Cornish RP, Macleod J, Boyd A, Tilling K (2020). Factors associated with participation over time in the Avon Longitudinal Study of Parents and Children: A study using linked education and primary care data. International Journal of Epidemiology.

[R7] Crockett A, Voas D (2006). Generations of decline: Religious change in 20th-century Britain. Journal for the Scientific Study of Religion.

[R8] Finke R, Stark R (2005). The churching of America, 1776–2005: Winners and losers in our religious economy.

[R9] Fraser A, Macdonald-Wallis C, Tilling K, Boyd A, Golding J, Davey Smith G, Henderson J, Macleod J, Molloy L, Ness A, Ring S (2013). Cohort profile: The Avon Longitudinal Study of Parents and Children: ALSPAC mothers cohort. International Journal of Epidemiology.

[R10] Gervais WM, Najle MB, Caluori N (2021). The origins of religious disbelief: A dual inheritance approach. Social Psychological and Personality Science.

[R11] Gibbons S, Silva O (2011). Faith primary schools: Better schools or better pupils?. Journal of Labor Economics.

[R12] Griffith GJ, Morris TT, Tudball MJ, Herbert A, Mancano G, Pike L, Sharp GC, Sterne J, Palmer TM, Smith GD, Tilling K (2020). Collider bias undermines our understanding of COVID-19 disease risk and severity. Nature Communications.

[R13] Halstead I, Heron J, Joinson C (2022). Identifying patterns of religiosity in adults from a large UK cohort using latent class analysis [version 1; peer review: Awaiting peer review]. Wellcome Open Research.

[R14] Harris PA, Taylor R, Thielke R, Payne J, Gonzalez N, Conde JG (2009). Research electronic data capture (REDCap)—a metadata-driven methodology and workflow process for providing translational research informatics support. Journal of Biomedical Informatics.

[R15] Henrich J (2009). The evolution of costly displays, cooperation and religion. Credibility enhancing displays and their implications for cultural evolution. Evolution and Human Behavior.

[R16] Herle M, Micali N, Abdulkadir M, Loos R, Bryant-Waugh R, Hübel C, Bulik CM, De Stavola BL (2020). Identifying typical trajectories in longitudinal data: Modelling strategies and interpretations. European Journal of Epidemiology.

[R17] Hernán MA, Robins J (2020). Causal inference: What if.

[R18] Huque MH, Carlin JB, Simpson JA, Lee KJ (2018). A comparison of multiple imputation methods for missing data in longitudinal studies. BMC Medical Research Methodology.

[R19] Iannaccone LR (1994). Why strict churches are strong. American Journal of Sociology.

[R20] Iles-Caven Y, Bickerstaffe I, Northstone K, Golding J (2021). Spiritual and religious beliefs and behaviour: Data collected from 27/28-year-old offspring in the Avon Longitudinal Study of Parents and Children, 2019–2020. Wellcome Open Research.

[R21] Iles-Caven Y, Gregory S, Bickerstaffe I, Northstone K, Golding J (2021). Parental spiritual and religious beliefs and behaviour data collected from the Avon Longitudinal Study of Parents and Children, 2020. Wellcome Open Research.

[R22] Iles-Caven Y, Gregory S, Northstone K, Golding J (2019). Longitudinal data on parental religious behaviour and beliefs from the Avon Longitudinal Study of Parents and Children (ALSPAC). Wellcome Open Research.

[R23] Ingersoll-Dayton B, Krause N, Morgan D (2002). Religious trajectories and transitions over the life course. International Journal of Aging and Human Development.

[R24] Koenig HG, King D, Carson VB (2012). Handbook of religion and health.

[R25] Lash TL, VanderWeele TJ, Rothman KJ, Rothman KJ, Lash TL, VanderWeele TJ, Haneuse S (2021). Modern epidemiology.

[R26] Lee KJ, Tilling K, Cornish RP, Little RJA, Bell ML, Goetghebeur E, Hogan JW, Carpenter JR (2021). Framework for the treatment and reporting of missing data in observational studies: The TARMOS framework. Journal of Clinical Epidemiology.

[R27] Lim C, MacGregor CA, Putnam RD (2010). Secular and liminal: Discovering heterogeneity among religious nones. Journal for the Scientific Study of Religion.

[R28] Major-Smith D, Morgan J, Halstead I, Tohidinik HR, Iles-Caven Y, Golding J, Northstone K (2022). Demographic and socioeconomic predictors of religious / spiritual beliefs and behaviours in a prospective cohort study (ALSPAC) in southwest England: Results from the parental generation [version 1; peer review: Awaiting peer review]. Wellcome Open Research.

[R29] McCullough ME, Brion SL, Enders CK, Jain AR (2005). The varieties of religious development in adulthood: A longitudinal investigation of religion and rational choice. Journal of Personality and Social Psychology.

[R30] Morgan J, Halstead I, Northstone K, Major-Smith D (2022). Religious/spiritual beliefs and behaviours and study participation in a prospective cohort study (ALSPAC) in southwest England [version 1; peer review: Awaiting peer review]. Wellcome Open Research.

[R31] Morris Trainor Z, Jong J, Bluemke M, Halberstadt J (2019). Death salience moderates the effect of trauma on religiosity. Psychological Trauma: Theory, Research, Practice, and Policy.

[R32] Munafò MR, Tilling K, Taylor AE, Evans DM, Smith GD (2018). Collider scope: When selection bias can substantially influence observed associations. International Journal of Epidemiology.

[R33] Norris P, Inglehart R (2011). Sacred and secular: Religion and politics worldwide.

[R34] Office for National Statistics (2012). Religion in England and Wales.

[R35] R Development Core Team (2021). R: A language and environment for statistical computing.

[R36] Seaman SR, White IR (2013). Review of inverse probability weighting for dealing with missing data. Statistical Methods in Medical Research.

[R37] Shaver JH, Power EA, Purzycki BG, Watts J, Sear R, Shenk MK, Sosis R, Bulbulia JA (2020). Church attendance and alloparenting: An analysis of fertility, social support and child development among English mothers: Church attendance and alloparenting. Philosophical Transactions of the Royal Society B: Biological Sciences.

[R38] Shields AE, Balboni TA (2020). Building towards common psychosocial measures in U.S. Cohort studies: Principal investigators’ views regarding the role of religiosity and spirituality in human health. BMC Public Health.

[R39] Smith ADAC, Hardy R, Heron J, Joinson CJ, Lawlor DA, Macdonald-Wallis C, Tilling K (2016). A structured approach to hypotheses involving continuous exposures over the life course. International Journal of Epidemiology.

[R40] Smith ADAC, Heron J, Mishra G, Gilthorpe MS, Ben-Shlomo Y, Tilling K (2015). Model selection of the effect of binary exposures over the life course. Epidemiology.

[R41] van Buuren S (2018). Flexible imputation of missing data.

[R42] Van Smeden M, Lash TL, Groenwold RHH (2020). Reflection on modern methods: Five myths about measurement error in epidemiological research. International Journal of Epidemiology.

[R43] VanderWeele TJ, Peteet J, Balboni M (2017). Spirituality and religion within the culture of medicine: From evidence to practice.

[R44] VanderWeele TJ, Jackson JW, Li S (2016). Causal inference and longitudinal data: A case study of religion and mental health. Social Psychiatry and Psychiatric Epidemiology.

[R45] Vardy T, Moya C, Placek CD, Apicella CL, Bolyanatz A, Cohen E, Handley C, Kundtová Klocová E, Lesorogol C, Mathew S, McNamara SA (2022). The religiosity gender gap in 14 diverse societies. Religion, Brain & Behavior.

[R46] Voas D (2009). The rise and fall of fuzzy fidelity in Europe. European Sociological Review.

[R47] Voas D, Bruce S (2019). British social attitudes: The 36th report.

