# Supplementary material for: Individual-level changes in religious/spiritual beliefs and behaviors over three decades in the parental generation of the ALSPAC cohort, UK
Source: Religion Brain Behav. Author manuscript; Available in PMC 2024 Jul 22. (PMC7616281; doi:10.1080/2153599X.2022.2156584)
Supplement: Supplementary Information [file EMS197537-supplement-Supplementary_Information.pdf]

Supplementary information for 'Individual-level changes in religious/spiritual beliefs and behaviours over three decades in the parental generation of the ALSPAC cohort, UK'

Supplementary Information Section S1: Differences from pre-registered analysis plan

Overall, the analysis plan was followed as specified. One small deviation from our published protocol was the imputation of missing data. In the analysis plan we said that "based on our previous experience with this data, we will also impute some missing data for certain variables to boost sample sizes (e.g., many RSBB questions were not answered by non-religious participants, so it is possible to impute their data accordingly)."

For each RSBB variable in table 1, other than 'belief in God/a divine power', we explored whether non-religious individuals – i.e., those who said that they did not believe in God/a divine power – were less likely to answer these subsequent questions, potentially because they were less relevant to individuals without a faith or belief. However, after inspecting the data, we decided not to impute any missing data for any of these RSBB variables. Although non-believers were more likely to miss subsequent questions in general, there were still a substantial minority of believers who nonetheless had missing data for these questions; as such, we decided to leave these data as missing, rather than impute as 'non-religious' and risk misclassifying individuals. Other users of these data may decide to impute these missing cases, if they feel it is appropriate.

Supplementary Information Section S2: Coding questions which varied over time

As indicated in the footnotes of table 1, although a number of RSBB questions have been asked repeatedly, in some cases there were minor changes in the response options, meaning that questions were not necessarily presented identically at each time-point. Here we detail some of these changes and the coding decisions made in this study.

Pray even if not in trouble: At 6 and 9 years, the only responses were 'yes' and 'no', while at 28 years an additional 'not sure' option was added. In the analysis plan we said that we may combine these 'not sure' responses with 'no', if this was a valid assumption (e.g., if most of these 'not sure' responses were from non-religious participants and/or answered 'no' to this question at previous time-points). After inspecting the data, we decided not to recode these 'not sure' responses and instead code this data as 'missing', as it was not clear whether recoding these 'not sure' responses to 'no' was a valid assumption. For instance, 307 mothers (6.9% of valid responses) answered 'not sure' to this question at 28 years, the majority of which said they either believed in God/a divine power (*n*=139) or were not sure (*n*=150; only 18 responded 'no'), while there was a relatively even split between 'yes' and 'no' responses to this 'pray even if not in trouble' question from 9 years post-partum (yes = 116; no = 148). Similar patterns were observed for partners.

Attendance at a place of worship: At all time-points this question had the following response options 'Yes, at least once a week', 'Yes, at least once a month', 'Yes, at least once a year' and 'Not at all'. However, at 5 years an 'Occasional worship' category was added (chosen by 11 mothers [0.1% of valid responses] and < 5 partners [< 0.1% of valid responses]), at 6 years an 'only for special occasions' response was added (chosen by 2,742 mothers [33.7% of valid responses] and 1,578 partners [36.4% of valid responses]), and at 28 years an 'occasionally' response was added (chosen by 1,290 mothers [29.2% of valid responses] and 471 partners [22.9% of valid responses]). Given this inconsistency, making it impossible to track changes longitudinally, we decided to recode these responses into a binary variable with 'regular attendance' (attend at least once a week or once a month) vs 'occasional/non-attendance' (attend at least once a year, occasionally, or not at all). This ensured consistency over all time-points, making it possible to trace shifts in religious attendance over time. As we will see below, this also appears to be a valid assumption, as the majority of participants who said 'occasionally' (or variations thereof) were found to have attended a place of worship either not at all or at least once a year when this was measured at the previous time-point.

In an attempt to understand what these 'occasional' categories were measuring, and help users of these data in the future, we will provide some additional detail here. As so few participants answered 'occasional worship' at 5 years, we will focus here on the 6 and 28 years data.

Of 2,742 mothers who answered 'only for special occasions' at 6 years, 1,168 (42.7%) believed in God/a divine power, 1,272 (46.5%) were not sure, while 298 (10.9%) did not believe; these percentages were broadly similar to those of the full 'belief in God/a divine power' sample at this age (yes = 48.2%; not sure = 34.7%; no = 17.1%). Looking next at how these 2,742 mothers who answered 'only for special occasion' at 6 years answered this religious attendance question at 5 years, 1,486 (59.4%) said 'not at all' (relative to 53.4% in the whole sample), 922 (36.8%) said 'at least once a year' (relative to 26.5% in the whole sample), 76 (3.0%) said 'at least once a month' (relative to 9.8% in the whole sample), 15 (0.6%) said 'at least once a week (relative to 10.2% in the whole sample), and <5 (<0.2%) said 'occasional worship' (relative to 0.1% in the whole sample). The

majority of mothers who answered ‘only for special occasions’ at 6 years therefore had a religious belief (or were not sure), and generally attended a place of worship either a minimum of once a year or not at all. Patterns of results were similar for partners.

Of the 1,290 mothers who answered ‘occasionally’ at 28 years, 678 (43.6%) believed in God/a divine power, 428 (33.2%) were not sure, while 182 (14.1%) did not believe; these percentages were broadly similar to those of the full ‘belief in God/a divine power’ sample at this age (yes = 43.6%; not sure = 29.8%; no = 26.6%). Looking next at how these 1,290 mothers who answered ‘occasionally’ at 28 years answered this religious attendance question at 9 years, 322 (28.4%) said ‘not at all’ (relative to 49.8% in the whole sample), 542 (47.8%) said ‘at least once a year’ (relative to 29.0% in the whole sample), 161 (14.2%) said ‘at least once a month’ (relative to 9.2% in the whole sample), and 108 (9.5%) said ‘at least once a week (relative to 11.9% in the whole sample). The majority of mothers who answered ‘occasionally’ at 28 years therefore had a religious belief (or were not sure), and were more likely to have attended a place of worship a minimum of once a year at 9 years. Patterns of results were similar for partners.

Obtain help and support from leaders/other members of own religious group: At 28 years, a ‘not applicable’ option was added to these questions. For mothers, 1,045 (23.8% of valid responses) chose this ‘not applicable’ option regarding obtaining support from leaders of own religious group. Those who answered ‘not applicable’ to this question were more likely to not believe in God/a divine power (57.2% vs 26.6% in whole sample), more likely to have no religious affiliation (63.8% vs 27.0% in whole sample), and more likely to say ‘no’ to obtaining help and support from leaders of own religious group at age 9 (97.7% vs 89.9% in whole sample). Results were comparable for partners to this question, and for both mothers and partners to the ‘obtain help and support from other members of own religious group’ question. As such, and given that if the participant did obtain support they would presumably answer ‘yes’ rather than ‘not applicable’, we decided to code all ‘not applicable’ responses as ‘no’ for both of these help/support questions for mothers and partners.

Table S1: Weightings for each of the variables used to derive the latent RSBB classes, for mothers and partners. These weightings indicate the probability of being categorised in each latent class based on the response to each variable (e.g., mothers in pregnancy classified as "highly religious" had a 100% probability of answering 'yes' to belief in God/a divine power, while mothers in pregnancy classified as "atheist" only had a 5% probability of answering 'yes' to belief in God/a divine power). For 'length of time had this particular faith', categories were coded into 'long-term believer' (had said belief whole life or for more than 5 years) and 'recent convert' (had said belief for 5 years or less). For 'attendance at a place of worship', the same binary categorisation as described in table 1 was used (regular = attend once a week or once a month; occasional/never = attend never, occasionally, or minimum once a year). For 'obtains help from and religious group', affirmative responses to any of the questions 'obtains help/support from members of own religious group', 'obtains help/support from leaders of own religious group' or 'obtains help/support from members of other religious groups' were coded as 'yes'.

**Time-point**	**Latent class**	**Belief in God/a divine power**			**Feel that God has ever helped them**			**Would appeal to God for help in in trouble**			**Length of time had this particular faith**		**Attendance at a place of worship**		**Obtains help from any religious group**	
		Yes	*Not sure*	*No*	*Yes*	*Not sure*	*No*	*Yes*	*Not sure*	*No*	*Long-term*	*Recent*	*Regular*	*Occ./ never*	*Yes*	*No*
**Mothers in pregnancy**	*Highly religious*	1.00	0.00	0.00	0.95	0.05	0.00	0.99	0.01	0.00	0.89	0.11	0.87	0.13	0.83	0.17
	Moderately religious	0.93	0.06	0.00	0.73	0.26	0.02	0.89	0.09	0.01	0.96	0.04	0.09	0.91	0.03	0.97
	Agnostic	0.23	0.75	0.01	0.02	0.78	0.19	0.22	0.71	0.08	1.00	0.00	0.03	0.97	0.02	0.98
	Atheist	0.05	0.25	0.70	0.00	0.01	0.99	0.01	0.09	0.91	0.97	0.03	0.01	0.99	0.01	0.99
**Mothers 5 years post-partum**	*Highly religious*	0.99	0.01	0.00	0.92	0.08	0.00	0.98	0.01	0.00	0.90	0.10	0.93	0.07	0.76	0.24
	Moderately religious	0.92	0.08	0.00	0.66	0.30	0.04	0.90	0.09	0.01	0.97	0.03	0.18	0.82	0.02	0.98
	Agnostic	0.24	0.74	0.02	0.02	0.72	0.26	0.26	0.68	0.05	1.00	0.00	0.07	0.93	0.02	0.98
	Atheist	0.05	0.23	0.73	0.00	0.01	0.99	0.01	0.14	0.85	0.98	0.02	0.01	0.99	0.00	1.00
**Mothers 9 years post-partum**	*Highly religious*	0.99	0.01	0.00	0.94	0.06	0.00	0.98	0.01	0.00	0.92	0.08	0.96	0.04	0.84	0.16
	Moderately religious	0.93	0.07	0.00	0.69	0.28	0.03	0.88	0.10	0.03	0.97	0.03	0.18	0.82	0.04	0.96
	Agnostic	0.22	0.76	0.01	0.02	0.77	0.21	0.23	0.68	0.10	1.00	0.00	0.07	0.93	0.02	0.98
	Atheist	0.05	0.24	0.71	0.00	0.02	0.98	0.01	0.13	0.86	0.98	0.02	0.01	0.99	0.00	1.00
**Mothers 28 years post-partum**	*Highly religious*	1.00	0.00	0.00	0.99	0.01	0.00	0.99	0.01	0.00	0.98	0.02	0.76	0.24	0.92	0.08
	Moderately religious	0.90	0.10	0.00	0.79	0.20	0.01	0.95	0.04	0.01	0.98	0.02	0.01	0.99	0.06	0.94
	Agnostic	0.21	0.77	0.03	0.05	0.70	0.26	0.36	0.55	0.09	1.00	0.00	0.00	1.00	0.02	0.98
	Atheist	0.04	0.17	0.80	0.00	0.01	0.99	0.02	0.09	0.89	0.99	0.01	0.00	1.00	0.01	0.99
**Partners in pregnancy**	*Highly religious*	1.00	0.00	0.00	0.94	0.06	0.00	0.99	0.01	0.00	0.85	0.15	0.90	0.10	0.80	0.20
	Moderately religious	0.90	0.10	0.00	0.74	0.24	0.03	0.83	0.12	0.05	0.95	0.05	0.06	0.94	0.03	0.97
	Agnostic	0.20	0.78	0.03	0.02	0.77	0.22	0.24	0.65	0.10	1.00	0.00	0.03	0.97	0.02	0.98
	Atheist	0.05	0.16	0.79	0.00	0.01	0.99	0.02	0.07	0.91	0.97	0.03	0.01	0.99	0.01	0.99

Time-point	Latent class	Belief in God/a divine power			Feel that God has ever helped them			Would appeal to God for help in in trouble			Length of time had this particular faith		Attendance at a place of worship		Obtains help from any religious group	
		Yes	*Not sure*	*No*	*Yes*	*Not sure*	*No*	*Yes*	*Not sure*	*No*	*Long-term*	*Recent*	*Regular*	*Occ./ never*	*Yes*	*No*
**Partners 5 years post-partum**	*Highly religious*	0.99	0.01	0.00	0.95	0.05	0.00	0.98	0.01	0.00	0.93	0.07	0.92	0.08	0.80	0.20
	Moderately religious	0.91	0.08	0.00	0.72	0.26	0.01	0.87	0.09	0.04	0.96	0.04	0.15	0.85	0.02	0.98
	Agnostic	0.17	0.80	0.03	0.01	0.76	0.23	0.27	0.66	0.07	1.00	0.00	0.06	0.94	0.02	0.98
	Atheist	0.03	0.15	0.82	0.00	0.00	0.99	0.01	0.12	0.86	0.99	0.01	0.01	0.99	0.01	0.99
**Partners 9 years post-partum**	*Highly religious*	0.99	0.01	0.00	0.92	0.07	0.01	0.97	0.02	0.01	0.90	0.10	0.91	0.09	0.93	0.07
	Moderately religious	0.94	0.06	0.00	0.75	0.23	0.02	0.84	0.12	0.04	0.97	0.03	0.19	0.81	0.00	1.00
	Agnostic	0.17	0.81	0.02	0.02	0.77	0.22	0.24	0.66	0.10	1.00	0.00	0.06	0.94	0.02	0.98
	Atheist	0.04	0.13	0.83	0.00	0.01	0.98	0.03	0.11	0.87	0.98	0.02	0.02	0.98	0.01	0.99
**Partners 28 years post-partum**	*Highly religious*	0.99	0.01	0.00	0.96	0.02	0.01	0.98	0.02	0.00	0.99	0.01	0.73	0.27	0.93	0.07
	Moderately religious	0.86	0.13	0.01	0.83	0.15	0.02	0.84	0.12	0.04	0.99	0.01	0.02	0.98	0.03	0.97
	Agnostic	0.20	0.76	0.03	0.01	0.70	0.29	0.28	0.55	0.17	1.00	0.00	0.01	0.99	0.04	0.96
	Atheist	0.03	0.08	0.89	0.00	0.00	1.00	0.01	0.07	0.92	0.98	0.02	0.01	0.99	0.01	0.99

Table S2: Descriptive statistics for all RSBB variables for mothers at each time-point. Results are displayed for the whole sample, for mothers with complete pregnancy to 28 year post-partum data, and for mothers with complete pregnancy to 9 year post-partum data (*n* = 14,157). Note that "Do you 'pray' even if not in trouble?" and "Are you bringing up your child in this faith?" were both first measured at 6 years post-partum, not 5 years.

RSBB variable	Sample	Responses	Pregnancy	5 years post-partum	9 years post-partum	28 years post-partum
Do you believe in God or in some divine power?	Whole sample	Yes	6,066 (49.9%)	4,095 (46.5%)	3,593 (48.3%)	1,942 (43.6%)
		Not sure	4,287 (35.3%)	2,984 (33.9%)	2,545 (24.3%)	1,329 (29.8%)
		No	1,806 (14.9%)	1,729 (19.6%)	1,302 (17.5%)	1,187 (26.6%)
		Total	12,159	8,808	7,440	4,458
	Complete pregnancy to 28 years data	Yes	1,993 (54.6%)	1,826 (50.0%)	1,823 (49.9%)	1,587 (43.4%)
		Not sure	1,211 (33.2%)	1,182 (32.4%)	1,218 (33.3%)	1,107 (30.3%)
		No	449 (12.3%)	645 (17.7%)	612 (16.8%)	959 (26.3%)
		Total	3,653	3,653	3,653	3,653
	Complete pregnancy to 9 years data	Yes	3,435 (52.3%)	3,164 (48.2%)	3,171 (48.3%)	-
		Not sure	2,258 (34.4%)	2,184 (33.2%)	2,254 (34.3%)	-
		No	877 (13.3%)	1,222 (18.6%)	1,145 (17.4%)	-
		Total	6,570	6,570	6,570	-
Do you feel that God (or some divine power) has helped you at any time?	Whole sample	Yes	4,111 (33.9%)	2,640 (30.1%)	2,436 (32.9%)	1,548 (34.8%)
		Not sure	4,599 (37.9%)	3,017 (34.4%)	2,622 (35.4%)	1,138 (25.6%)
		No	3,429 (28.2%)	3,120 (35.5%)	2,349 (31.7%)	1,761 (39.6%)
		Total	12,139	8,777	7,404	4,447
	Complete pregnancy to 28 years data	Yes	1,384 (38.2%)	1,215 (33.6%)	1,261 (34.8%)	1,269 (35.0%)
		Not sure	1,338 (37.0%)	1,223 (33.8%)	1,234 (34.1%)	934 (25.8%)
		No	899 (24.8%)	1,183 (32.7%)	1,126 (31.1%)	1,418 (39.2%)
		Total	3,621	3,621	3,621	3,621
	Complete pregnancy to 9 years data	Yes	2,368 (36.3%)	2,049 (31.4%)	2,152 (33.0%)	-
		Not sure	2,410 (36.9%)	2,233 (34.2%)	2,303 (35.3%)	-
		No	1,748 (26.8%)	2,244 (34.4%)	2,071 (31.7%)	-
		Total	6,526	6,526	6,526	-
Would you appeal to God for help if	Whole sample	Yes	5,651 (46.6%)	4,027 (45.9%)	3,412 (46.0%)	2,161 (48.6%)
		Not sure	3,800 (31.3%)	2,624 (29.9%)	2,173 (29.3%)	864 (19.5%)

RSBB variable	Sample	Responses	Pregnancy	5 years post-partum	9 years post-partum	28 years post-partum
you were in trouble?		No	2,677 (22.1%)	2,124 (24.2%)	1,838 (24.8%)	1,417 (31.9%)
		Total	12,128	8,775	7,423	4,442
	Complete pregnancy to 28 years data	Yes	1,917 (52.9%)	1,822 (50.3%)	1,801 (49.7%)	1,779 (49.1%)
		Not sure	1,031 (28.4%)	999 (27.6%)	991 (27.3%)	705 (19.5%)
		No	676 (18.7%)	803 (22.2%)	832 (23.0%)	1,140 (31.5%)
		Total	3,624	3,624	3,624	3,624
	Complete pregnancy to 9 years data	Yes	3,246 (49.7%)	3,103 (47.5%)	3,031 (46.4%)	-
		Not sure	1,974 (30.2%)	1,889 (28.9%)	1,899 (29.1%)	-
		No	1,312 (20.1%)	1,540 (23.6%)	1,602 (24.5%)	-
		Total	6,532	6,532	6,532	-
Do you 'pray' even if not in trouble?	Whole sample	Yes	-	3,267 (39.6%)	2,867 (39.2%)	1,496 (36.4%)
		No	-	4,988 (60.4%)	4,444 (60.8%)	2,616 (63.6%)
		Total	-	8,255	7,311	4,112
	Complete 6 to 28 years data	Yes	-	1,462 (43.7%)	1,426 (42.7%)	1,221 (36.5%)
		No	-	1,880 (56.3%)	1,916 (57.3%)	2,121 (63.5%)
		Total	-	3,342	3,342	3,342
What sort of religious faith would you say you had? (Christians grouped together)	Whole sample	Christian	9,663 (80.5%)	7,042 (81.4%)	5,878 (80.7%)	2,959 (67.1%)
		None	1,836 (15.3%)	1,340 (15.5%)	1,148 (15.8%)	1,189 (27.0%)
		Other religion	511 (4.3%)	271 (3.1%)	257 (3.5%)	261 (5.9%)
		Total	12,010	8,653	7,283	4,409
	Complete pregnancy to 28 years data	Christian	2,875 (82.9%)	2,879 (83.0%)	2,832 (81.6%)	2,406 (69.4%)
		None	474 (13.7%)	506 (14.6%)	539 (15.5%)	888 (25.6%)
		Other religion	120 (3.5%)	84 (2.4%)	98 (2.8%)	175 (5.0%)
		Total	3,469	3,469	3,469	3,469
	Complete pregnancy to 9 years data	Christian	5,173 (82.3%)	5,196 (82.6%)	5,129 (81.6%)	-
		None	886 (14.1%)	917 (14.6%)	964 (15.3%)	-
		Other religion	229 (3.6%)	175 (2.8%)	195 (3.1%)	-
		Total	6,288	6,288	6,288	-
What sort of religious faith	Whole sample	Church of England	7,726 (64.3%)	5,497 (63.5%)	4,424 (60.7%)	2,157 (48.9%)
		Roman Catholic	998 (8.3%)	679 (7.8%)	556 (7.6%)	334 (7.6%)

RSBB variable	Sample	Responses	Pregnancy	5 years post-partum	9 years post-partum	28 years post-partum
would you say you had? (Christians split)		Other Christian	939 (7.8%)	886 (10.0%)	898 (12.3%)	468 (10.6%)
		None	1,836 (15.3%)	1,340 (15.5%)	1,148 (15.8%)	1,189 (27.0%)
		Other religion	511 (4.3%)	271 (3.1%)	257 (3.5%)	261 (5.9%)
		Total	12,010	8,653	7,283	4,409
	Complete pregnancy to 28 years data	Church of England	2,228 (64.2%)	2,179 (62.8%)	2,086 (60.1%)	1,762 (50.8%)
		Roman Catholic	286 (8.2%)	274 (7.9%)	271 (7.8%)	263 (7.6%)
		Other Christian	361 (10.4%)	426 (12.3%)	475 (13.7%)	381 (11.0%)
		None	474 (13.7%)	506 (14.6%)	539 (15.5%)	888 (25.6%)
		Other religion	120 (3.5%)	84 (2.4%)	98 (2.8%)	175 (5.0%)
		Total	3,469	3,469	3,469	3,469
	Complete pregnancy to 9 years data	Church of England	4,098 (65.2%)	4,040 (64.2%)	3,875 (61.6%)	-
		Roman Catholic	520 (8.3%)	493 (7.8%)	493 (7.8%)	-
		Other Christian	555 (8.8%)	663 (10.5%)	761 (12.1%)	-
		None	886 (14.1%)	917 (14.6%)	964 (15.3%)	-
		Other religion	229 (3.6%)	175 (2.8%)	195 (3.1%)	-
		Total	6,288	6,288	6,288	-
How long have you had this particular faith?	Whole sample	All life	8,770 (81.9%)	6,540 (83.6%)	5,389 (80.7%)	3,238 (74.9%)
		>5 years	1,448 (13.5%)	1,009 (12.9%)	1,088 (16.3%)	1,013 (23.4%)
		≤5 years	496 (4.6%)	270 (3.5%)	199 (3.0%)	74 (1.7%)
		Total	10,714	7,819	6,676	4,325
	Complete pregnancy to 28 years data	All life	2,309 (78.8%)	2,368 (80.8%)	2,347 (80.1%)	2.235 (76.3%)
		>5 years	513 (17.5%)	469 (16.0%)	514 (17.5%)	641 (21.9%)
		≤5 years	109 (3.7%)	94 (3.2%)	70 (2.4%)	55 (1.9%)
		Total	2,931	2,931	2,931	2,931
	Complete pregnancy to 9 years data	All life	4,235 (80.9%)	4,344 (83.0%)	4,268 (81.6%)	-
		>5 years	796 (15.2%)	717 (31.7%)	838 (16.0%)	-
		≤5 years	201 (3.8%)	171 (3.3%)	126 (2.4%)	-
		Total	5,232	5,232	5,232	-
	Whole sample	Yes	-	5,467 (70.1%)	4,911 (71.9%)	2,955 (67.5%)
		No	-	2,332 (29.9%)	1,919 (28.1%)	1,425 (32.5%)

**RSBB variable**	**Sample**	**Responses**	**Pregnancy**	**5 years post-partum**	**9 years post-partum**	**28 years post-partum**
Are you bringing up your child in this faith?		Total	-	7,799	6,830	4,380
	Complete 6 to 28 years data	Yes	-	2,404 (74.5%)	2,411 (74.7%)	2,232 (69.1%)
		No	-	825 (25.5%)	818 (25.3%)	997 (30.9%)
		Total	-	3,229	3,229	3,229
Do you go to a place of worship?	Whole sample	Regular attendance	1,694 (14.3%)	1,709 (20.0%)	1,553 (21.1%)	586 (13.3%)
		Occasional/non-attendance	10,192 (85.7%)	6,821 (80.0%)	5,793 (78.9%)	3,825 (86.7%)
		Total	11,886	8,530	7,346	4,411
	Complete pregnancy to 28 years data	Regular attendance	705 (20.3%)	935 (26.9%)	899 (25.9%)	502 (14.5%)
		Occasional/non-attendance	2,768 (79.7%)	2,538 (73.1%)	2,574 (74.1%)	2,971 (85.5%)
		Total	3,473	3,473	3,473	3,473
	Complete pregnancy to 9 years data	Regular attendance	1,058 (17.0%)	1,407 (22.6%)	1,385 (22.2%)	-
		Occasional/non-attendance	5,175 (83.0%)	4,826 (77.4%)	4,848 (77.8%)	-
		Total	6,233	6,233	6,233	-
Do you obtain help and support from leaders of your religious group?	Whole sample	Yes	887 (7.7%)	634 (7.6%)	704 (10.1%)	401 (9.1%)
		No	10,562 (92.3%)	7,709 (92.4%)	6,294 (89.9%)	3,985 (90.9%)
		Total	11,449	8,343	6,998	4,386
	Complete pregnancy to 28 years data	Yes	334 (10.6%)	314 (9.9%)	379 (12.0%)	295 (9.3%)
		No	2,826 (89.4%)	2,846 (90.1%)	2,781 (88.0%)	2,865 (90.7%)
		Total	3,160	3,160	3,160	3,160
	Complete pregnancy to 9 years data	Yes	503 (8.8%)	470 (8.2%)	573 (10.0%)	-
		No	5,212 (91.2%)	5,245 (91.8%)	5,142 (90.0%)	-
		Total	5,715	5,715	5,715	-
Do you obtain help and support from other members of your religious group?	Whole sample	Yes	1,074 (9.4%)	843 (10.2%)	882 (12.7%)	496 (11.4%)
		No	10,299 (90.6%)	7,421 (89.8%)	6,071 (87.3%)	3,845 (88.6%)
		Total	11,373	8,264	6,953	4,341
		Yes	448 (14.3%)	442 (14.1%)	490 (15.7%)	384 (12.3%)
		No	2,681 (85.7%)	2,687 (85.9%)	2,639 (84.3%)	2,745 (87.7%)

RSBB variable	Sample	Responses	Pregnancy	5 years post-partum	9 years post-partum	28 years post-partum
	Complete pregnancy to 28 years data	Total	3,129	3,129	3,129	3,129
	Complete pregnancy to 9 years data	Yes	658 (11.7%)	657 (11.6%)	725 (12.8%)	-
		No	4,990 (88.3%)	4,991 (88.4%)	4,923 (87.2%)	-
		Total	5,648	5,648	5,648	-
Do you obtain help and support from members of other religious groups?	Whole sample	Yes	232 (2.1%)	143 (1.8%)	175 (2.6%)	62 (1.5%)
		No	10,881 (97.9%)	7.824 (98.2%)	6,525 (97.4%)	4,073 (98.5%)
		Total	11,113	7,967	6,700	4,135
	Complete pregnancy to 28 years data	Yes	56 (2.0%)	54 (2.0%)	70 (2.5%)	28 (1.0%)
		No	2,694 (98.0%)	2,696 (98.0%)	2,680 (97.5%)	2,722 (99.0%)
		Total	2,750	2,750	2,750	2,750
	Complete pregnancy to 9 years data	Yes	93 (1.8%)	78 (1.5%)	115 (2.2%)	-
		No	5,089 (98.2%)	5,104 (98.5%)	5,067 (97.8%)	-
		Total	5,182	5,182	5,182	-
RSBB latent classes	Whole sample	Highly religious	1,151 (10.4%)	1,361 (15.3%)	884 (11.7%)	537 (12.0%)
		Moderately religious	3,536 (31.8%)	2,155 (24.3%)	2,230 (29.4%)	1,255 (28.1%)
		Agnostic	4,159 (37.4%)	3,130 (35.3%)	2,683 (35.4%)	1,220 (27.3%)
		Atheist	2,262 (20.4%)	2,226 (25.1%)	1,788 (23.6%)	1,460 (32.6%)
		Total	11,108	8,872	7,585	4,472
	Complete pregnancy to 28 years data	Highly religious	490 (14.5%)	681 (20.1%)	500 (14.8%)	416 (12.3%)
		Moderately religious	1,112 (32.9%)	775 (22.9%)	967 (28.6%)	923 (27.3%)
		Agnostic	1,118 (35.1%)	1,139 (33.7%)	1,165 (34.5%)	959 (28.4%)
		Atheist	591 (17.5%)	786 (23.2%)	749 (22.2%)	1,083 (32.0%)
		Total	3,381	3,381	3,381	3,381
	Complete pregnancy to 9 years data	Highly religious	733 (12.0%)	1,030 (16.9%)	733 (12.0%)	-
		Moderately religious	2,001 (32.8%)	1,468 (24.1%)	1,770 (29.0%)	-
		Agnostic	2,225 (36.5%)	2,116 (34.7%)	2,175 (35.7%)	-
		Atheist	1,137 (18.7%)	1,482 (24.3%)	1,418 (23.3%)	-
		Total	6,096	6,096	6,096	-

Table S3: Descriptive statistics for all RSBB variables for partners at each time-point. Results are displayed for the whole sample, for partners with complete pregnancy to 28 year post-partum data, and for partners with complete pregnancy to 9 year post-partum data (*n* = 14,157). Note that "Do you 'pray' even if not in trouble?" and "Are you bringing up your child in this faith?" were both first measured at 6 years post-partum, not 5 years.

**RSBB variable**	**Sample**	**Responses**	**Pregnancy**	**5 years post-partum**	**9 years post-partum**	**28 years post-partum**
Do you believe in God or in some divine power?	Whole sample	Yes	3,552 (36.9%)	1,488 (33.5%)	1,209 (35.3%)	622 (30.0%)
		Not sure	3,311 (34.4%)	1,556 (35.1%)	1,121 (32.7%)	508 (24.5%)
		No	2,758 (28.7%)	1,394 (31.4%)	1,093 (31.9%)	942 (45.5%)
		Total	9,621	4,438	3,423	2,072
	Complete pregnancy to 28 years data	Yes	506 (41.1%)	448 (36.4%)	464 (37.7%)	387 (31.4%)
		Not sure	394 (32.0%)	423 (34.4%)	376 (30.5%)	295 (24.0%)
		No	331 (26.9%)	360 (29.2%)	391 (31.8%)	549 (44.6%)
		Total	1,231	1,231	1,231	1,231
	Complete pregnancy to 9 years data	Yes	1,030 (39.0%)	885 (33.5%)	932 (35.3%)	-
		Not sure	900 (34.1%)	951 (36.0%)	858 (32.5%)	-
		No	711 (26.9%)	805 (30.5%)	851 (32.2%)	-
		Total	2,641	2,641	2,641	-
Do you feel that God (or some divine power) has helped you at any time?	Whole sample	Yes	2,425 (25.3%)	1,014 (22.9%)	838 (24.5%)	482 (23.4%)
		Not sure	3,096 (32.3%)	1,416 (32.0%)	1,045 (30.6%)	397 (19.3%)
		No	4,075 (42.5%)	1,998 (45.1%)	1,532 (44.9%)	1,180 (57.3%)
		Total	9,596	4,428	3,415	2,059
	Complete pregnancy to 28 years data	Yes	352 (28.8%)	322 (26.3%)	325 (26.6%)	298 (24.3%)
		Not sure	377 (30.8%)	356 (29.1%)	369 (30.1%)	228 (18.6%)
		No	495 (40.4%)	546 (44.6%)	530 (43.3%)	698 (57.0%)
		Total	1,224	1,224	1,224	1,224
	Complete pregnancy to 9 years data	Yes	693 (26.4%)	612 (23.3%)	645 (24.6%)	-
		Not sure	830 (31.7%)	818 (31.2%)	802 (30.6%)	-
		No	1,099 (41.9%)	1,192 (45.5%)	1,175 (44.8%)	-
		Total	2,622	2,622	2,622	-
Would you appeal to God for help if	Whole sample	Yes	3,461 (36.1%)	1,564 (35.3%)	1,182 (34.8%)	631 (30.6%)
		Not sure	2,640 (27.5%)	1,306 (29.5%)	963 (28.3%)	389 (18.9%)

RSBB variable	Sample	Responses	Pregnancy	5 years post-partum	9 years post-partum	28 years post-partum
you were in trouble?		No	3,493 (36.4%)	1,555 (35.1%)	1,255 (36.9%)	1,043 (50.6%)
		Total	9,594	4,425	3,400	2,063
	Complete pregnancy to 28 years data	Yes	512 (42.1%)	485 (39.9%)	447 (36.8%)	387 (31.8%)
		Not sure	295 (24.3%)	324 (26.6%)	339 (27.9%)	229 (18.8%)
		No	409 (33.6%)	407 (33.5%)	430 (35.4%)	600 (49.3%)
		Total	1,216	1,216	1,216	1,216
	Complete pregnancy to 9 years data	Yes	1,042 (40.0%)	950 (36.4%)	906 (34.8%)	-
		Not sure	686 (26.3%)	751 (28.8%)	745 (28.6%)	-
		No	879 (33.7%)	906 (34.8%)	956 (36.7%)	-
		Total	2,607	2,607	2,607	-
Do you 'pray' even if not in trouble?	Whole sample	Yes	-	1,124 (25.9%)	852 (25.3%)	416 (21.5%)
		No	-	3,216 (74.1%)	2,522 (74.7%)	1,519 (78.5%)
		Total	-	4,340	3,374	1,935
	Complete 6 to 28 years data	Yes	-	350 (29.6%)	335 (28.3%)	274 (23.1%)
		No	-	834 (70.4%)	849 (71.7%)	910 (76.9%)
		Total	-	1,184	1,184	1,184
What sort of religious faith would you say you had? (Christians grouped together)	Whole sample	Christian	6,521 (68.9%)	3,154 (73.0%)	2,366 (71.3%)	1,136 (55.7%)
		None	2,440 (25.8%)	1,040 (24.1%)	837 (25.2%)	829 (40.6%)
		Other religion	506 (5.3%)	127 (2.9%)	117 (3.5%)	75 (3.7%)
		Total	9,467	4,321	3,320	2,040
	Complete pregnancy to 28 years data	Christian	794 (69.8%)	841 (73.9%)	817 (71.8%)	665 (58.4%)
		None	284 (25.0%)	266 (23.4%)	277 (24.3%)	429 (37.7%)
		Other religion	60 (5.3%)	31 (2.7%)	44 (3.9%)	44 (3.9%)
		Total	1,138	1,138	1,138	1,138
	Complete pregnancy to 9 years data	Christian	1,723 (69.8%)	1,830 (74.2%)	1,779 (72.1%)	-
		None	623 (25.3%)	576 (23.3%)	24.6%)	-
		Other religion	121 (4.9%)	61 (2.5%)	82 (3.3%)	-
		Total	2,467	2,467	2,467	-
What sort of religious faith	Whole sample	Church of England	5,194 (54.9%)	2,437 (56.4%)	1,749 (52.7%)	840 (41.2%)
		Roman Catholic	699 (7.4%)	315 (7.3%)	259 (7.8%)	129 (6.3%)

RSBB variable	Sample	Responses	Pregnancy	5 years post-partum	9 years post-partum	28 years post-partum
would you say you had? (Christians split)		Other Christian	628 (6.6%)	402 (9.3%)	358 (10.8%)	167 (8.2%)
		None	2,440 (25.8%)	1,040 (24.1%)	837 (25.2%)	829 (40.6%)
		Other religion	506 (5.3%)	127 (2.9%)	117 (3.5%)	75 (3.7%)
		Total	9,467	4,321	3,320	2,040
	Complete pregnancy to 28 years data	Church of England	584 (51.3%)	621 (54.6%)	593 (52.1%)	494 (43.4%)
		Roman Catholic	88 (7.7%)	77 (7.7%)	92 (8.1%)	72 (6.3%)
		Other Christian	122 (10.7%)	132 (11.6%)	132 (11.6%)	99 (8.7%)
		None	284 (25.0%)	266 (23.4%)	277 (24.3%)	429 (37.7%)
		Other religion	60 (5.3%)	31 (2.7%)	44 (3.9%)	44 (3.9%)
		Total	1,138	1,138	1,138	1,138
	Complete pregnancy to 9 years data	Church of England	1,314 (53.3%)	1,394 (56.5%)	1,321 (53.5%)	-
		Roman Catholic	184 (7.5%)	185 (7.5%)	190 (7.7%)	-
		Other Christian	225 (9.1%)	251 (10.2%)	268 (10.9%)	-
		None	623 (25.3%)	576 (23.3%)	24.6%)	-
		Other religion	121 (4.9%)	61 (2.5%)	82 (3.3%)	-
		Total	2,467	2,467	2,467	-
How long have you had this particular faith?	Whole sample	All life	6.545 (79.0%)	3,017 (78.3%)	2,322 (76.1%)	1,364 (67.8%)
		>5 years	1,385 (16.7%)	737 (19.1%)	647 (21.2%)	615 (30.6%)
		≤5 years	354 (4.3%)	99 (2.6%)	83 (2.7%)	33 (1.6%)
		Total	8,284	3,853	3,052	2,012
	Complete pregnancy to 28 years data	All life	660 (71.1%)	667 (71.9%)	670 (72.2%)	618 (66.6%)
		>5 years	235 (25.3%)	241 (26.0%)	230 (24.8%)	296 (31.9%)
		≤5 years	33 (3.6%)	20 (2.2%)	28 (3.0%)	14 (1.5%)
		Total	928	928	928	928
	Complete pregnancy to 9 years data	All life	1,489 (74.8%)	1,516 (76.2%)	1,501 (75.4%)	-
		>5 years	429 (21.6%)	432 (21.7%)	432 (21.7%)	-
		≤5 years	72 (3.6%)	42 (2.1%)	57 (29.%)	-
		Total	1,990	1,990	1,990	-
	Whole sample	Yes	-	2,414 (60.1%)	1,902 (60.4%)	1,270 (62.7%)
		No	-	1,605 (39.9%)	1,245 (39.6%)	755 (37.3%)

RSBB variable	Sample	Responses	Pregnancy	5 years post-partum	9 years post-partum	28 years post-partum
		Total	-	4,019	3,147	2,025
Are you bringing up your child in this faith?	Complete 6 to 28 years data	Yes	-	703 (64.2%)	698 (63.7%)	709 (64.7%)
		No	-	392 (35.8%)	397 (36.3%)	386 (35.3%)
		Total	-	1,095	1,095	1,095
Do you go to a place of worship?	Whole sample	Regular attendance	976 (10.4%)	633 (14.7%)	536 (15.9%)	235 (11.4%)
		Occasional/non-attendance	8,447 (89.6%)	3,655 (85.3%)	2,844 (84.1%)	1,820 (88.6%)
		Total	9.423	4,298	3,380	2,055
	Complete pregnancy to 28 years data	Regular attendance	219 (18.6%)	260 (22.1%)	269 (22.8%)	168 (14.3%)
		Occasional/non-attendance	959 (81.4%)	918 (77.9%)	909 (77.2%)	1,010 (85.7%)
		Total	1,178	1,178	1,178	1,178
	Complete pregnancy to 9 years data	Regular attendance	376 (15.0%)	433 (17.2%)	442 (17.6%)	-
		Occasional/non-attendance	2,136 (85.0%)	2,079 (82.8%)	2,070 (82.4%)	-
		Total	2,512	2,512	2,512	-
Do you obtain help and support from leaders of your religious group?	Whole sample	Yes	542 (5.9%)	297 (7.1%)	278 (8.4%)	171 (8.4%)
		No	8,569 (94.1%)	3,908 (92.9%)	3,032 (91.6%)	1,876 (91.6%)
		Total	9,111	4,205	3,310	2,047
	Complete pregnancy to 28 years data	Yes	114 (10.3%)	120 (10.9%)	129 (11.7%)	115 (10.4%)
		No	991 (89.7%)	985 (89.1%)	976 (88.3%)	990 (89.6%)
		Total	1,105	1,105	1,105	1,105
	Complete pregnancy to 9 years data	Yes	199 (8.4%)	204 (8.7%)	223 (9.5%)	-
		No	2,159 (91.6%)	2,154 (91.3%)	2,135 (90.5%)	-
		Total	2,358	2,358	2,358	-
Do you obtain help and support from other members of your religious group?	Whole sample	Yes	629 (7.0%)	330 (7.9%)	319 (9.7%)	193 (9.5%)
		No	8,394 (93.0%)	3,855 (92.1%)	2,982 (90.3%)	1,833 (90.5%)
		Total	9,023	4,185	3,301	2,026
		Yes	134 (12.4%)	124 (11.4%)	150 (13.8%)	119 (11.0%)
		No	951 (87.6%)	961 (88.6%)	935 (86.2%)	966 (89.0%)

RSBB variable	Sample	Responses	Pregnancy	5 years post-partum	9 years post-partum	28 years post-partum
	Complete pregnancy to 28 years data	Total	1,085	1,085	1,085	1,085
	Complete pregnancy to 9 years data	Yes	232 (10.0%)	209 (9.0%)	245 (10.5%)	-
		No	2,093 (90.0%)	2,116 (91.0%)	2,080 (89.5%)	-
		Total	2,325	2,325	2,325	-
Do you obtain help and support from members of other religious groups?	Whole sample	Yes	136 (1.5%)	65 (1.6%)	52 (1.6%)	27 (1.4%)
		No	8.787 (98.5%)	4,050 (98.4%)	3,188 (98.4%)	1,912 (98.6%)
		Total	8,923	4,115	3,240	1,939
	Complete pregnancy to 28 years data	Yes	27 (2.7%)	22 (2.2%)	15 (1.5%)	10 (1.0%)
		No	963 (97.3%)	968 (97.8%)	975 (98.5%)	980 (99.0%)
		Total	990	990	990	990
	Complete pregnancy to 9 years data	Yes	44 (2.0%)	37 (1.7%)	36 (1.6%)	-
		No	2,176 (98.0%)	2,183 (98.3%)	2,184 (98.4%)	-
		Total	2,220	2,220	2,220	-
RSBB latent classes	Whole sample	Highly religious	746 (9.1%)	492 (11.0%)	332 (9.5%)	202 (9.7%)
		Moderately religious	1,783 (21.7%)	752 (16.8%)	686 (19.7%)	345 (16.5%)
		Agnostic	2,714 (33.0%)	1,550 (34.6%)	1,120 (32.2%)	475 (22.8%)
		Atheist	2,976 (36.2%)	1,683 (37.6%)	1,340 (38.5%)	1,065 (51.0%)
		Total	8,219	4,477	3,478	2,087
	Complete pregnancy to 28 years data	Highly religious	155 (14.6%)	168 (15.8%)	138 (13.0%)	121 (11.4%)
		Moderately religious	222 (20.9%)	162 (15.3%)	188 (17.7%)	165 (15.5%)
		Agnostic	315 (29.7%)	348 (32.8%)	338 (31.8%)	241 (22.7%)
		Atheist	370 (34.8%)	384 (36.2%)	398 (37.5%)	535 (50.4%)
		Total	1,062	1,062	1,062	1,062
	Complete pregnancy to 9 years data	Highly religious	272 (11.9%)	281 (12.3%)	237 (10.4%)	-
		Moderately religious	482 (21.2%)	347 (15.2%)	425 (18.7%)	-
		Agnostic	721 (31.7%)	798 (35.0%)	725 (31.8%)	-
		Atheist	802 (35.2%)	851 (37.4%)	890 (39.1%)	-
		Total	2,277	2,277	2,277	-

Table S4: Summary of multinomial regression results for the method 1 illustrative analysis based on 'belief in God/a divine power' from pregnancy and 9 years post-partum for mothers (for details on coding, see table 2). For all analyses, the baseline reference outcome is 'consistent non-believers' ('no' or 'not sure' at both time-points). ConsBel = Consistent believers ('yes' at both time-points); NewBel = New believers ('no' or 'not sure' in pregnancy & 'yes' 9 years post-partum); NewNon = New non-believers ('yes' in pregnancy & 'no' or 'not' sure 9 years post-partum). The 'p_exp' column gives the overall *p*-value assessing whether the exposure is associated with the outcome, using a likelihood ratio test comparing the model with vs without the exposure. Other than the age at birth model, all models are adjusted for age. RRR = relative risk ratio; CI = confidence interval; HA = Housing association; IMD = index of multiple deprivation.

**Exposure**	**exposure level**	**outcome level**	**RRR**	**Lower 95% CI**	**Upper 95% CI**	***p*-value**	**p_exp**	***n***
Age at birth (years)	NA	ConsBel	1.04	1.03	1.05	<0.001	<0.001	7,213
		NewBel	1.00	0.98	1.02	0.920		
		NewNon	1.00	0.98	1.01	0.768		
Ethnicity (ref=White)	Other than White	ConsBel	3.21	1.98	5.20	<0.001	<0.001	7,064
		NewBel	1.79	0.79	4.05	0.159		
		NewNon	1.54	0.73	3.27	0.260		
Education (ref=CSE/None)[a]	Vocational	ConsBel	1.45	1.15	1.84	0.002	<0.001	7,083
	O level		1.60	1.34	1.90	<0.001		
	A level		1.95	1.62	2.34	<0.001		
	Degree		1.61	1.32	1.96	<0.001		
	Vocational	NewBel	0.85	0.58	1.24	0.404		
	O level		0.91	0.70	1.19	0.502		
	A level		0.98	0.74	1.3	0.876		
	Degree		0.74	0.53	1.02	0.069		
	Vocational	NewNon	1.25	0.88	1.77	0.209		
	O level		1.52	1.18	1.95	0.001		
	A level		1.77	1.36	2.31	<0.001		
	Degree		1.05	0.77	1.44	0.755		
Income (log GBP)	NA	ConsBel	1.15	1.02	1.29	0.026	0.029	6,649
		NewBel	0.96	0.78	1.18	0.689		
		NewNon	1.22	1.02	1.45	0.027		

Exposure	exposure level	outcome level	RRR	Lower 95% CI	Upper 95% CI	*p*-value	p_exp	*n*
Home Ownership (ref=Owned/Mortgaged)	Rented	ConsBel	0.90	0.71	1.13	0.362	<0.001	7,088
	Council/HA		0.57	0.47	0.69	<0.001		
	Other		1.03	0.74	1.43	0.853		
	Rented	NewBel	1.16	0.80	1.68	0.443		
	Council/HA		0.90	0.67	1.21	0.482		
	Other		1.09	0.64	1.88	0.742		
	Rented	NewNon	0.88	0.63	1.24	0.466		
	Council/HA		0.61	0.46	0.81	0.001		
	Other		0.52	0.29	0.95	0.034		
IMD (ref=Quin. 1/Least deprived)	Quintile 2	ConsBel	0.83	0.71	0.96	0.012	0.001	6,651
	Quintile 3		0.90	0.76	1.05	0.180		
	Quintile 4		0.79	0.67	0.93	0.006		
	Quin. 5/Most dep.		0.66	0.54	0.80	<0.001		
	Quintile 2	NewBel	0.96	0.74	1.23	0.738		
	Quintile 3		0.82	0.62	1.09	0.174		
	Quintile 4		0.88	0.66	1.17	0.384		
	Quin. 5/Most dep.		0.86	0.63	1.19	0.368		
	Quintile 2	NewNon	0.76	0.61	0.95	0.015		
	Quintile 3		0.76	0.60	0.96	0.021		
	Quintile 4		0.72	0.56	0.91	0.007		
	Quin. 5/Most dep.		0.55	0.41	0.74	<0.001		
First-time Mother (ref=No)	New Mother	ConsBel	1.05	0.95	1.18	0.343	0.374	7,053
		NewBel	1.00	0.83	1.20	0.984		
		NewNon	1.15	0.97	1.35	0.098		

[a] CSE = Certificate of Secondary Education qualifications; examinations sat at the end of secondary school at approx. age 16; O-level = Ordinary level qualifications; examinations sat at the end of secondary school, often for more academically-able pupils, at approx. age 16; A-level = Advanced level qualifications; non-compulsory examinations sat at the end of college or sixth form at approx. age 18.

Table S5: Summary of multinomial regression results for the method 2 illustrative analysis based on 'belief in God/a divine power' from pregnancy and 9 years post-partum for mothers (for details on coding, see table 2). For all analyses, the baseline reference outcome is 'no change' (same response at both time-points). "+" = Small increase ('no' in pregnancy & 'not sure' 9 years post-partum, or 'not sure' in pregnancy & 'yes' 9 years post-partum); "+ +" = Large increase ('no' in pregnancy & 'yes' 9 years post-partum); "-" = Small decrease ('yes' in pregnancy & 'not sure' 9 years post-partum, or 'not sure' in pregnancy & 'no' 9 years post-partum); "- -" = Large decrease ('yes' in pregnancy & 'no' 9 years post-partum). The 'p_exp' column gives the overall *p*-value assessing whether the exposure is associated with the outcome, using a likelihood ratio test comparing the model with vs without the exposure. Other than the age at birth model, all models are adjusted for age. RRR = relative risk ratio; CI = confidence interval; HA = Housing association; IMD = index of multiple deprivation.

**Exposure**	**exposure level**	**outcome level**	**RRR**	**Lower 95% CI**	**Upper 95% CI**	***p*-value**	**p_exp**	***n***
Age at birth (years)	NA	+	0.96	0.95	0.98	<0.001	<0.001	7213
		+ +	0.93	0.88	0.99	0.015		
		-	0.97	0.96	0.99	<0.001		
		- -	0.94	0.90	0.98	0.005		
Ethnicity (ref=White)	Other than White	+	0.89	0.47	1.67	0.712	0.348	7,064
		+ +	1.07	0.15	7.80	0.950		
		-	0.54	0.29	1.01	0.054		
		- -	1.15	0.28	4.75	0.843		
Education (ref=CSE/None)[a]	Vocational	+	0.64	0.46	0.89	0.008	0.001	7,083
	O level		0.69	0.55	0.86	0.001		
	A level		0.59	0.46	0.76	<0.001		
	Degree		0.57	0.43	0.76	<0.001		
	Vocational	+ +	1.18	0.46	3.01	0.734		
	O level		0.49	0.22	1.11	0.088		
	A level		0.73	0.32	1.68	0.463		
	Degree		0.81	0.31	2.08	0.658		
	Vocational	-	0.98	0.74	1.30	0.916		
	O level		1.05	0.86	1.29	0.634		
	A level		0.96	0.77	1.19	0.703		
	Degree		0.75	0.59	0.97	0.025		

Exposure	exposure level	outcome level	RRR	Lower 95% CI	Upper 95% CI	*p*-value	p_exp	*n*
	Vocational	- -	0.90	0.42	1.93	0.789		
	O level		0.78	0.44	1.36	0.376		
	A level		0.65	0.35	1.21	0.171		
	Degree		0.48	0.22	1.03	0.061		
Income (log GBP)	NA	+	0.80	0.67	0.96	0.014	0.048	6,649
		+ +	0.61	0.34	1.09	0.096		
		-	1.04	0.90	1.20	0.610		
		- -	1.10	0.71	1.72	0.663		
Home Ownership (ref=Owned/Mortgaged)	Rented	+	0.97	0.69	1.38	0.880	0.004	7,088
	Council/HA		1.41	1.10	1.81	0.007		
	Other		1.01	0.63	1.62	0.975		
	Rented	+ +	4.40	2.16	8.97	<0.001		
	Council/HA		2.24	1.03	4.85	0.041		
	Other		0.78	0.11	5.83	0.812		
	Rented	-	0.86	0.64	1.15	0.302		
	Council/HA		1.05	0.84	1.32	0.669		
	Other		0.70	0.45	1.09	0.115		
	Rented	- -	1.70	0.86	3.35	0.127		
	Council/HA		1.37	0.74	2.54	0.312		
	Other		0.67	0.16	2.76	0.575		
IMD (ref=Quin. 1/Least deprived)	Quintile 2	+	1.28	1.02	1.60	0.030	0.011	6,651
	Quintile 3		1.07	0.83	1.37	0.609		
	Quintile 4		1.23	0.96	1.57	0.107		
	Quin. 5/Most dep.		1.62	1.24	2.12	<0.001		
	Quintile 2	+ +	0.29	0.10	0.86	0.026		
	Quintile 3		0.63	0.26	1.54	0.314		
	Quintile 4		1.65	0.83	3.27	0.154		
	Quin. 5/Most dep.		1.02	0.41	2.53	0.967		

**Exposure**	**exposure level**	**outcome level**	**RRR**	**Lower 95% CI**	**Upper 95% CI**	***p*-value**	**p_exp**	***n***
	Quintile 2	-	0.96	0.81	1.15	0.672		
	Quintile 3		0.94	0.77	1.14	0.507		
	Quintile 4		0.92	0.75	1.13	0.427		
	Quin. 5/Most dep.		0.90	0.71	1.14	0.377		
	Quintile 2	- -	1.14	0.65	2.00	0.643		
	Quintile 3		1.02	0.55	1.89	0.961		
	Quintile 4		1.28	0.71	2.32	0.413		
	Quin. 5/Most dep.		1.17	0.58	2.37	0.664		
First-time Mother (ref=No)	New Mother	+	0.91	0.77	1.07	0.245	0.813	7,053
		+ +	1.10	0.63	1.91	0.744		
		-	0.98	0.86	1.12	0.808		
		- -	1.05	0.70	1.56	0.827		

[a] CSE = Certificate of Secondary Education qualifications; examinations sat at the end of secondary school at approx. age 16; O-level = Ordinary level qualifications; examinations sat at the end of secondary school, often for more academically-able pupils, at approx. age 16; A-level = Advanced level qualifications; non-compulsory examinations sat at the end of college or sixth form at approx. age 18.

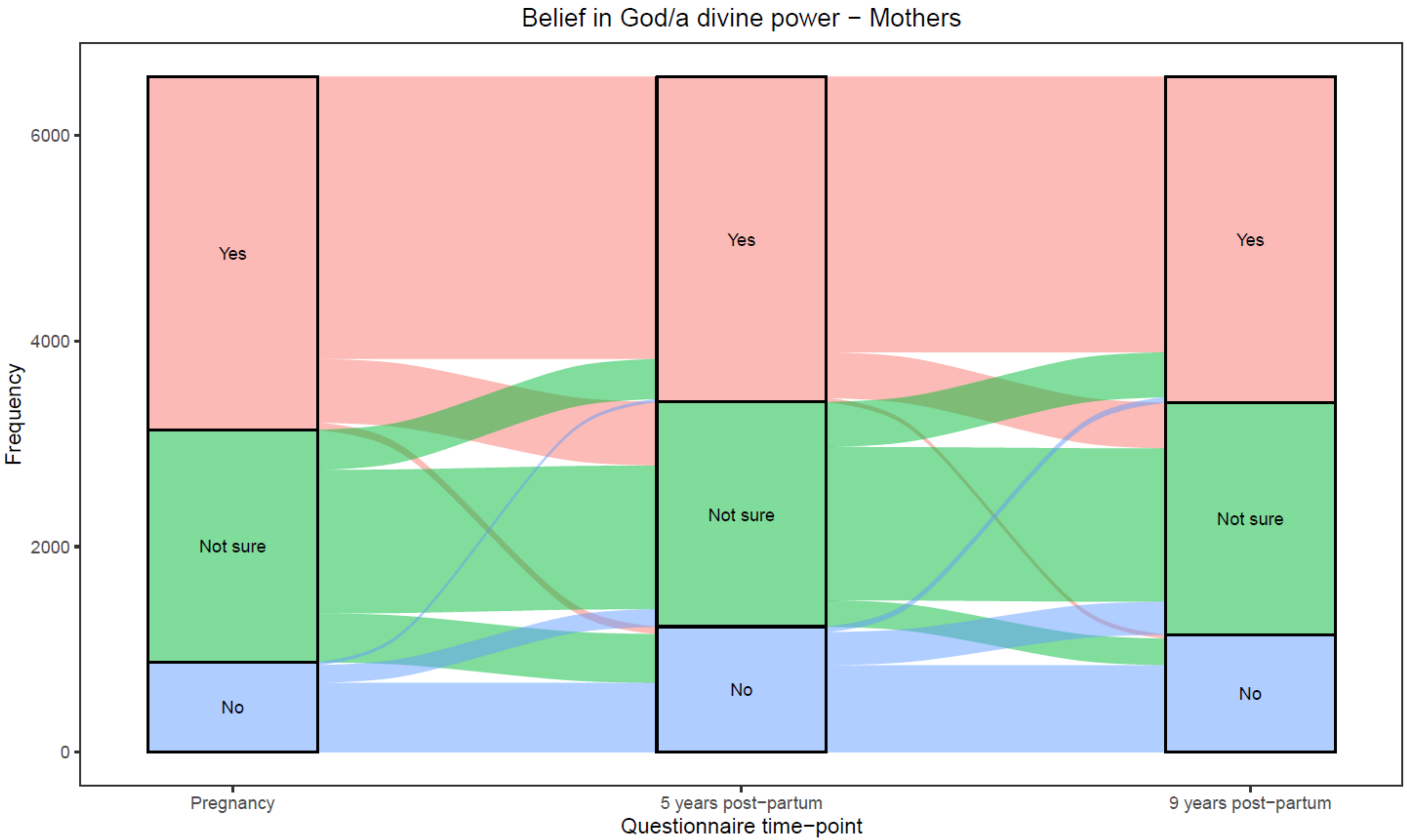

Figure S1: Change in belief in God/a divine power from pregnancy to 9 years post-partum for mothers (*n* = 6,570).

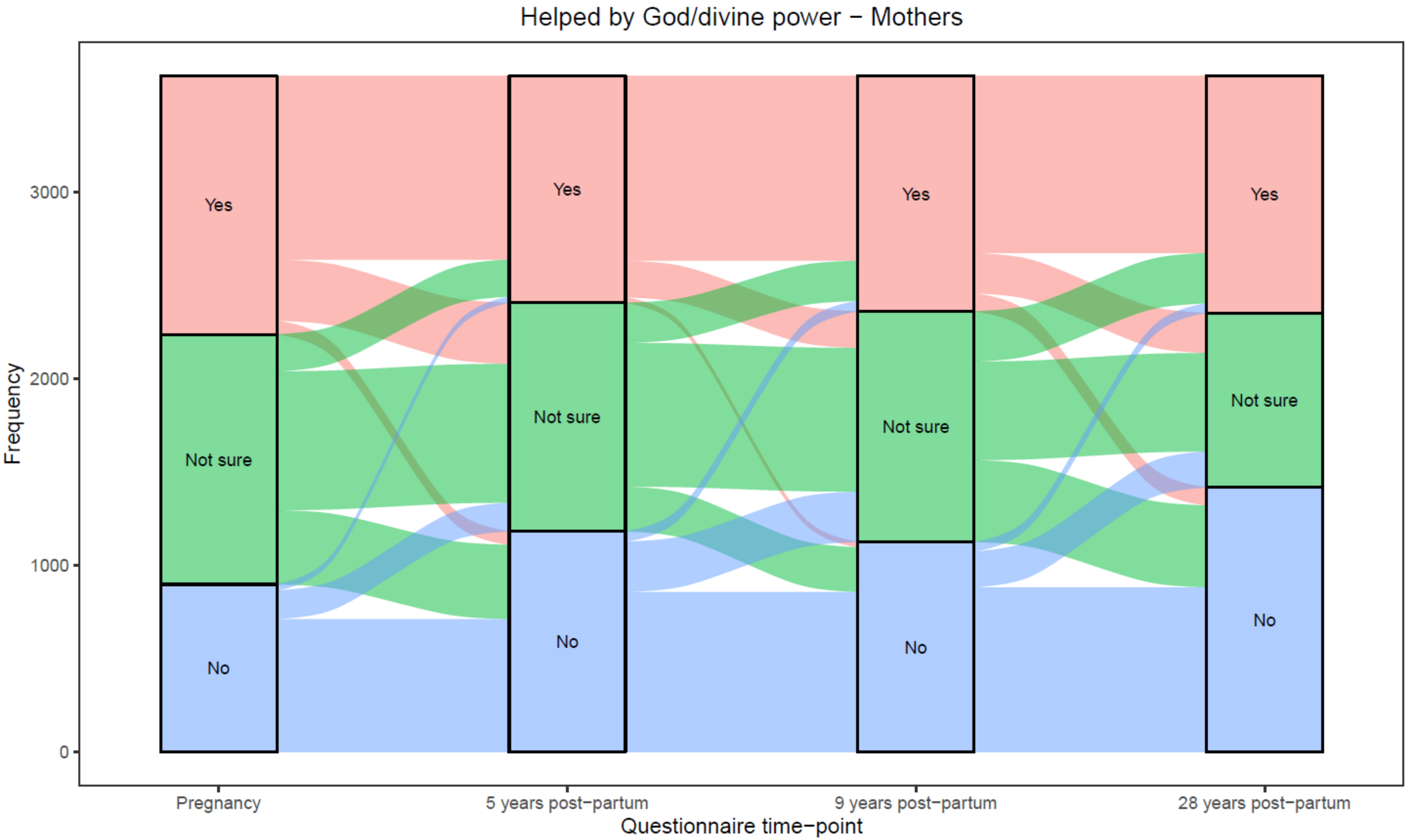

Figure S2: Change in 'ever helped by God/a divine power' from pregnancy to 28 years post-partum for mothers (*n* = 3,621).

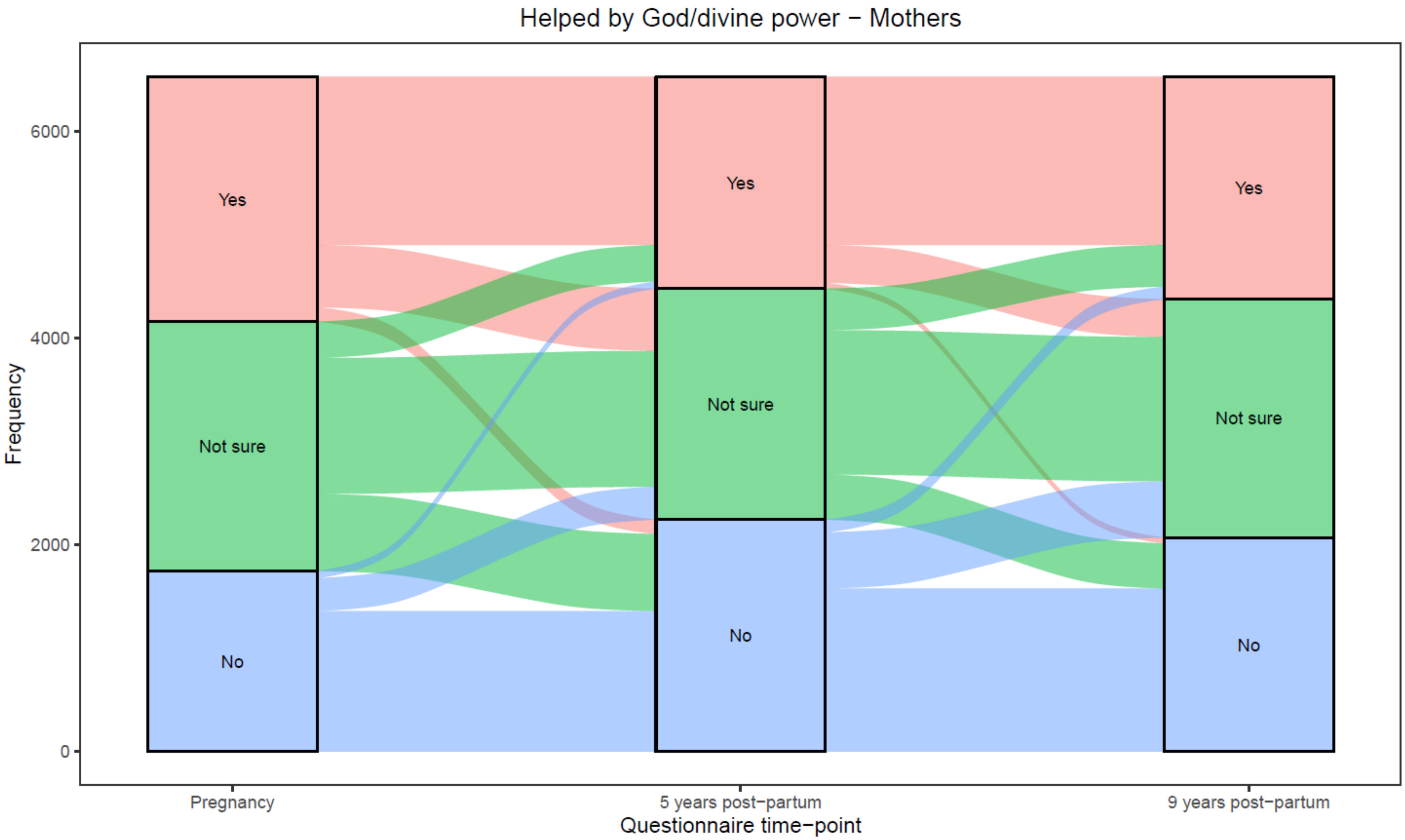

Figure S3: Change in ‘ever helped by God/a divine power’ from pregnancy to 9 years post-partum for mothers (*n* = 6,526).

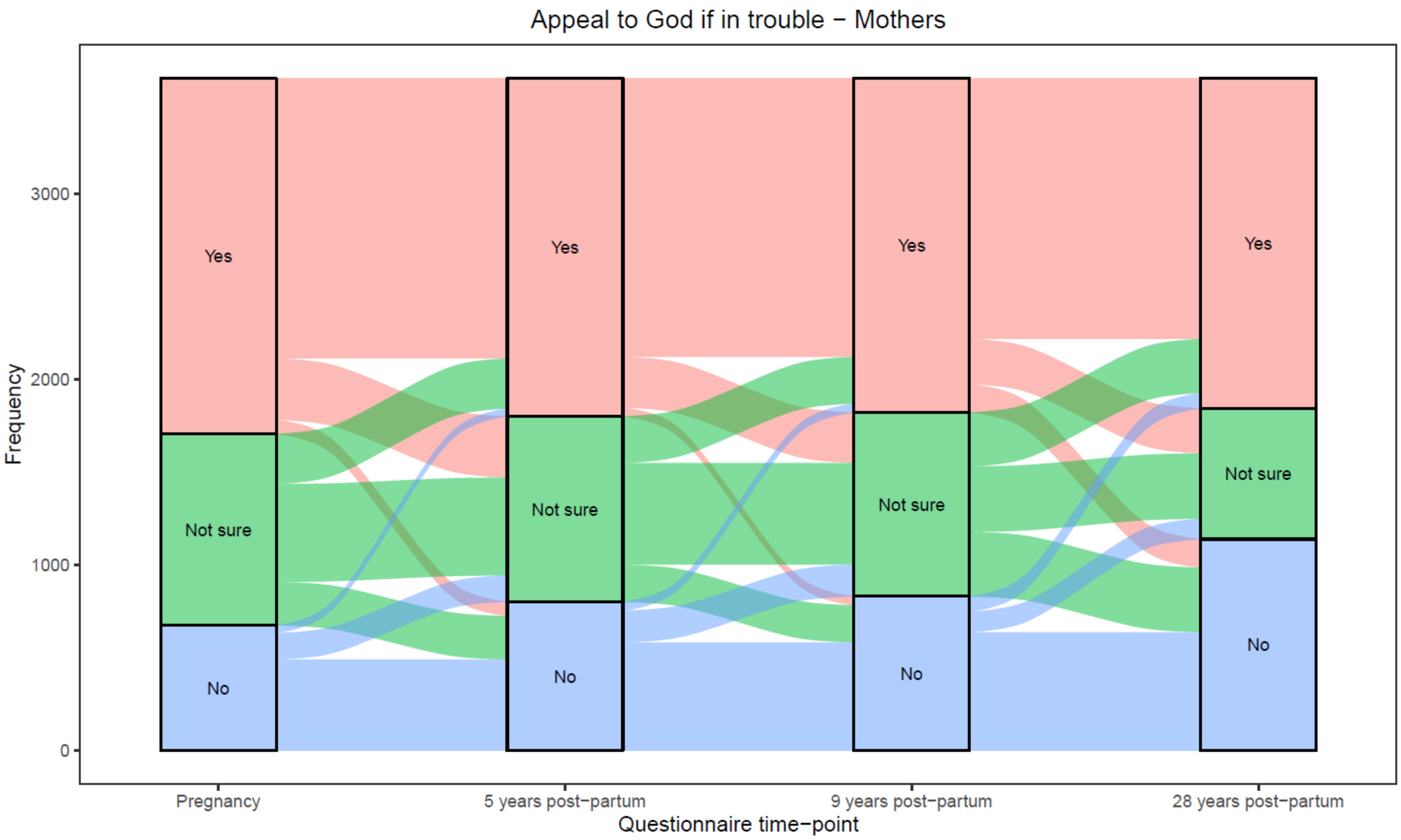

Figure S4: Change in 'would appeal to God for help if in trouble' from pregnancy to 28 years post-partum for mothers (*n* = 3,624).

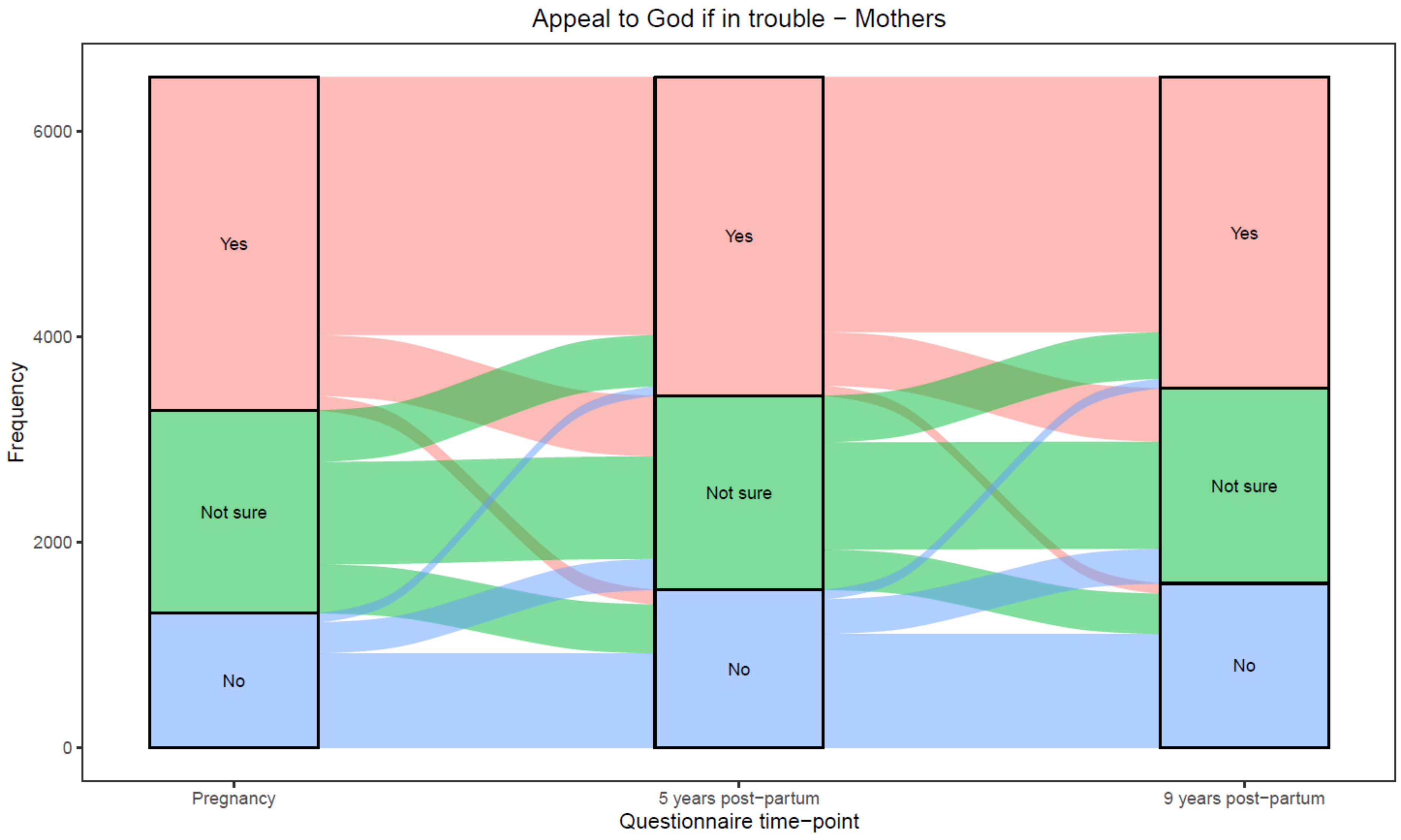

Figure S5: Change in 'would appeal to God for help if in trouble' from pregnancy to 9 years post-partum for mothers (*n* = 6,532).

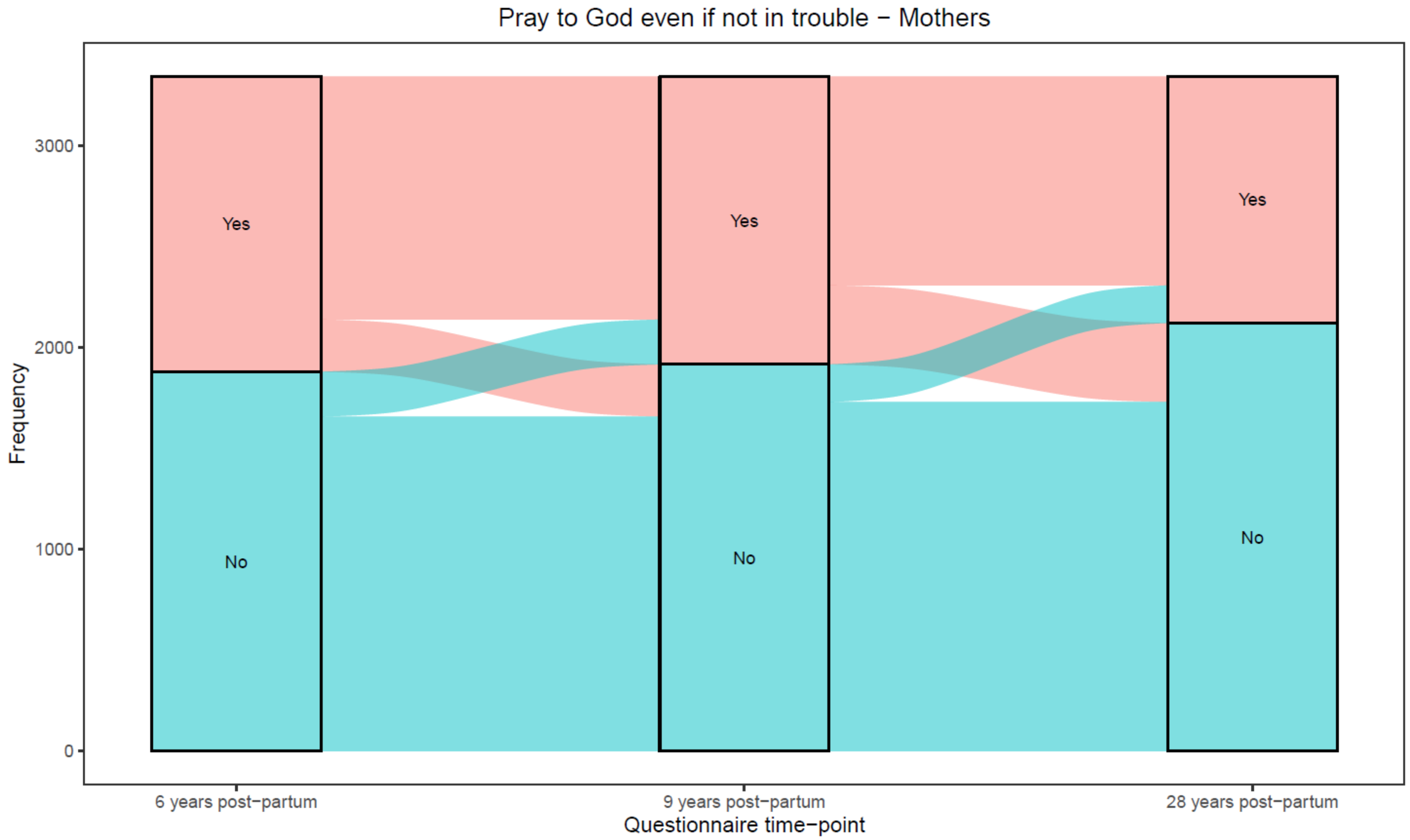

Figure S6: Change in 'would pray, even if not in trouble' from 6 to 28 years post-partum for mothers (*n* = 3,342).

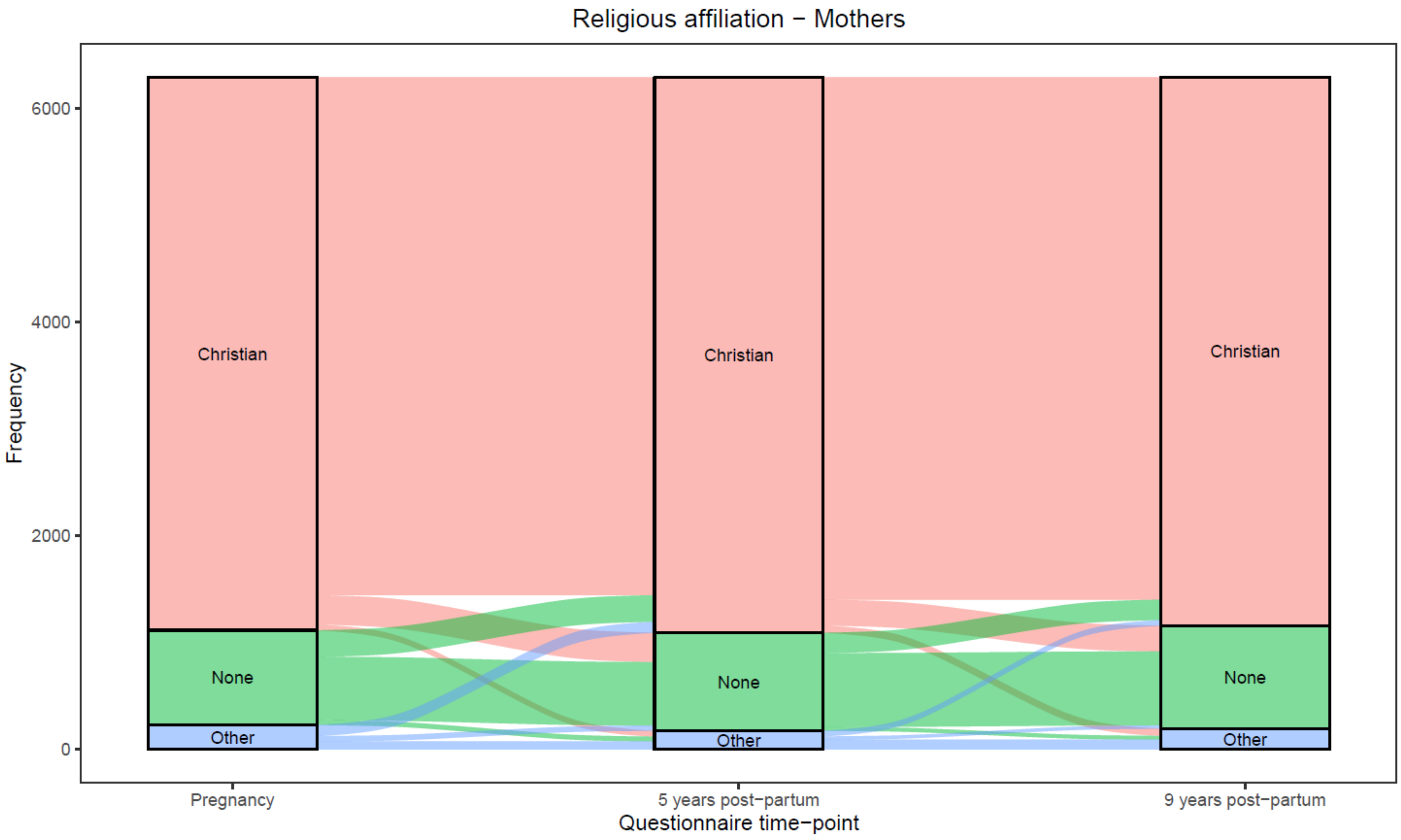

Figure S7: Change in religious affiliation (all Christians grouped together) from pregnancy to 9 years post-partum for mothers (*n* = 6,288).

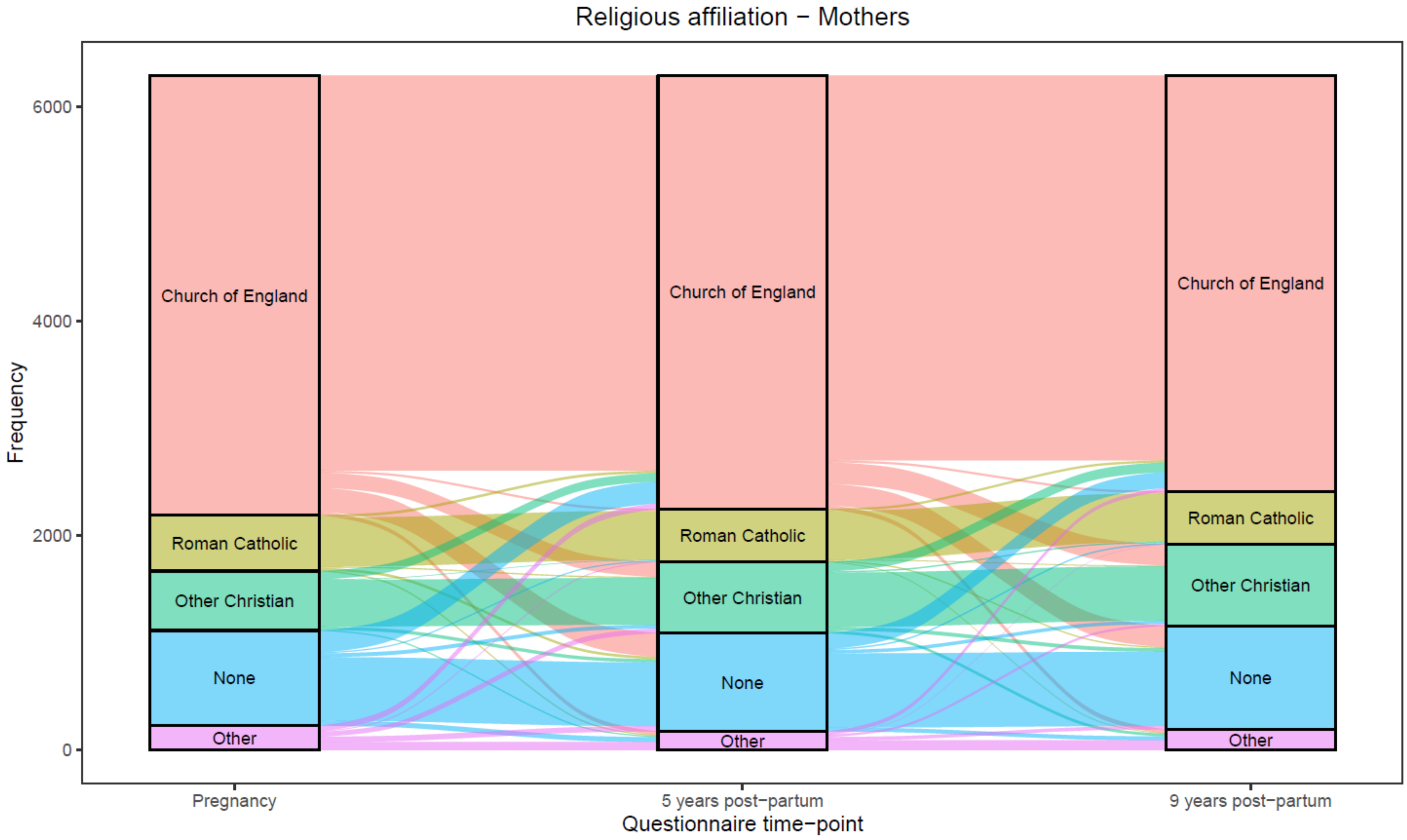

Figure S8: Change in religious affiliation (Christians split into 'Church of England', 'Roman Catholic' and 'Other Christian') from pregnancy to 9 years post-partum for mothers (*n* = 6,288).

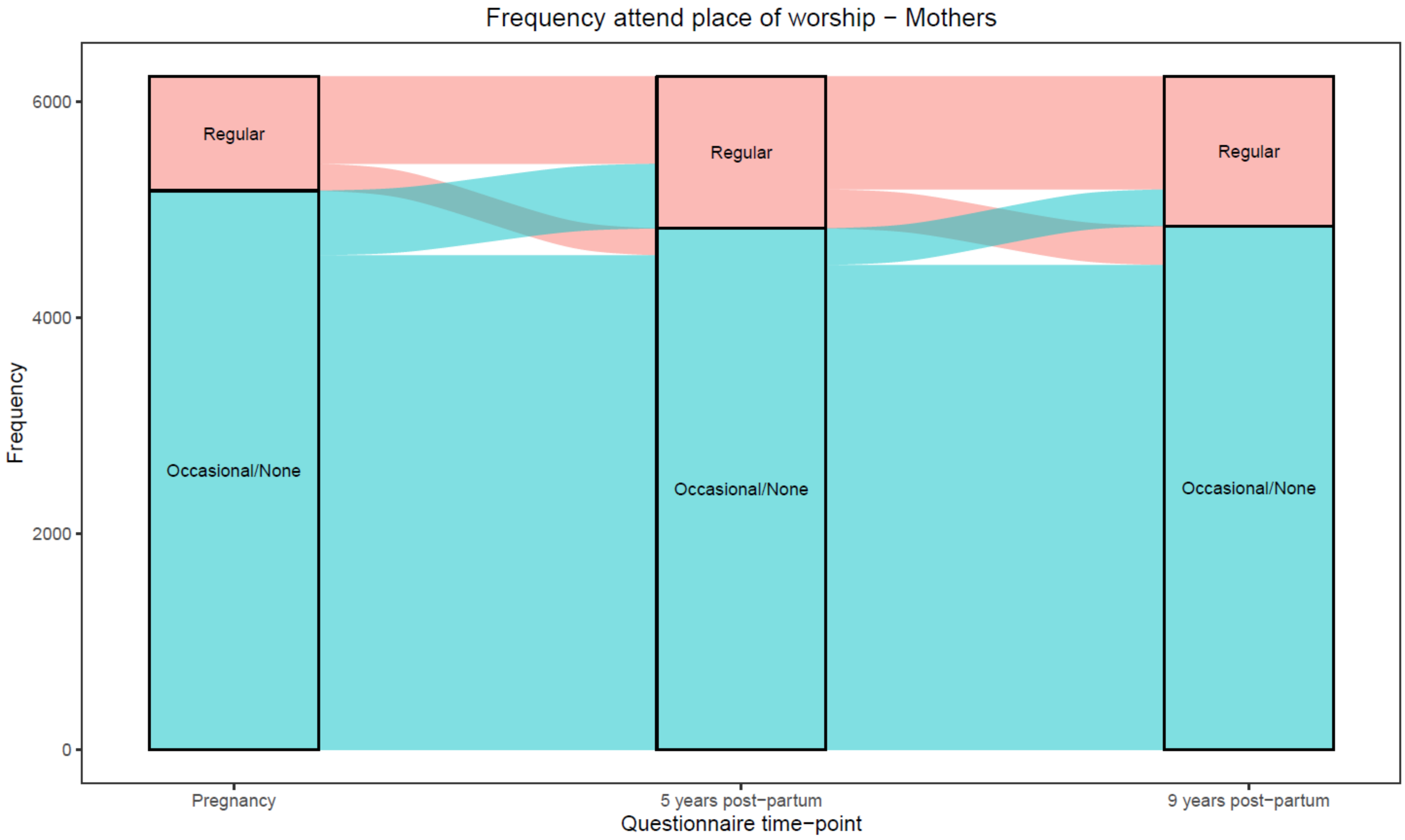

Figure S9: Change in religious attendance (frequency attend a place of worship) from pregnancy to 9 years post-partum for mothers (*n* = 6,233).

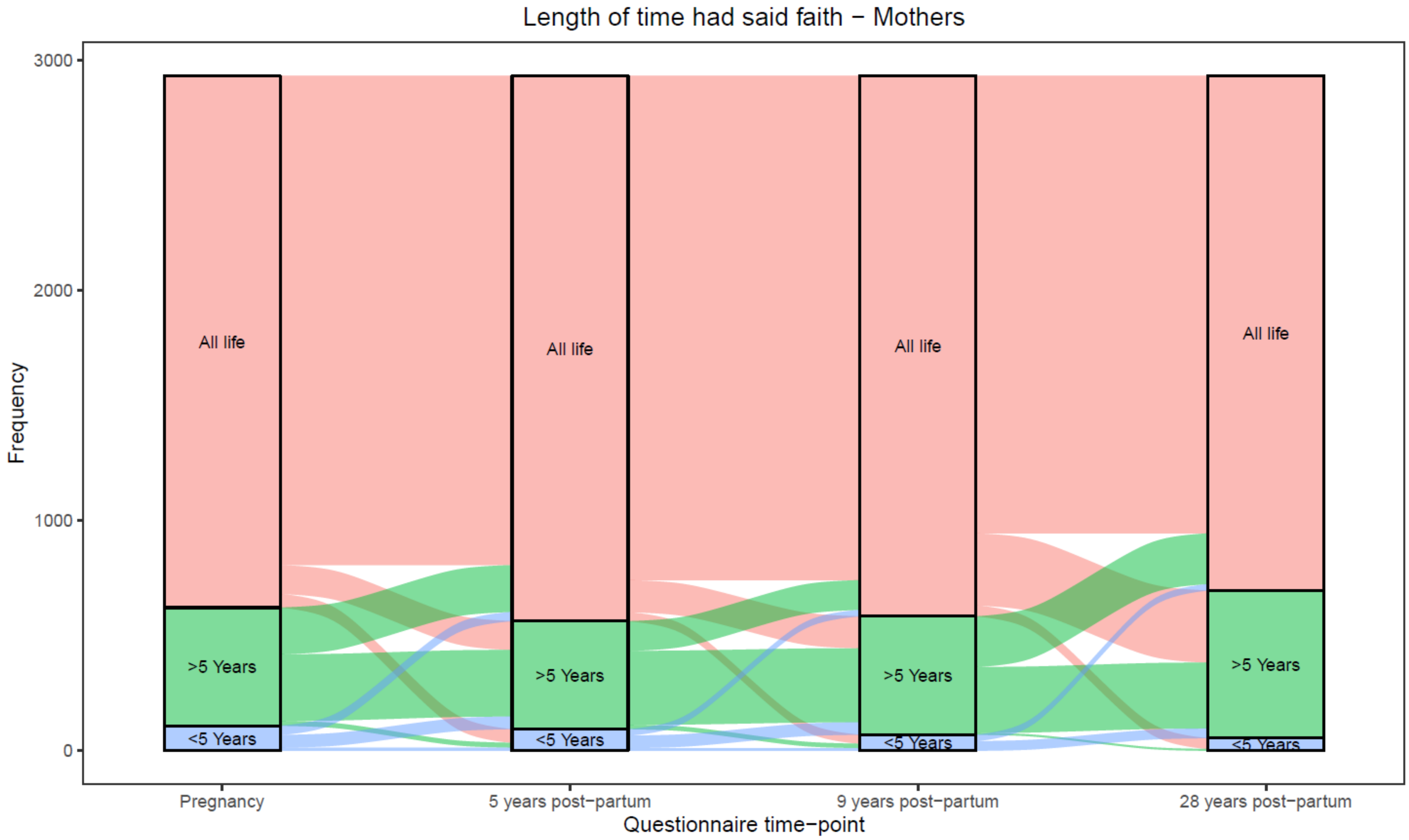

Figure S10: Change in length of time had current faith from pregnancy to 28 years post-partum for mothers (*n* = 2,931).

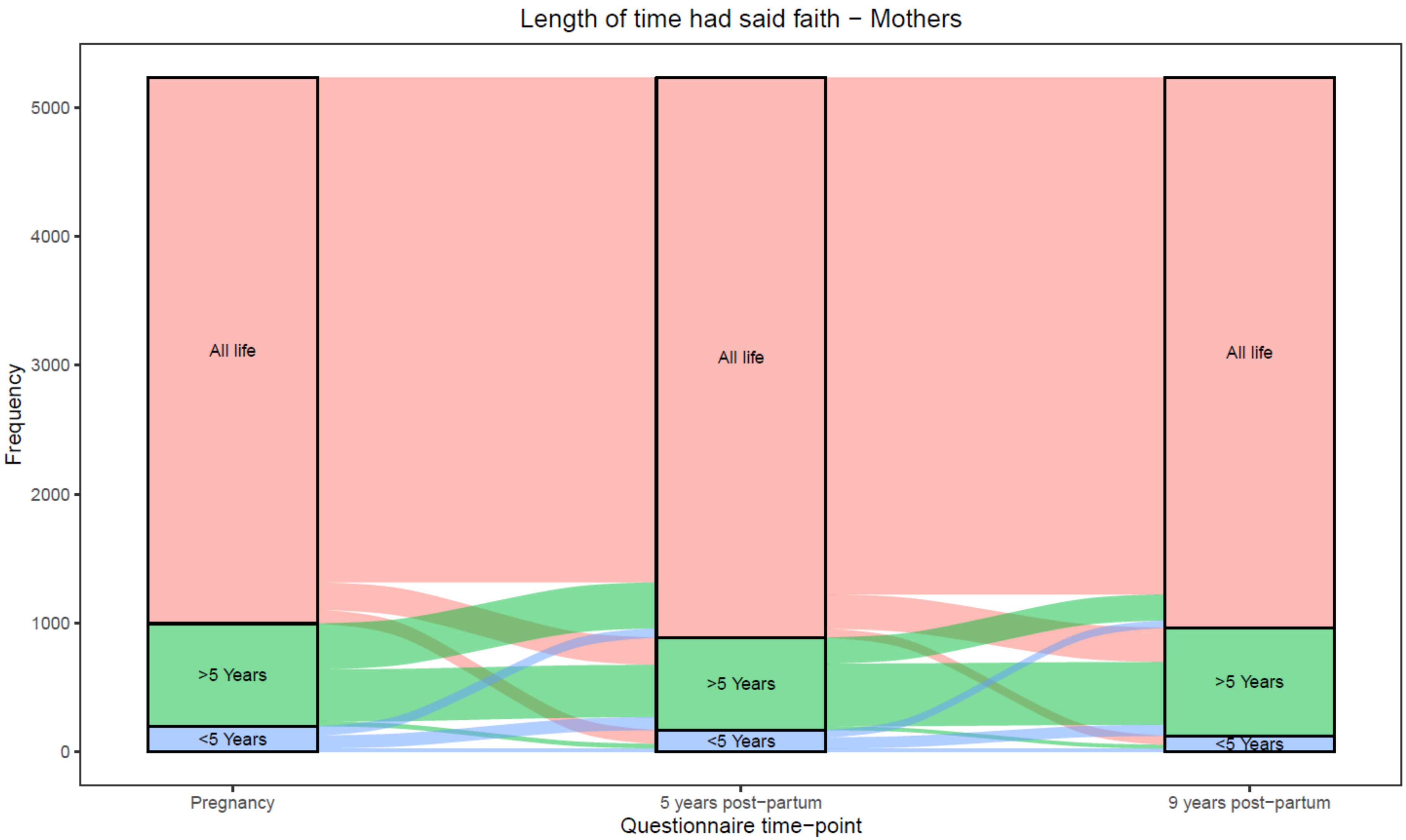

Figure S11: Change in length of time had current faith from pregnancy to 9 years post-partum for mothers (*n* = 5,232).

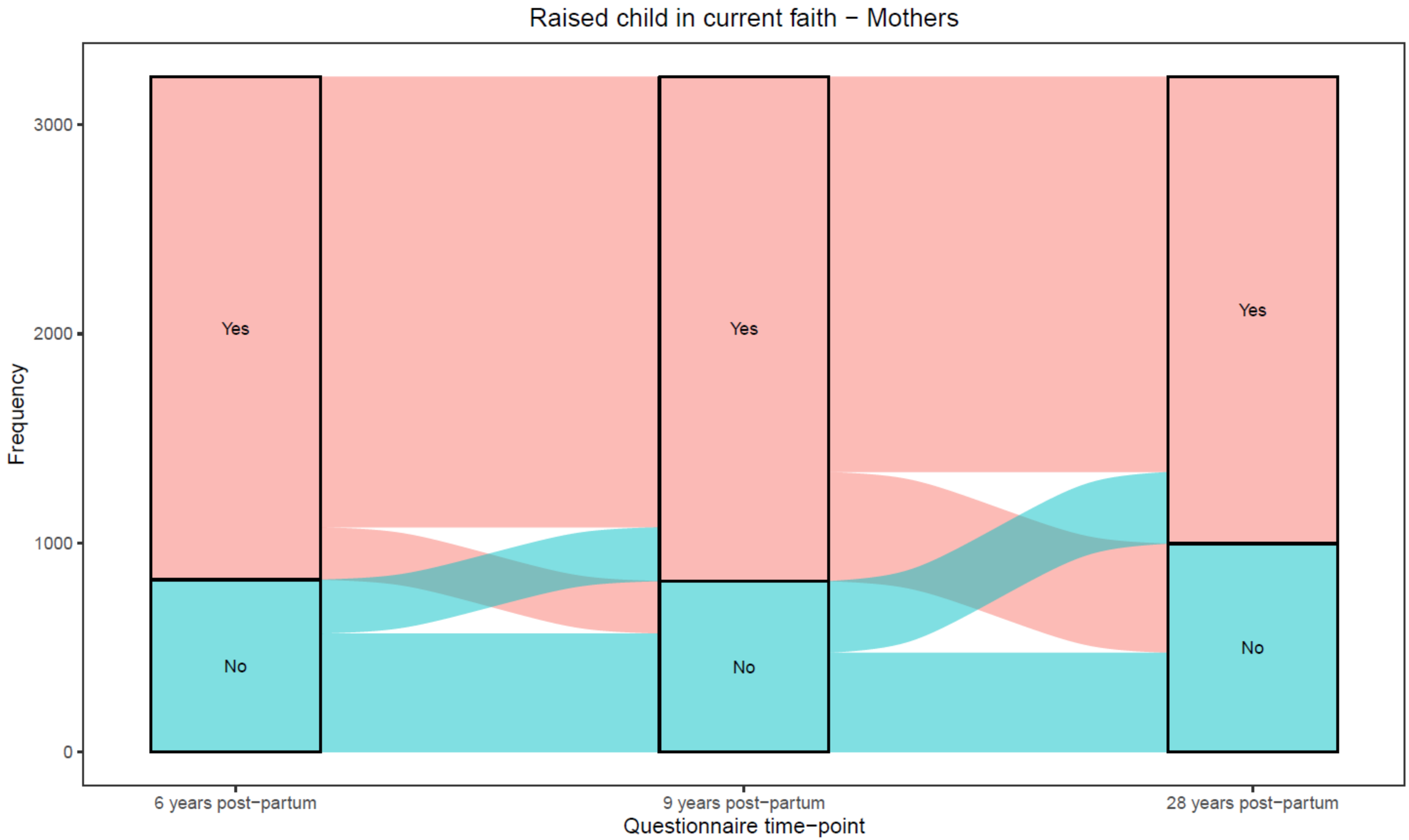

Figure S12: Change in whether bringing up study child in current faith from 6 to 28 years post-partum for mothers (*n* = 3,229; note that at 28 years post-partum this question was asked in the past tense "Did you bring up your child in your current faith/belief (including none)?").

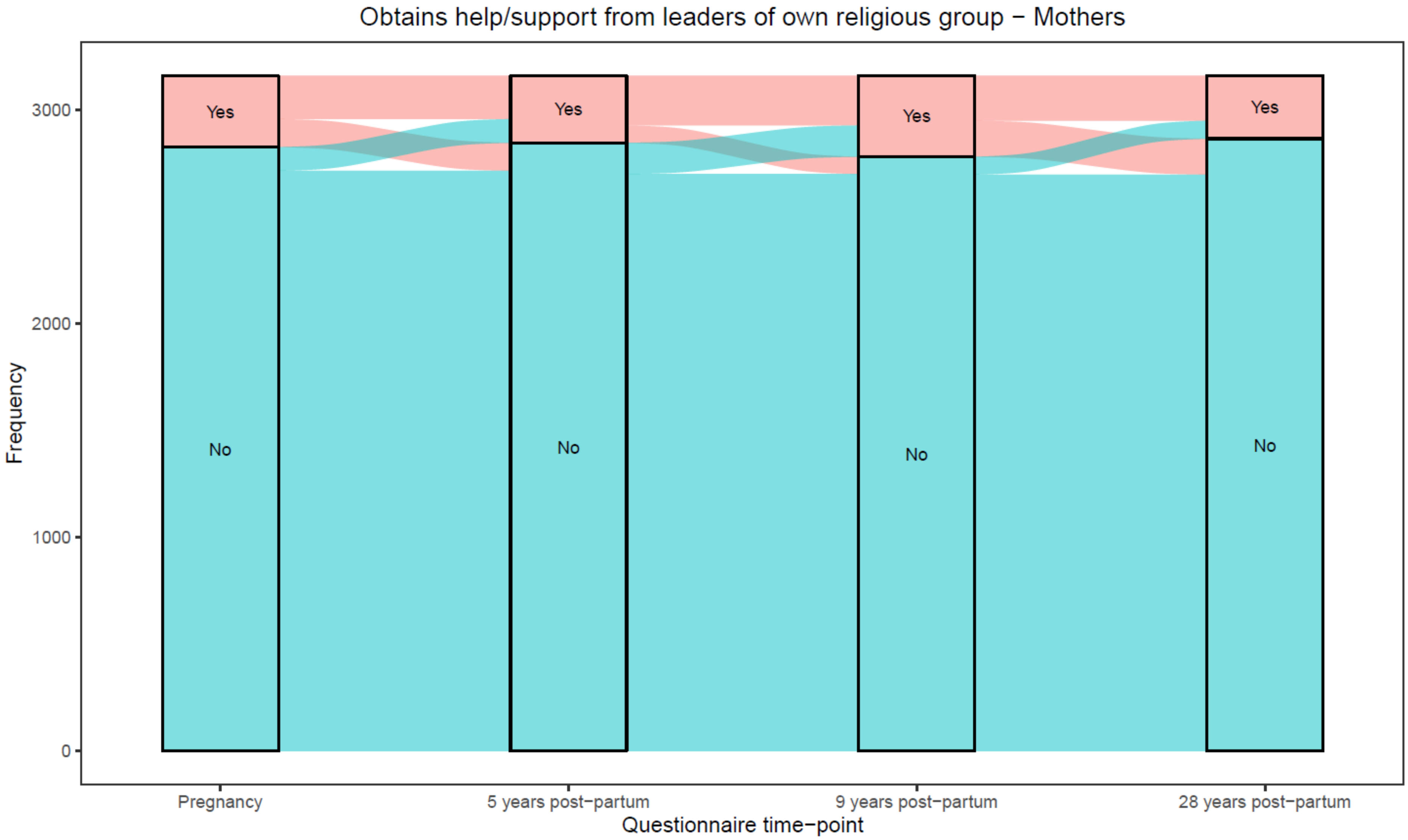

Figure S13: Change in whether obtain help/support from leaders of own religious group from pregnancy to 28 years post-partum for mothers (*n* = 3,160).

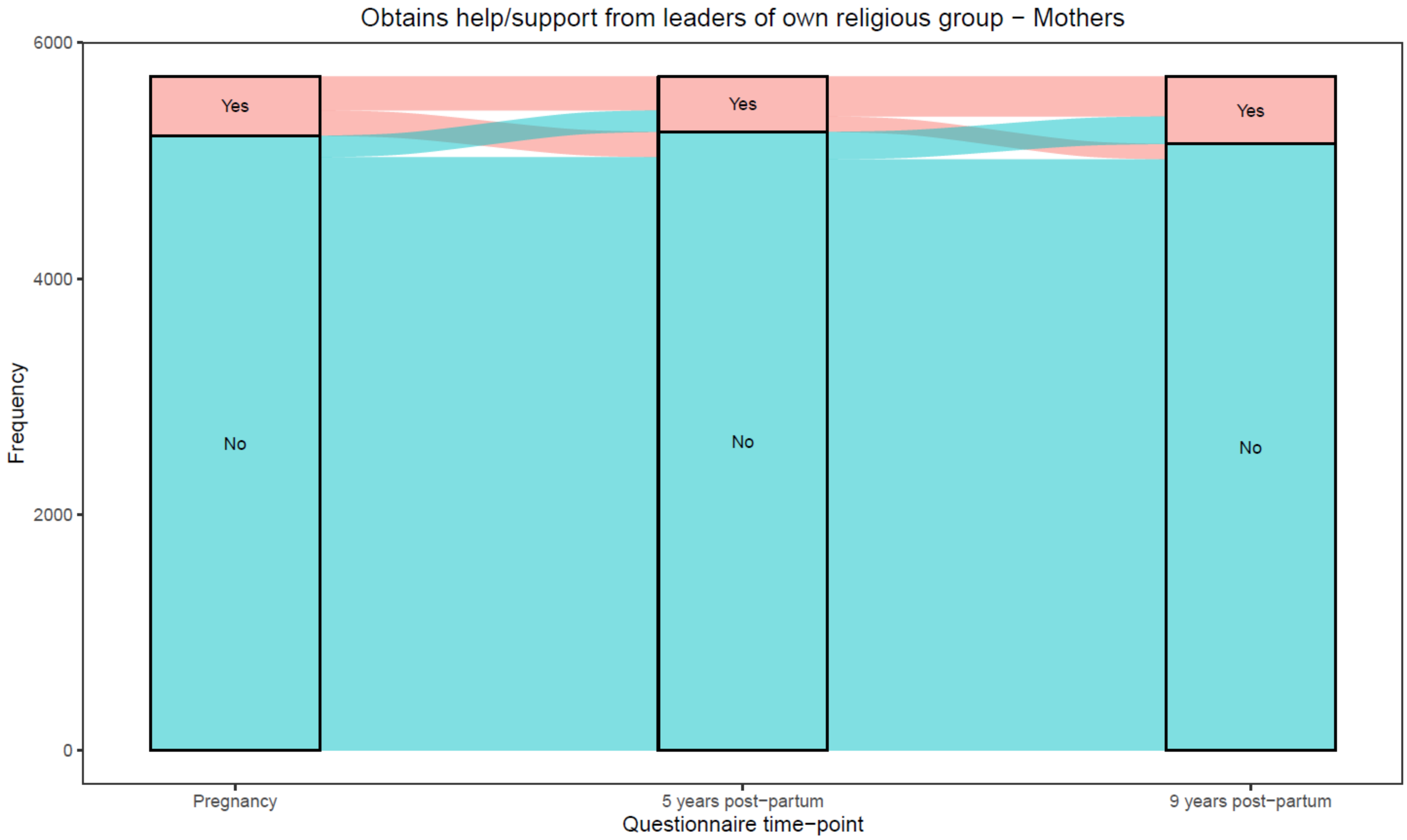

Figure S14: Change in whether obtain help/support from leaders of own religious group from pregnancy to 9 years post-partum for mothers (*n* = 5,715).

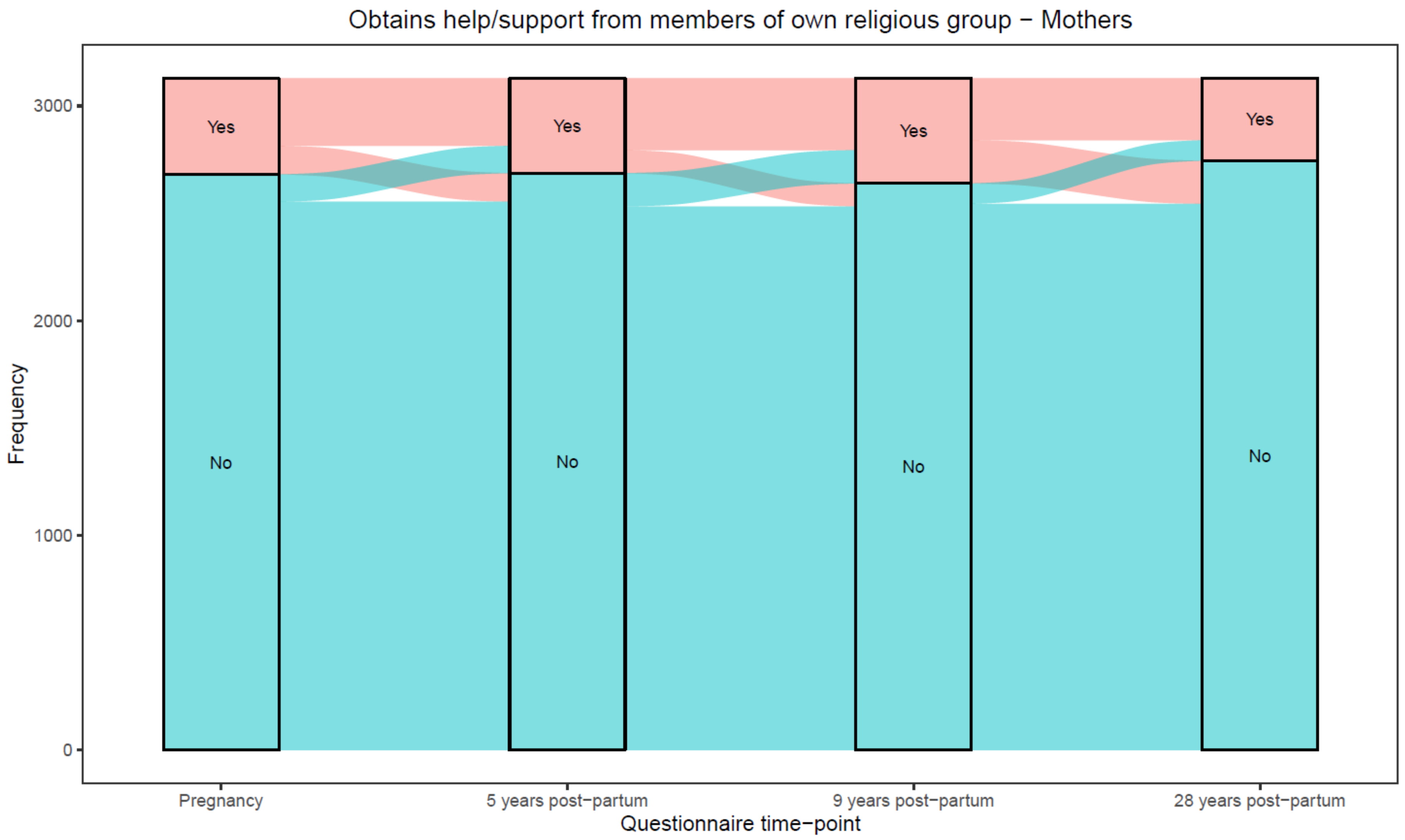

Figure S15: Change in whether obtain help/support from other members of own religious group from pregnancy to 28 years post-partum for mothers (*n* = 3,129).

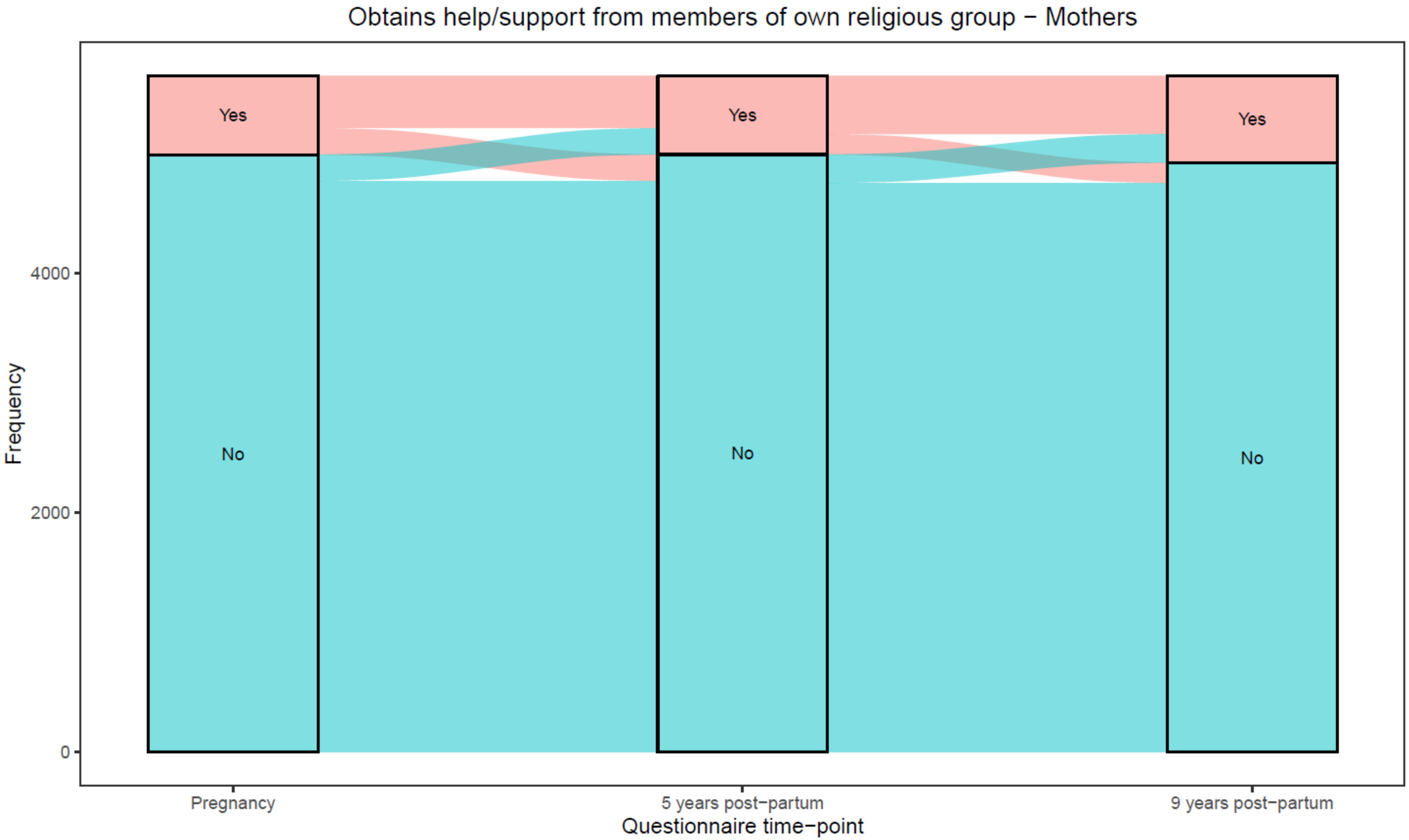

Figure S16: Change in whether obtain help/support from other members of own religious group from pregnancy to 9 years post-partum for mothers (*n* = 5,648).

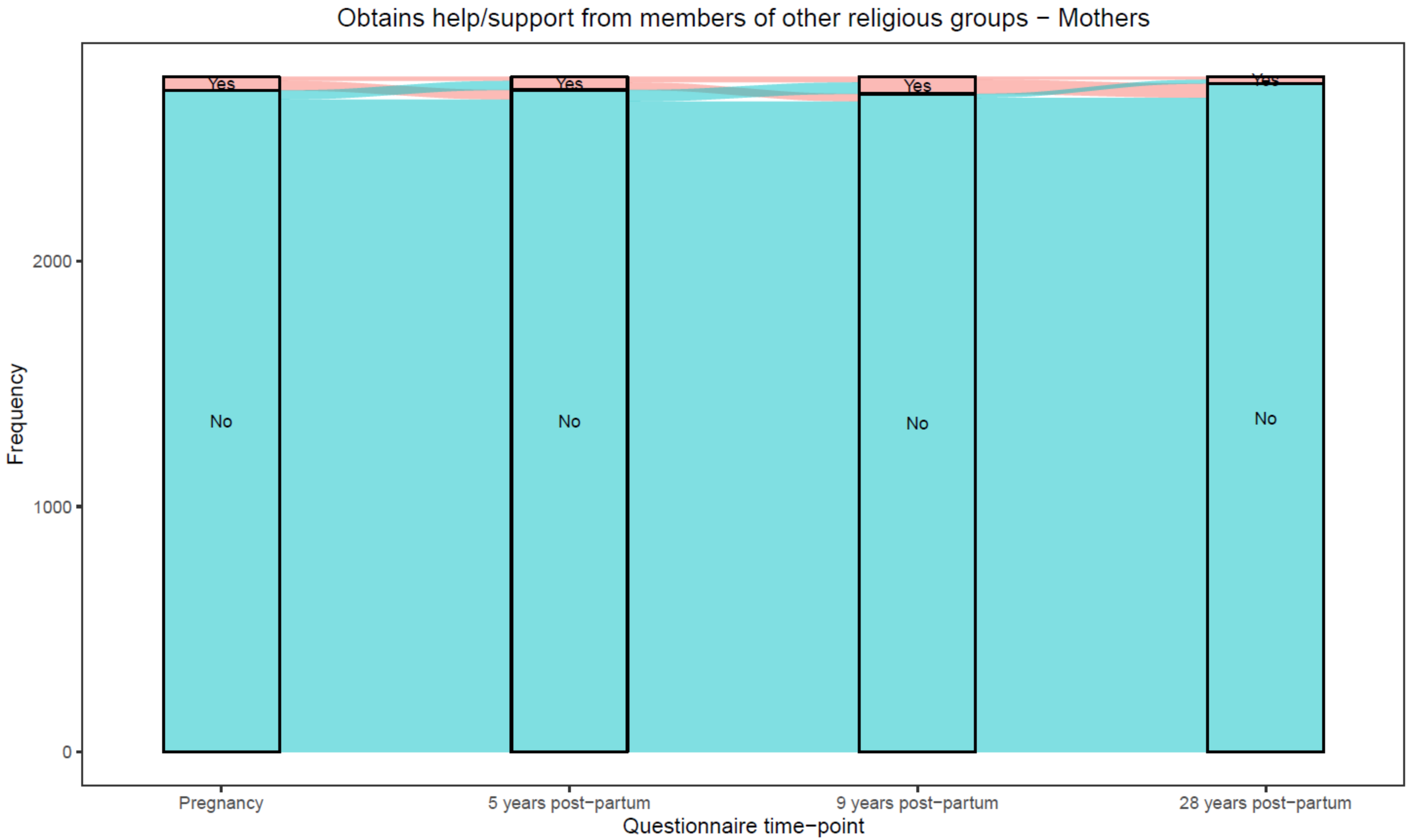

Figure S17: Change in whether obtain help/support from members of other religious groups from pregnancy to 28 years post-partum for mothers (n = 2,750).

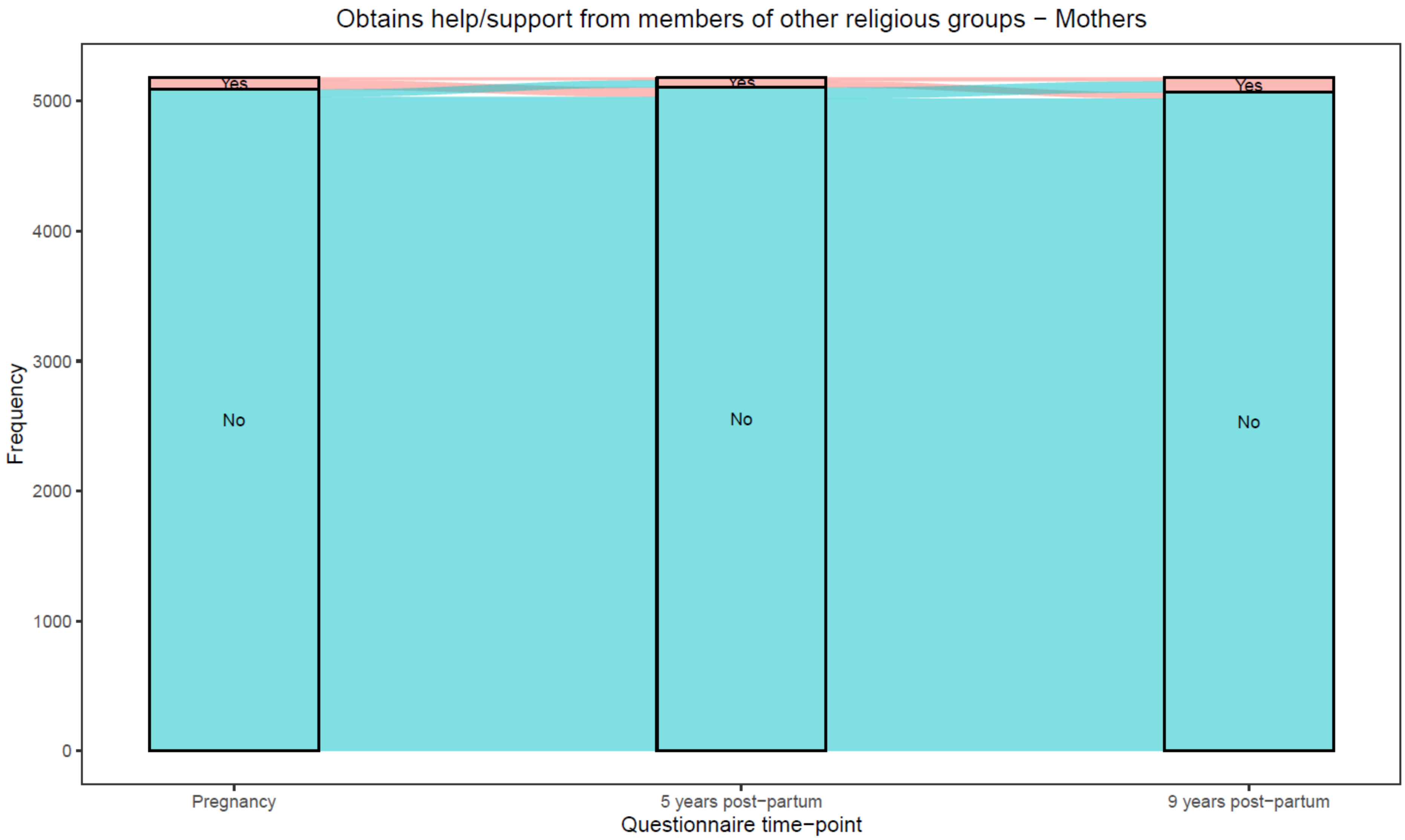

Figure S18: Change in whether obtain help/support from members of other religious groups from pregnancy to 9 years post-partum for mothers (*n* = 5,182).

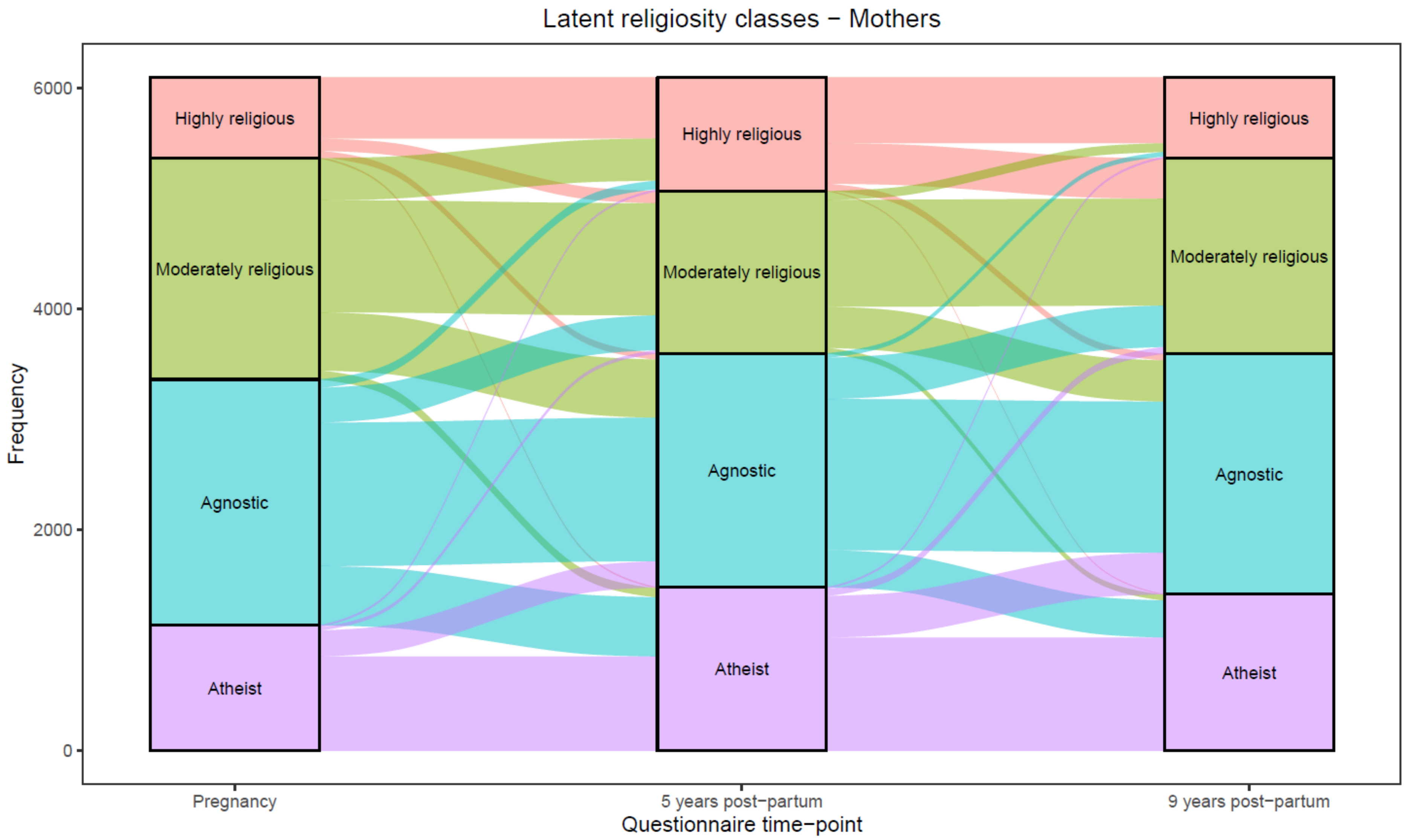

Figure S19: Change in the religiosity latent classes from pregnancy to 9 years post-partum for mothers (*n* = 6,096).

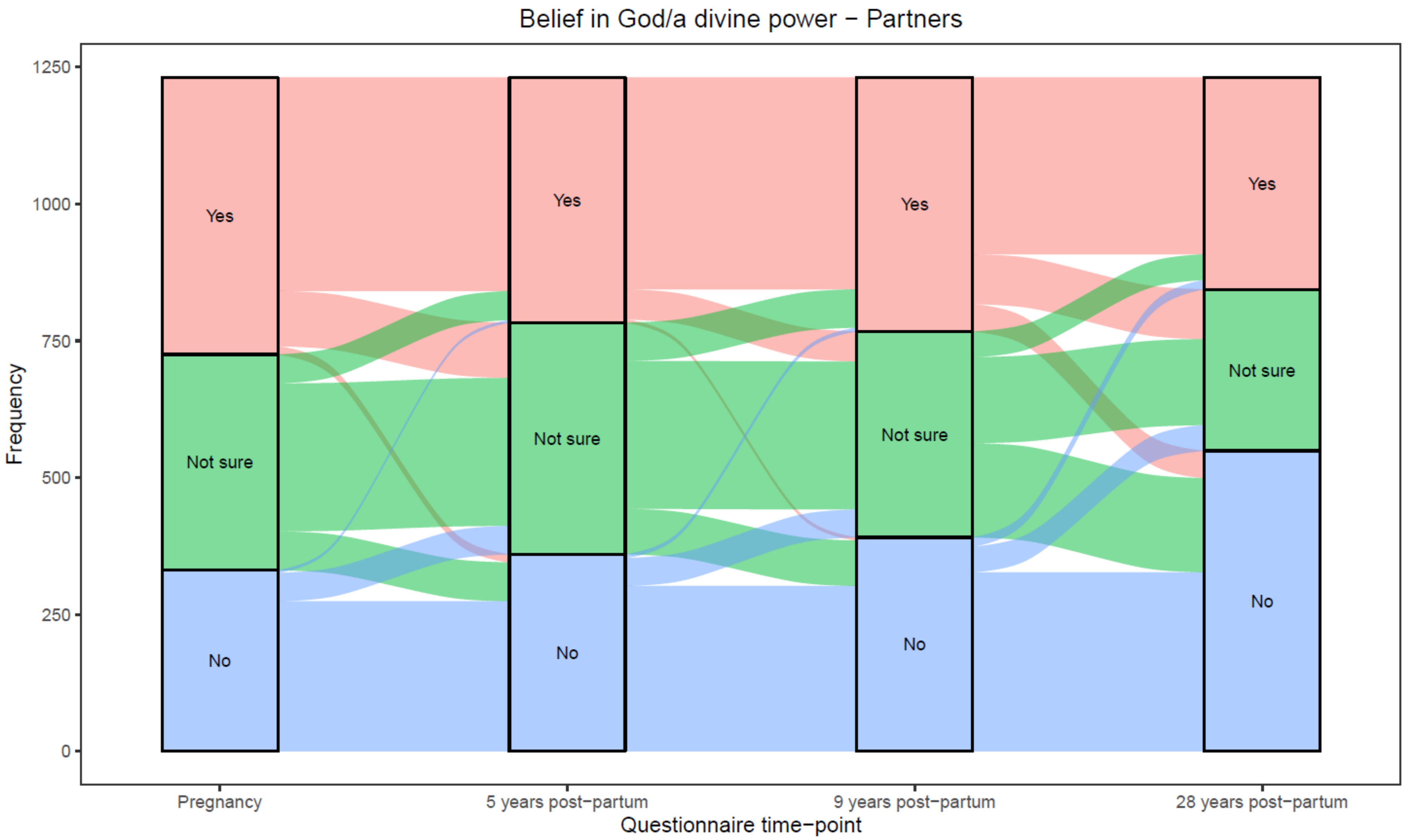

Figure S20: Change in belief in God/a divine power from pregnancy to 28 years post-partum for partners (*n* = 1,231).

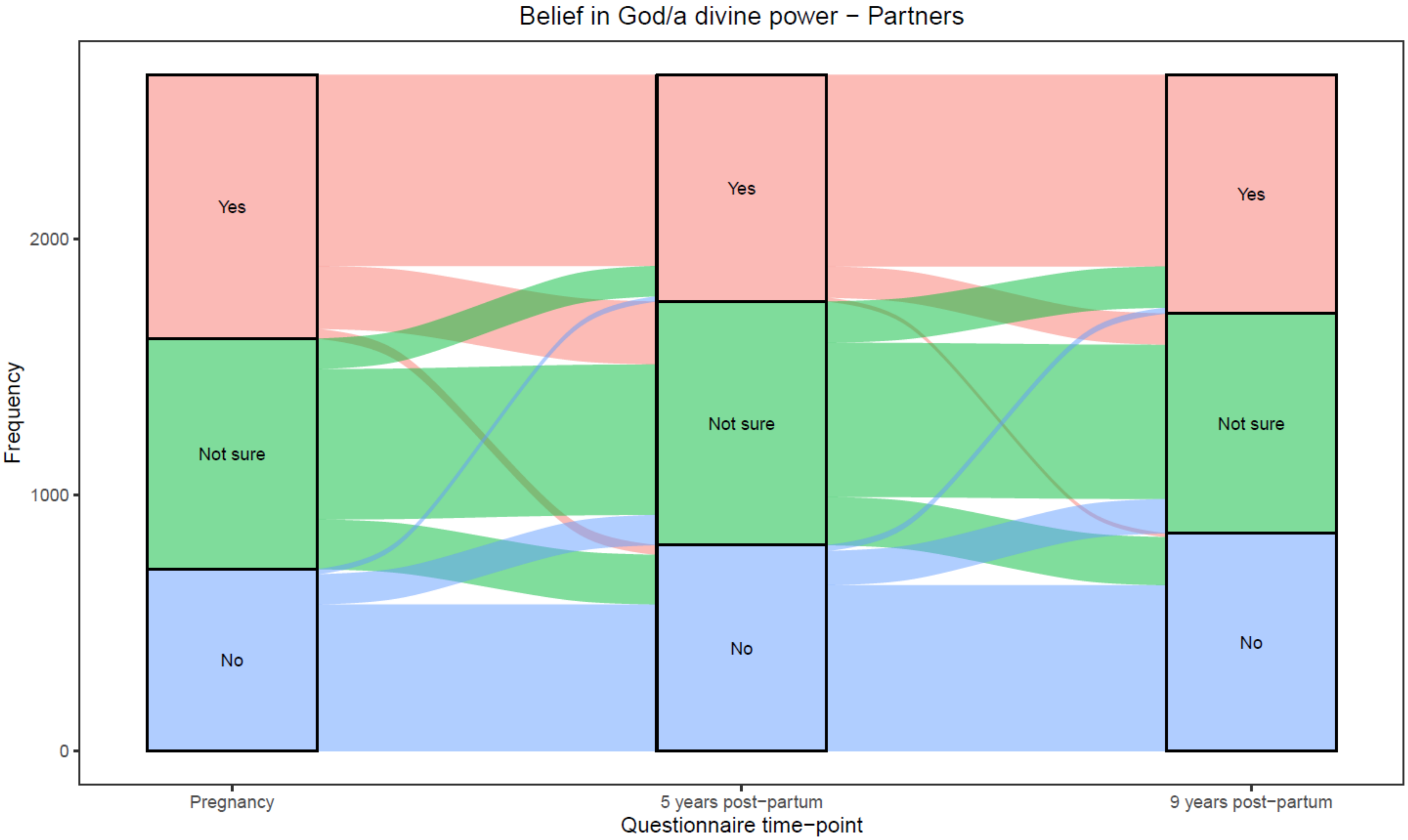

Figure S21: Change in belief in God/a divine power from pregnancy to 9 years post-partum for partners (*n* = 2,641).

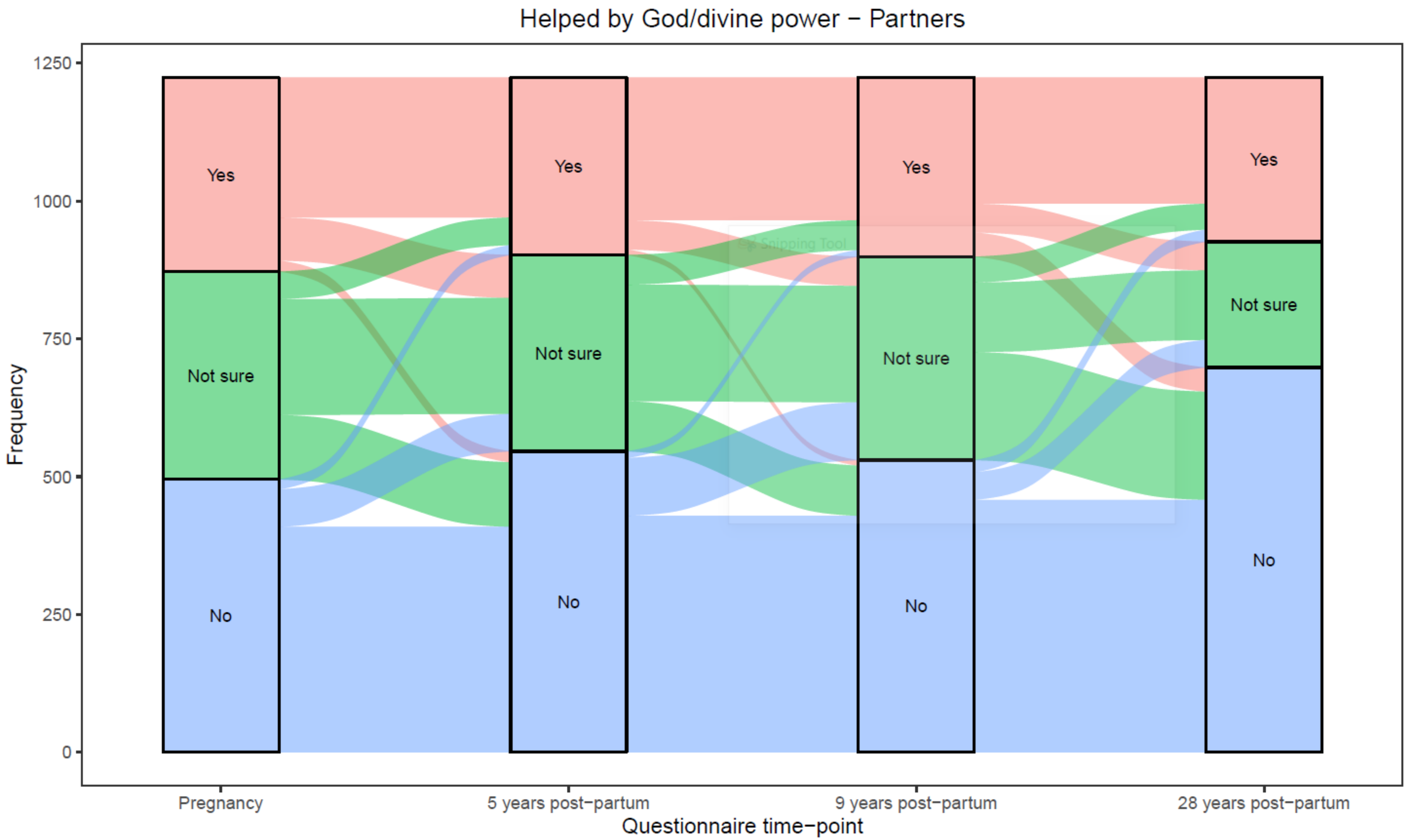

Figure S22: Change in 'ever helped by God/a divine power' from pregnancy to 28 years post-partum for partners (*n* = 1,224).

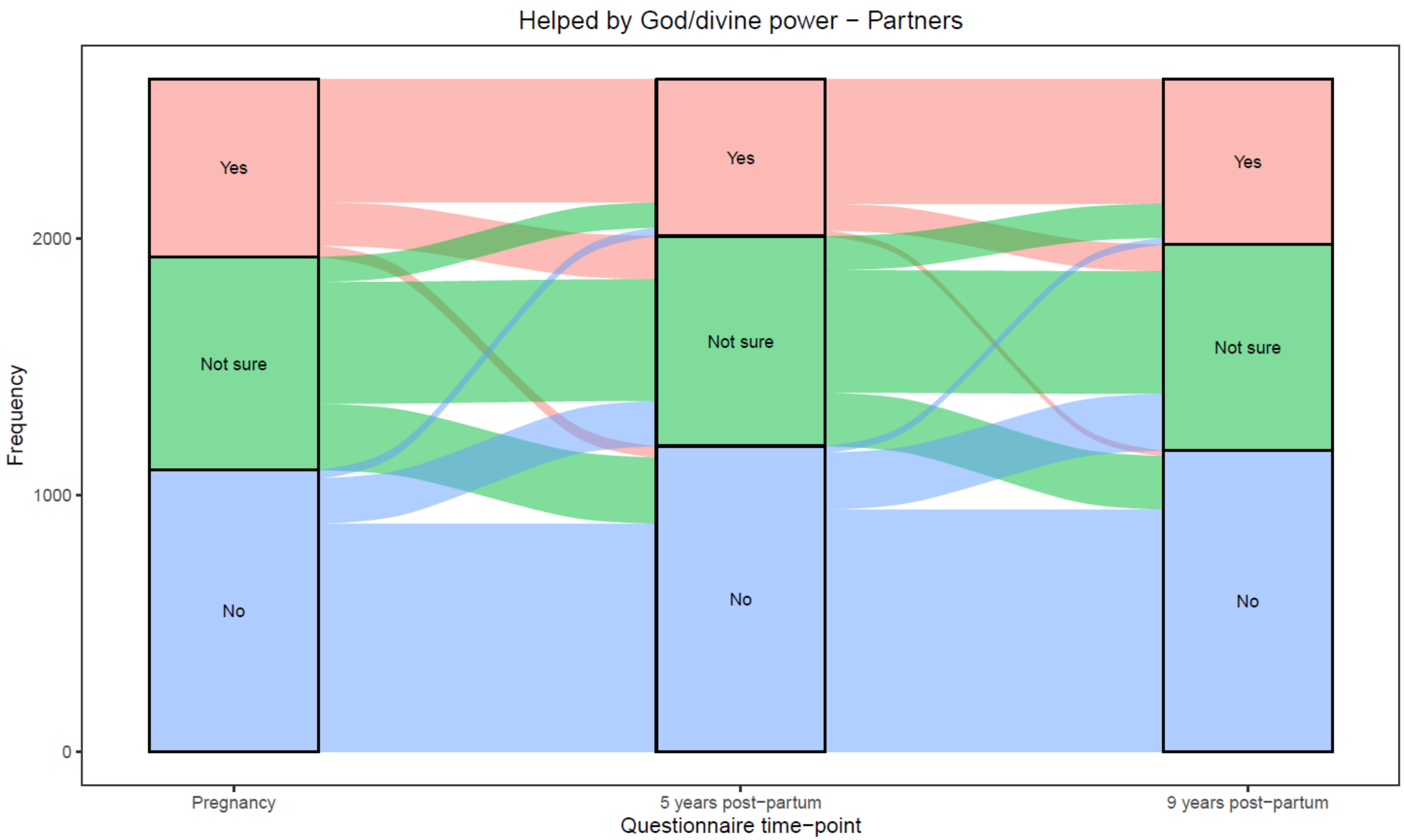

Figure S23: Change in 'ever helped by God/a divine power' from pregnancy to 9 years post-partum for partners (*n* = 2,622).

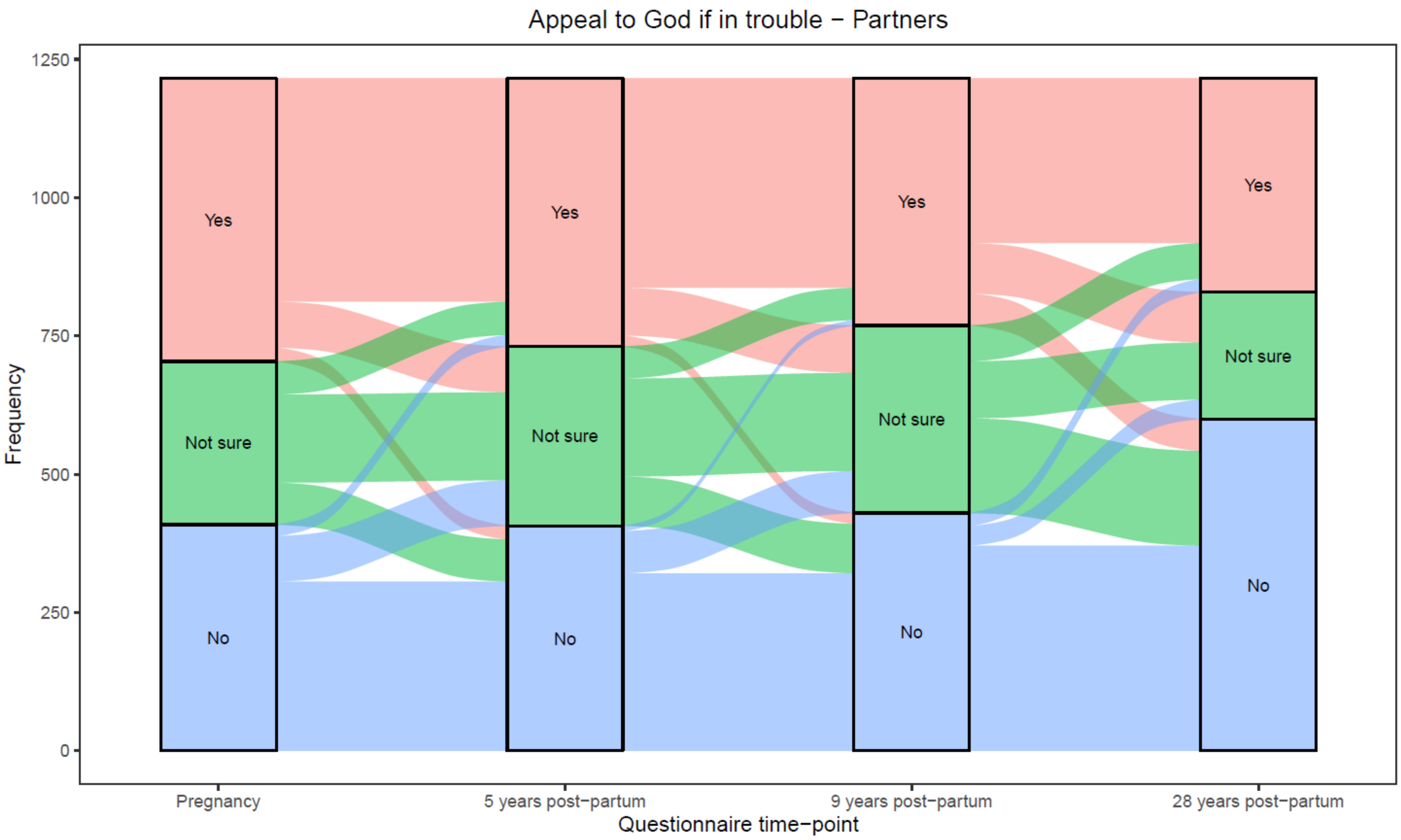

Figure S24: Change in 'would appeal to God for help if in trouble' from pregnancy to 28 years post-partum for partners (n = 1,216).

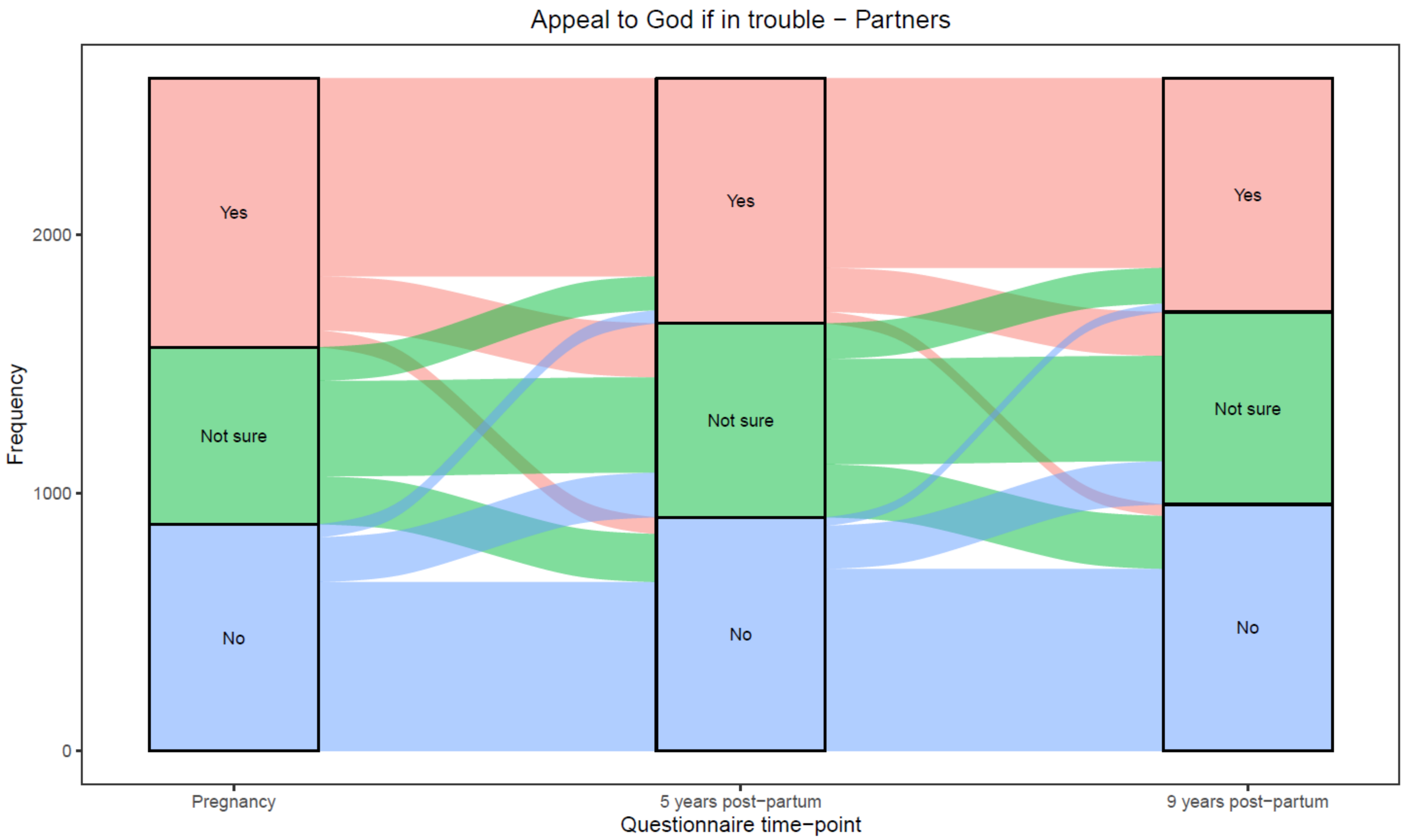

Figure S25: Change in 'would appeal to God for help if in trouble' from pregnancy to 9 years post-partum for partners (*n* = 2,607).

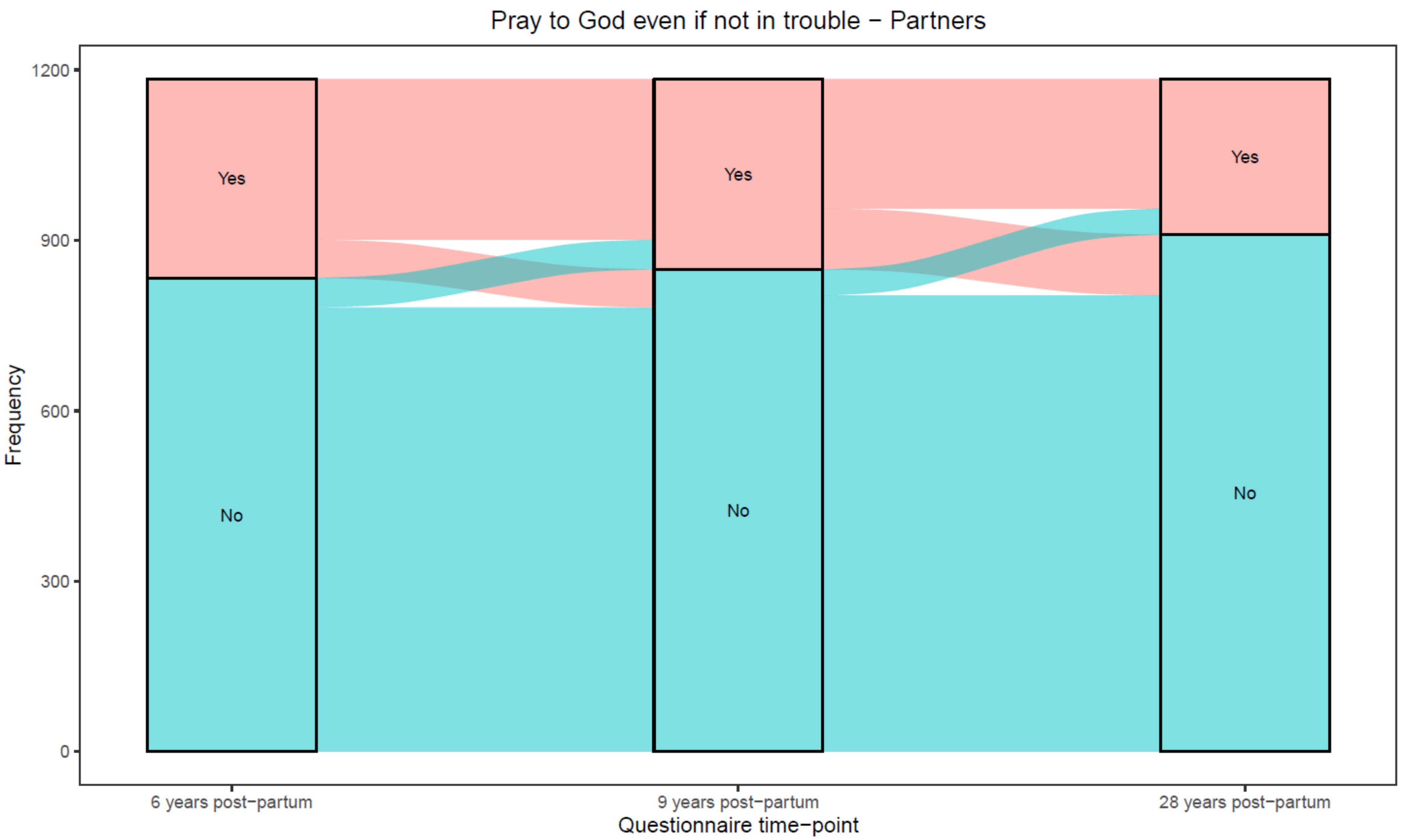

Figure S26: Change in 'would pray, even if not in trouble' from 6 to 28 years post-partum for partners (*n* = 1,184).

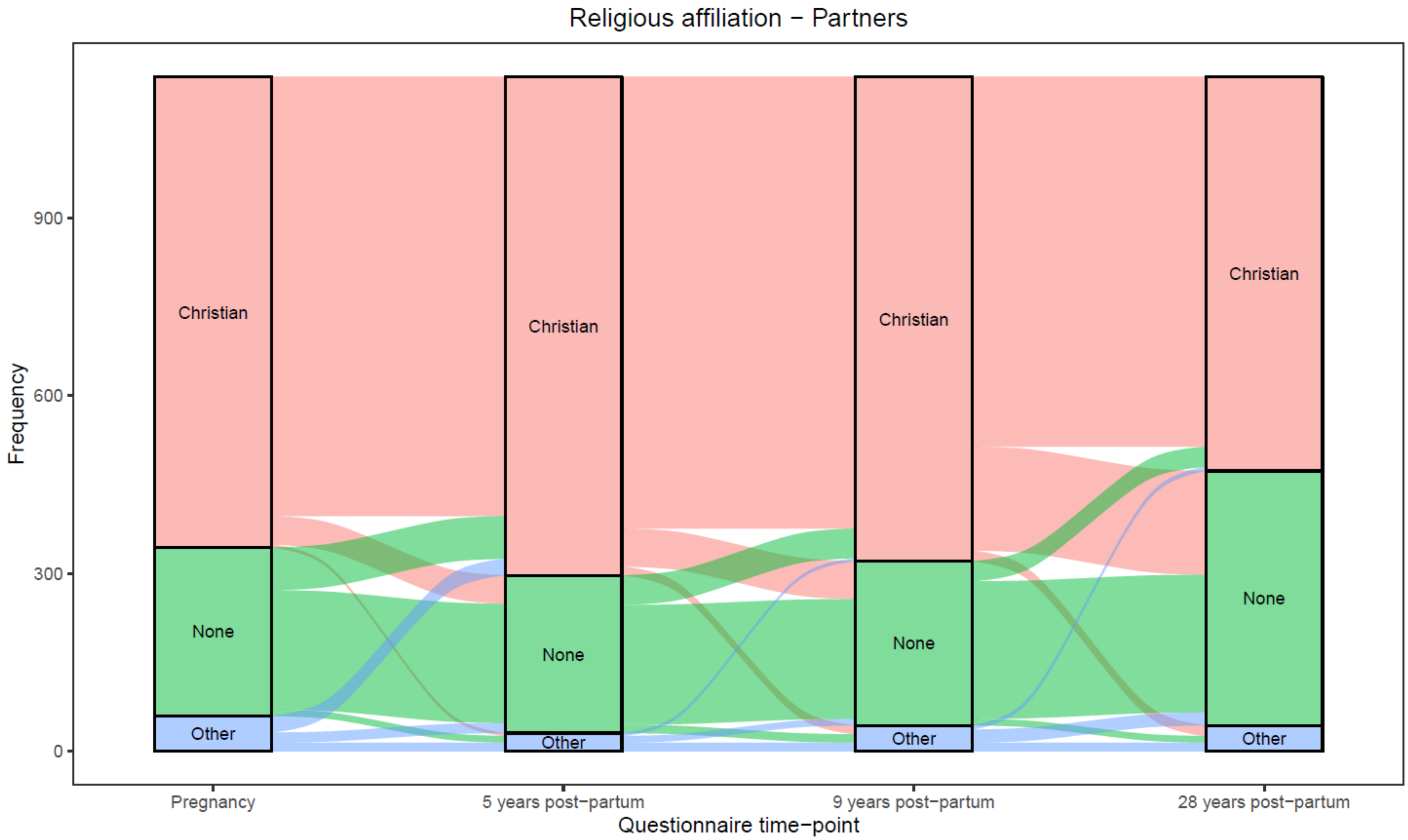

Figure S27: Change in religious affiliation (all Christians grouped together) from pregnancy to 28 years post-partum for partners (n = 1,138).

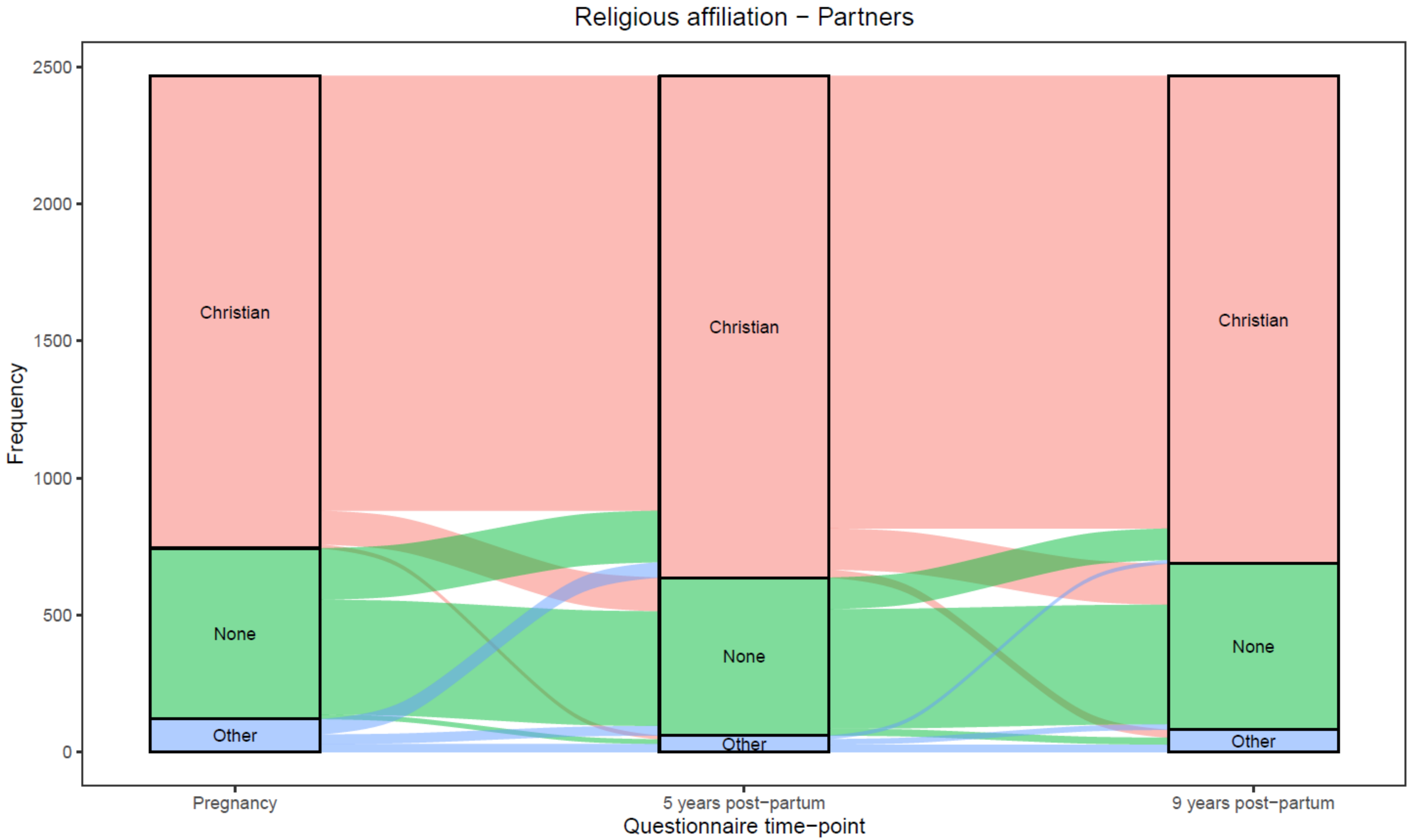

Figure S28: Change in religious affiliation (all Christians grouped together) from pregnancy to 9 years post-partum for partners (n = 2,467).

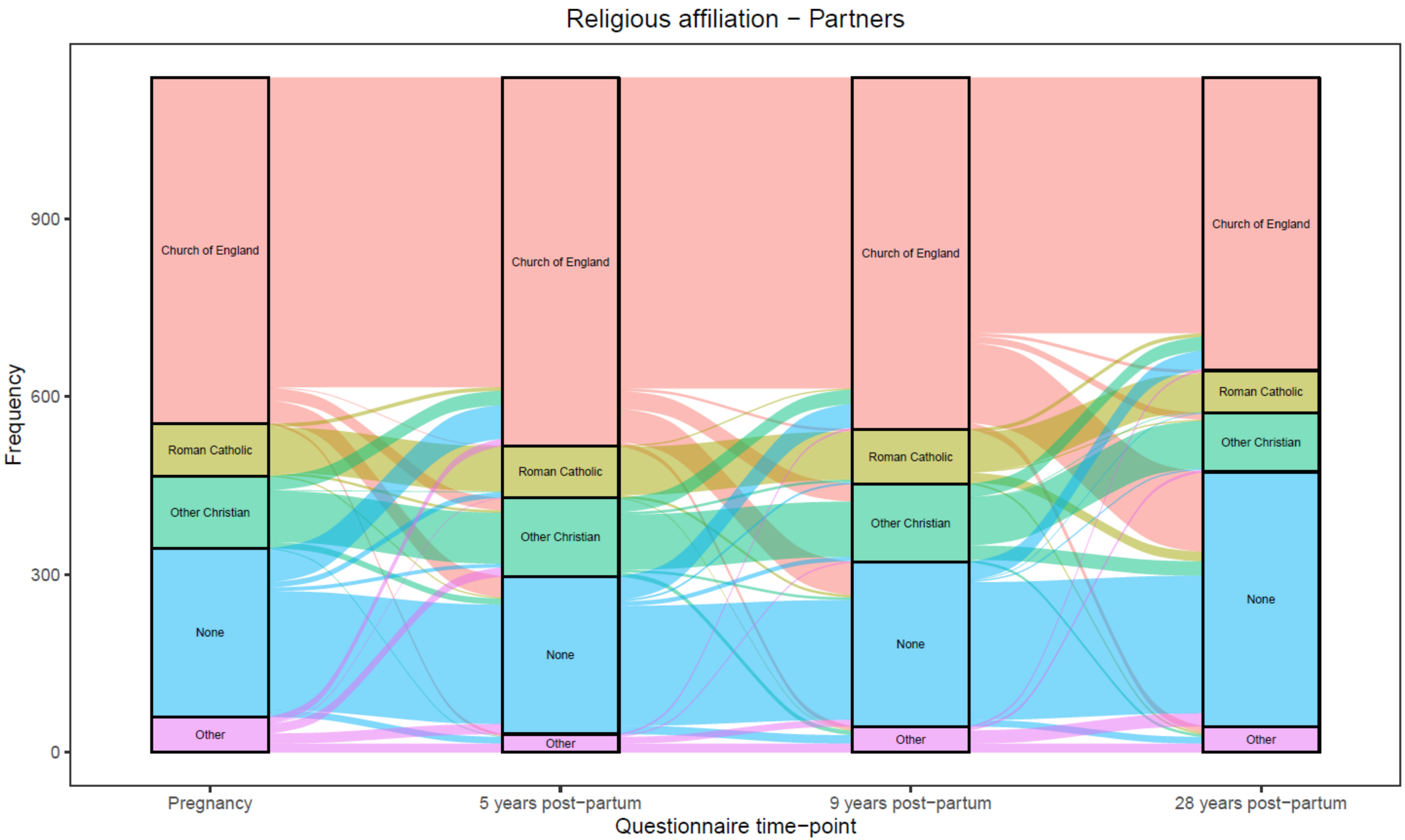

Figure S29: Change in religious affiliation (Christians split into 'Church of England', 'Roman Catholic' and 'Other Christian') from pregnancy to 28 years post-partum for partners (*n* = 1,138).

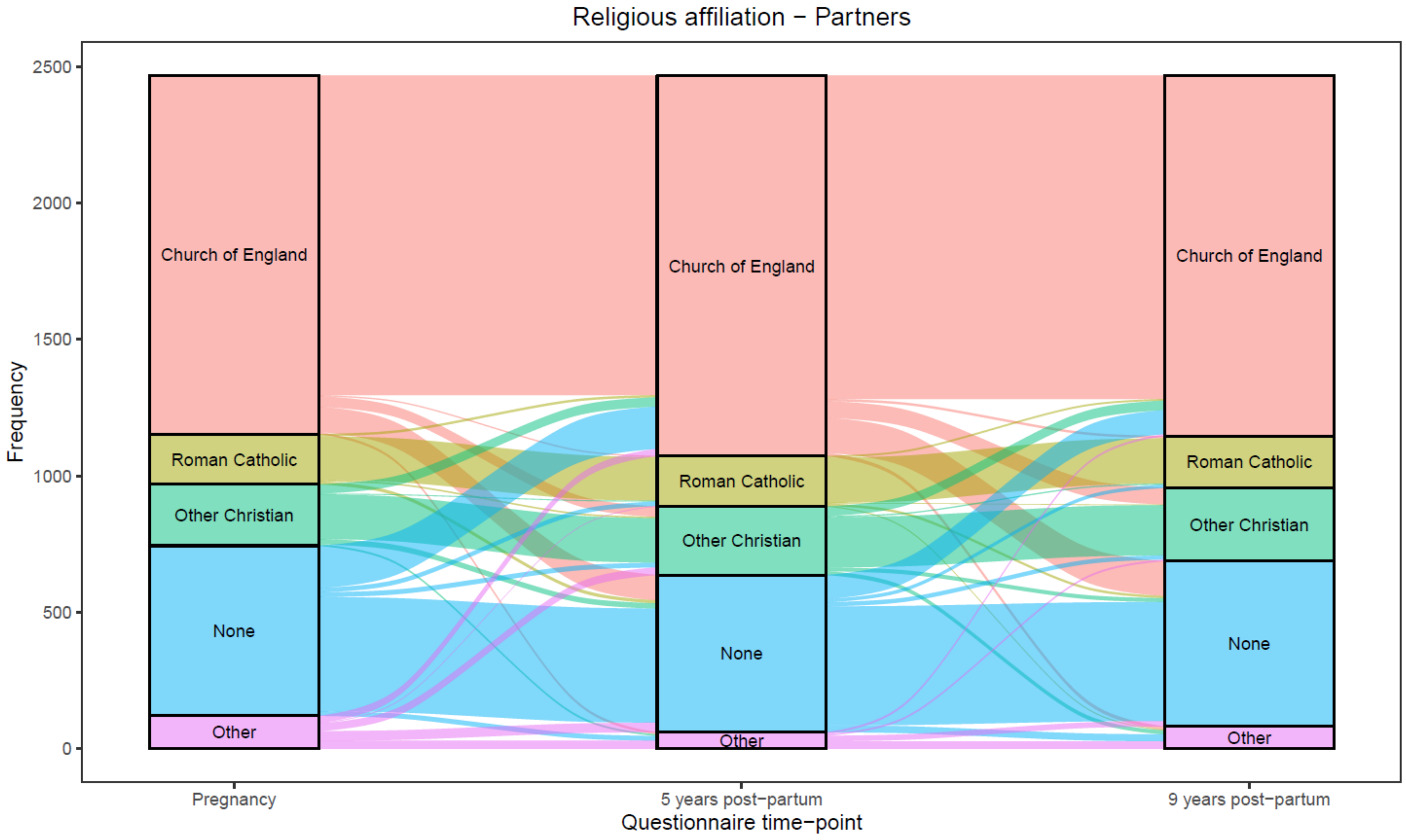

Figure S30: Change in religious affiliation (Christians split into ‘Church of England’, ‘Roman Catholic’ and ‘Other Christian’) from pregnancy to 9 years post-partum for partners (*n* = 2,467).

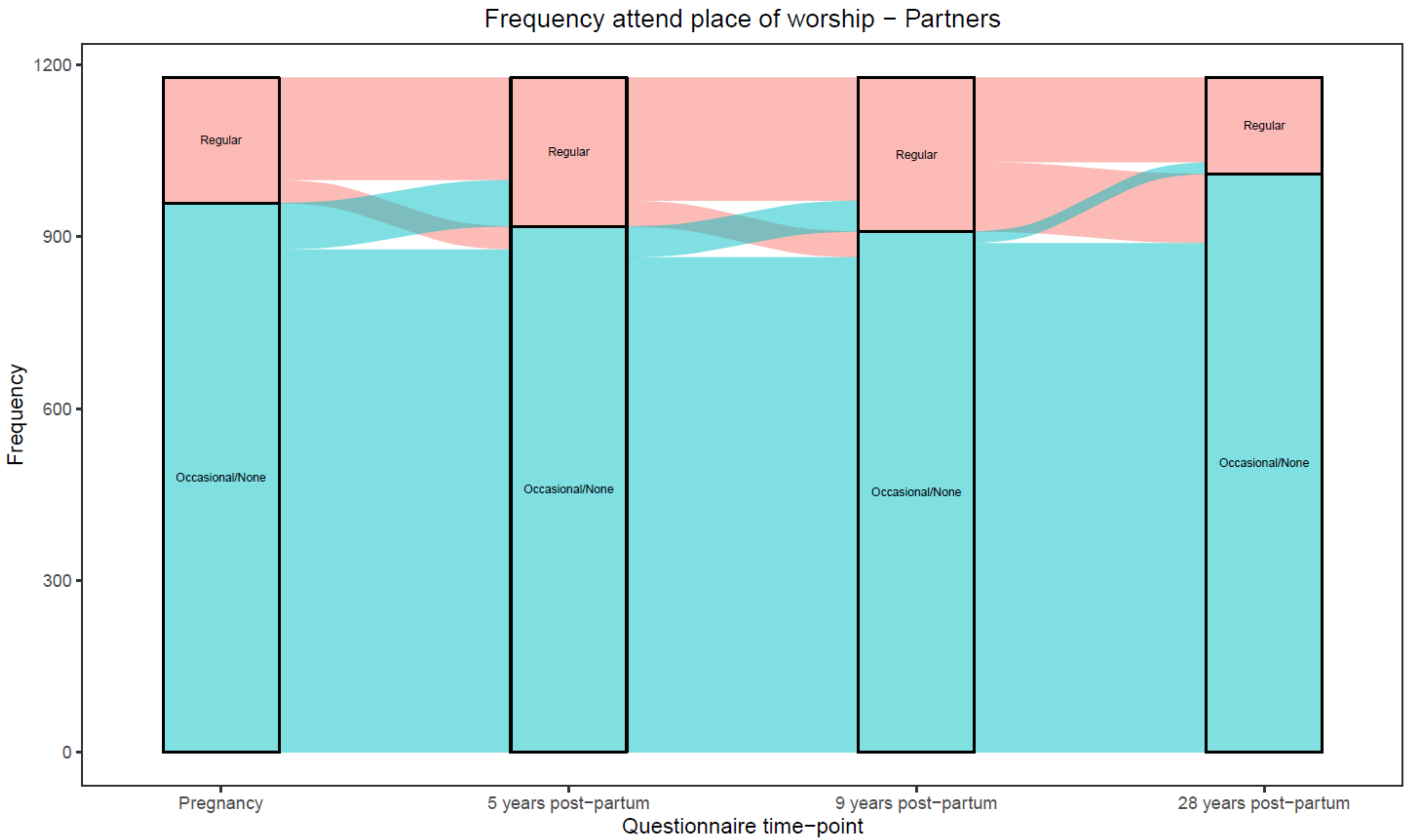

Figure S31: Change in religious attendance (frequency attend a place of worship) from pregnancy to 28 years post-partum for partners (*n* = 1,178).

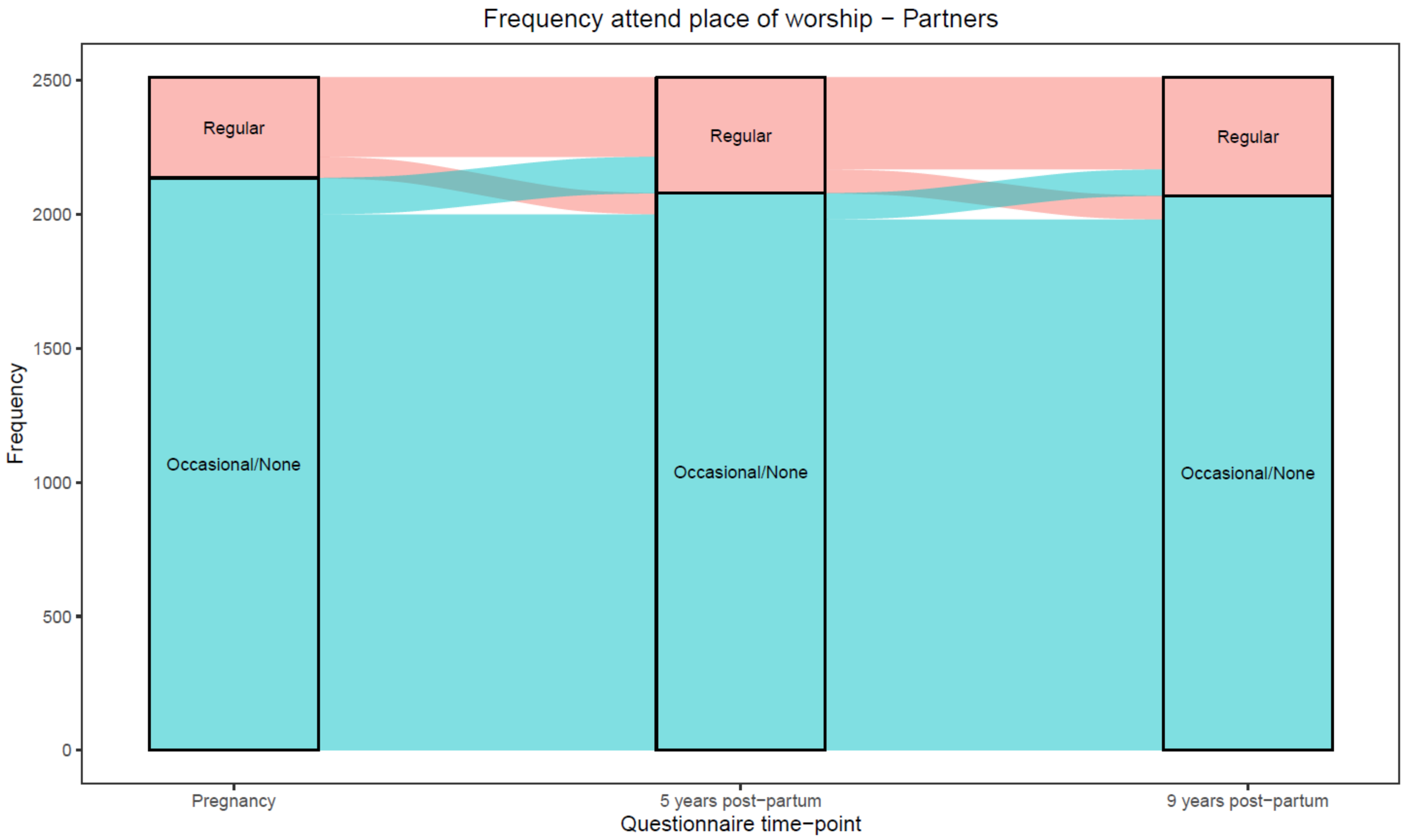

Figure S32: Change in religious attendance (frequency attend a place of worship) from pregnancy to 9 years post-partum for partners (*n* = 2,512).

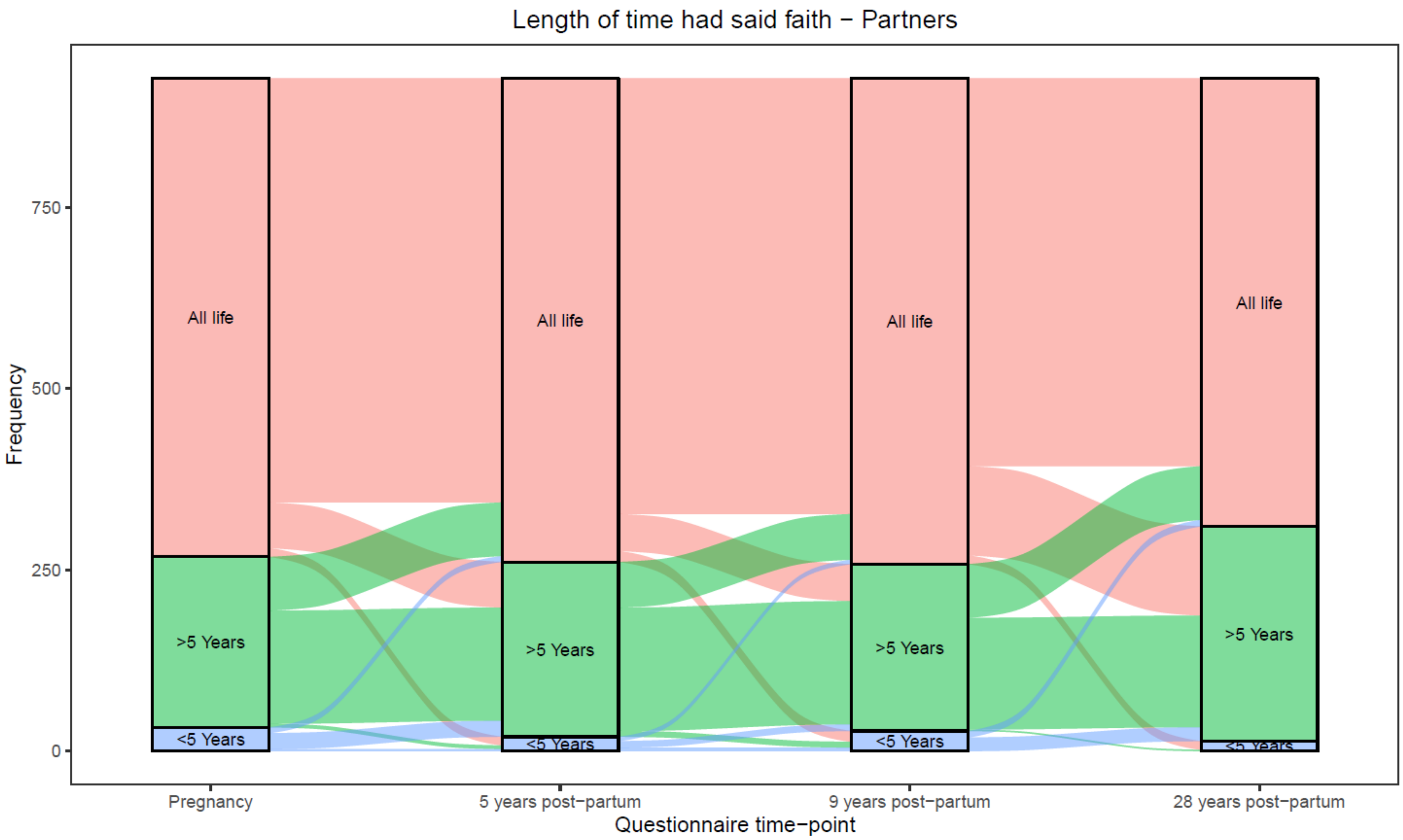

Figure S33: Change in length of time had current faith from pregnancy to 28 years post-partum for partners (n = 928).

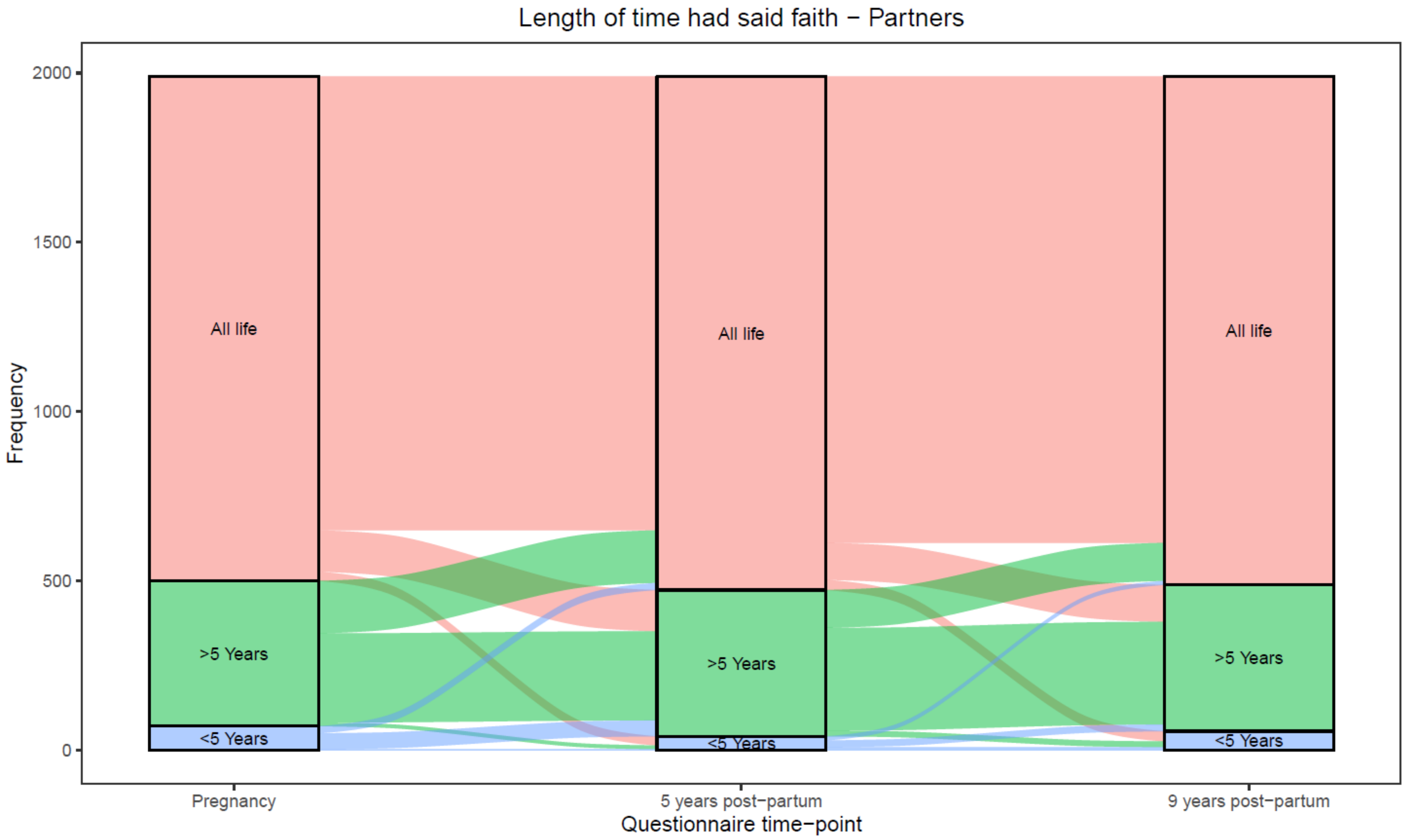

Figure S34: Change in length of time had current faith from pregnancy to 9 years post-partum for partners (*n* = 1,990).

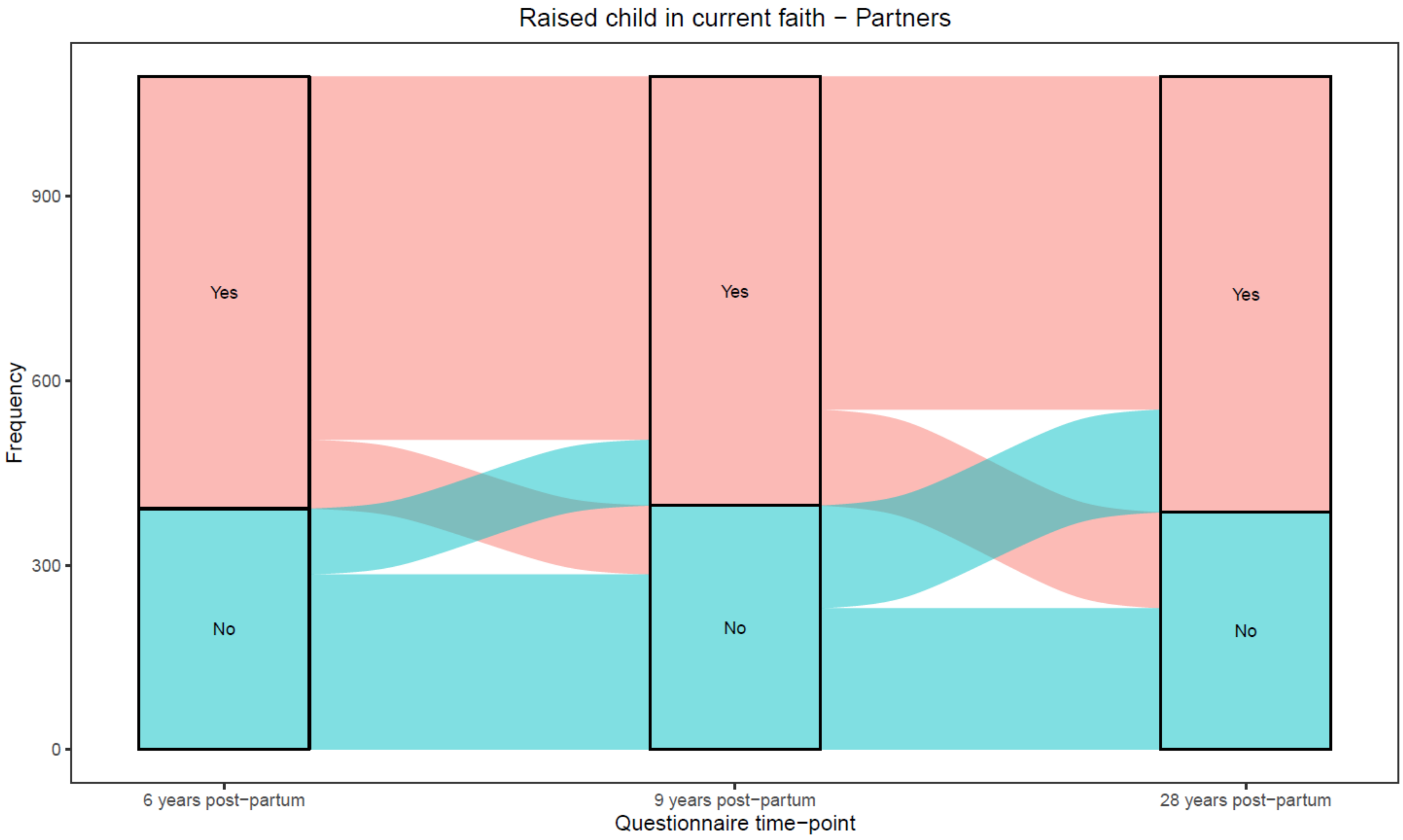

Figure S35: Change in whether bringing up study child in current faith from 6 to 28 years post-partum for partners (*n* = 1,095; note that at 28 years post-partum this question was asked in the past tense “Did you bring up your child in your current faith/belief (including none)?”).

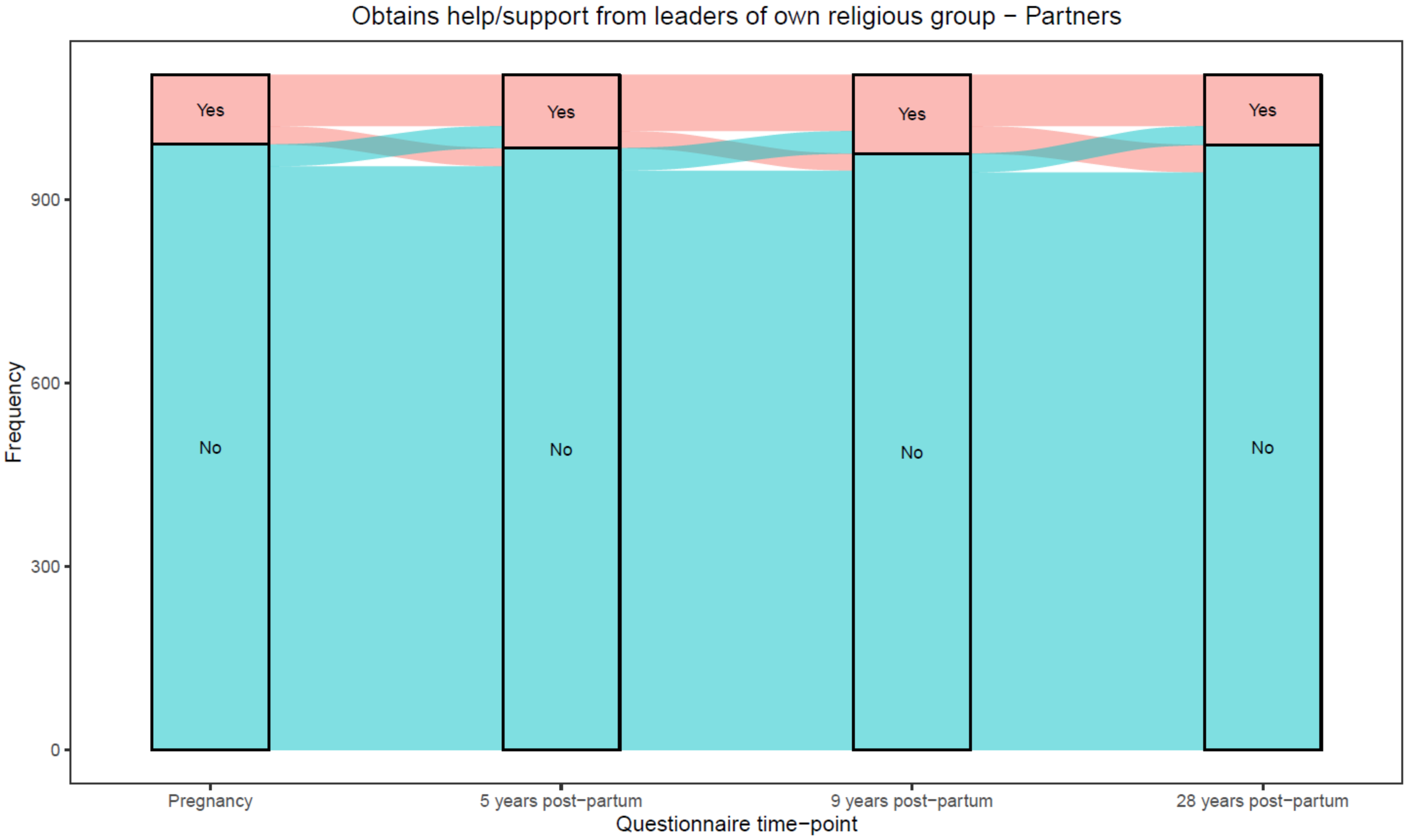

Figure S36: Change in whether obtain help/support from leaders of own religious group from pregnancy to 28 years post-partum for partners (*n* = 1,105).

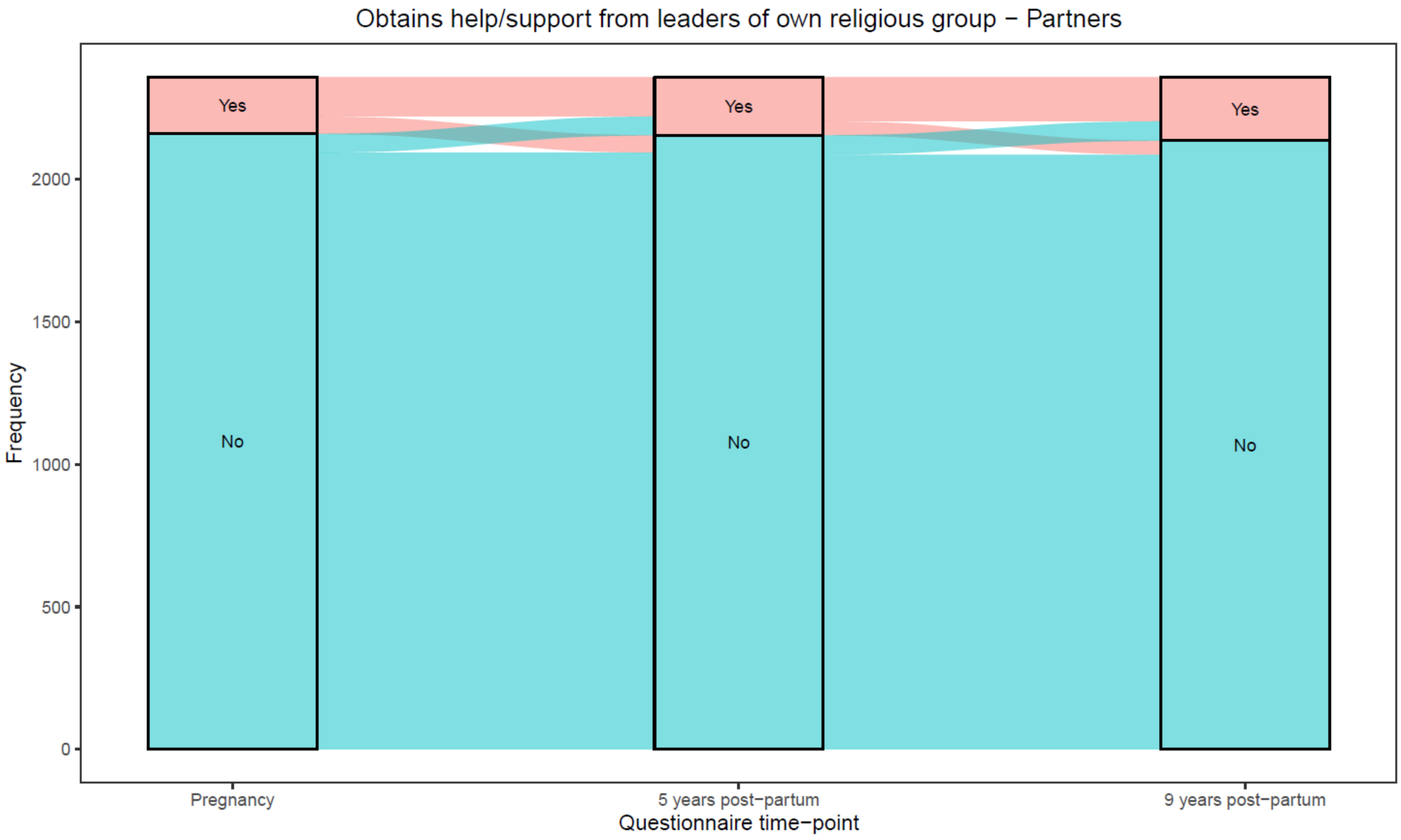

Figure S37: Change in whether obtain help/support from leaders of own religious group from pregnancy to 9 years post-partum for partners (*n* = 2,358).

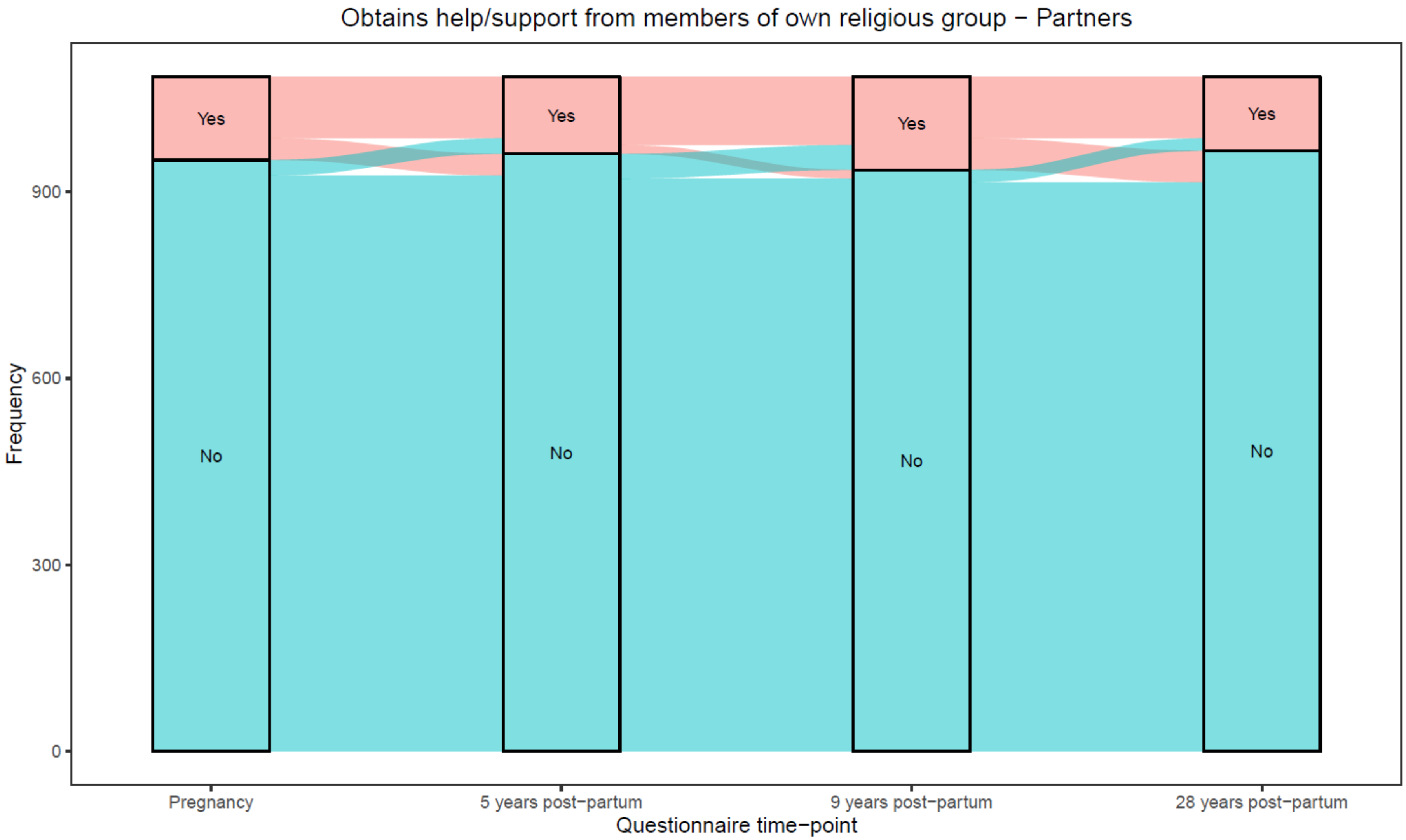

Figure S38: Change in whether obtain help/support from other members of own religious group from pregnancy to 28 years post-partum for partners (*n* = 1,085).

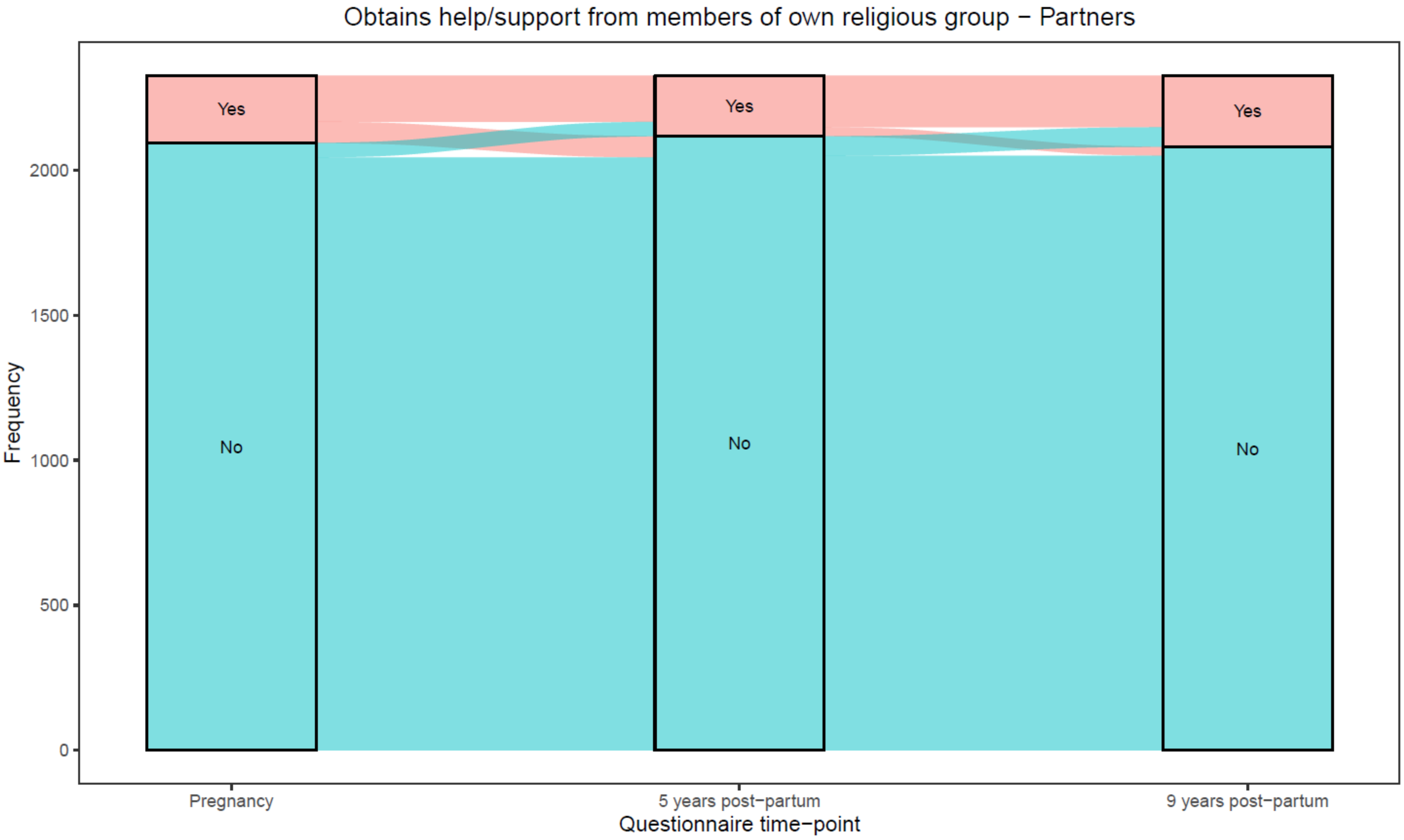

Figure S39: Change in whether obtain help/support from other members of own religious group from pregnancy to 9 years post-partum for partners (*n* = 2,325).

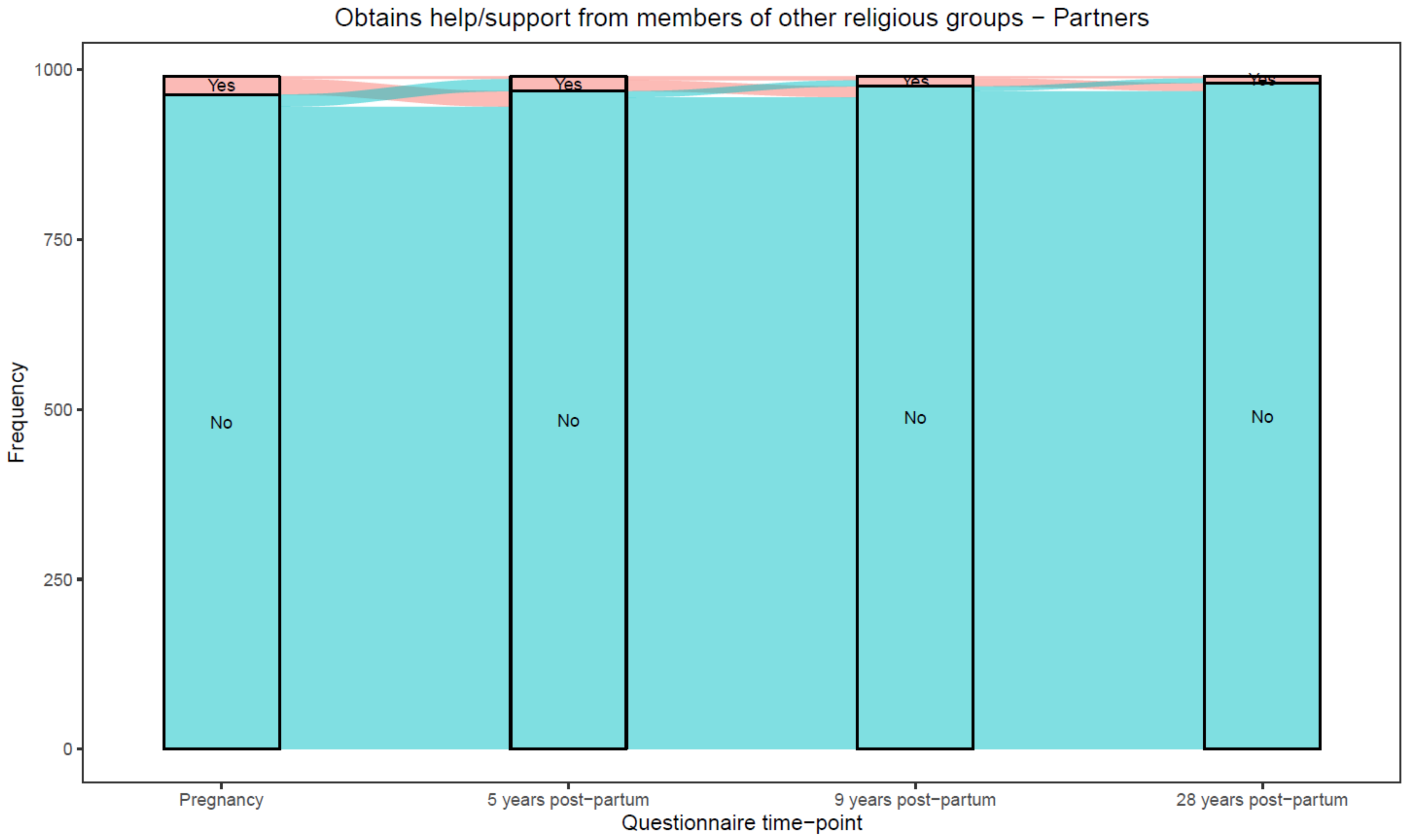

Figure S40: Change in whether obtain help/support from members of other religious groups from pregnancy to 28 years post-partum for partners (*n* = 990).

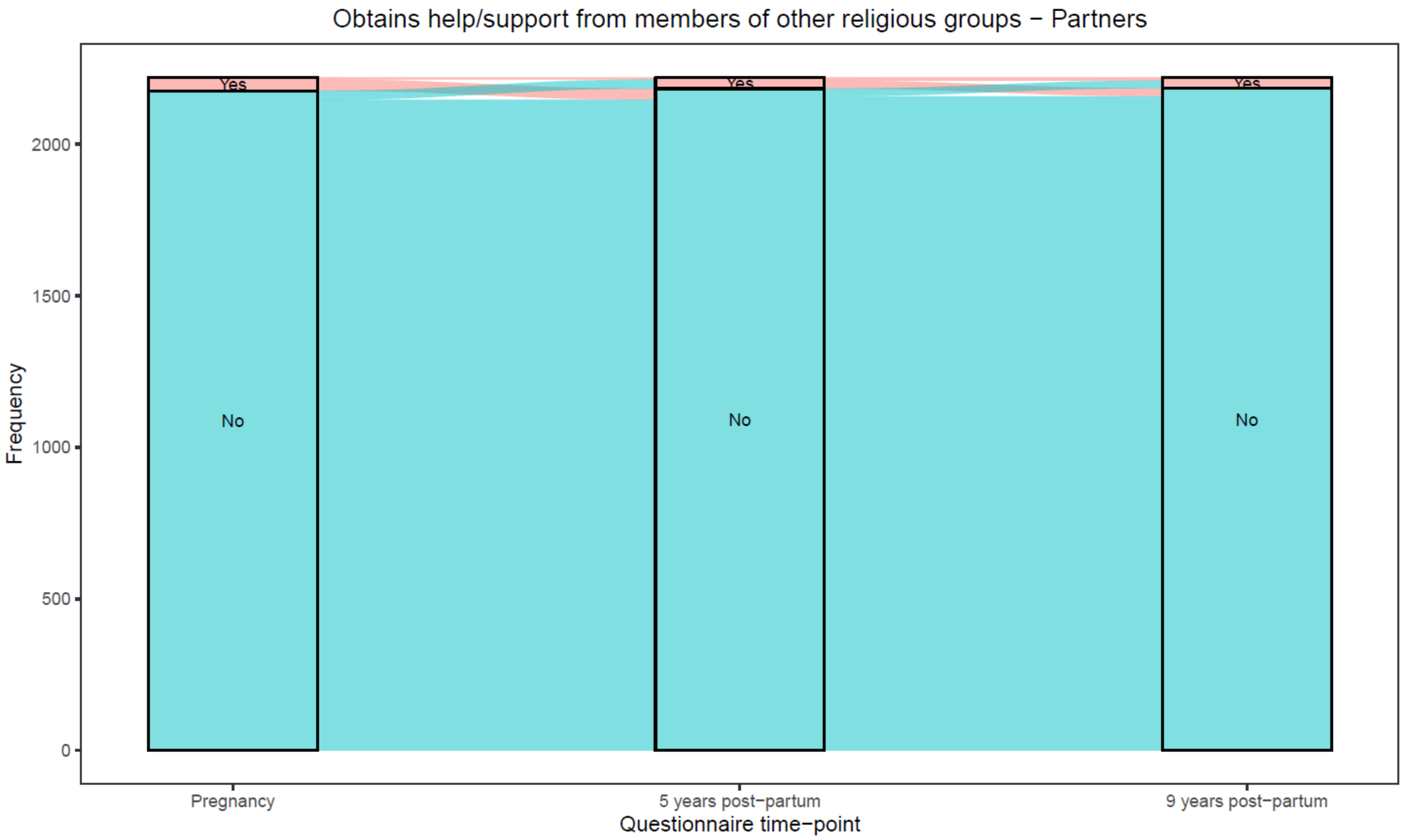

Figure S41: Change in whether obtain help/support from members of other religious groups from pregnancy to 9 years post-partum for partners (*n* = 2,220).

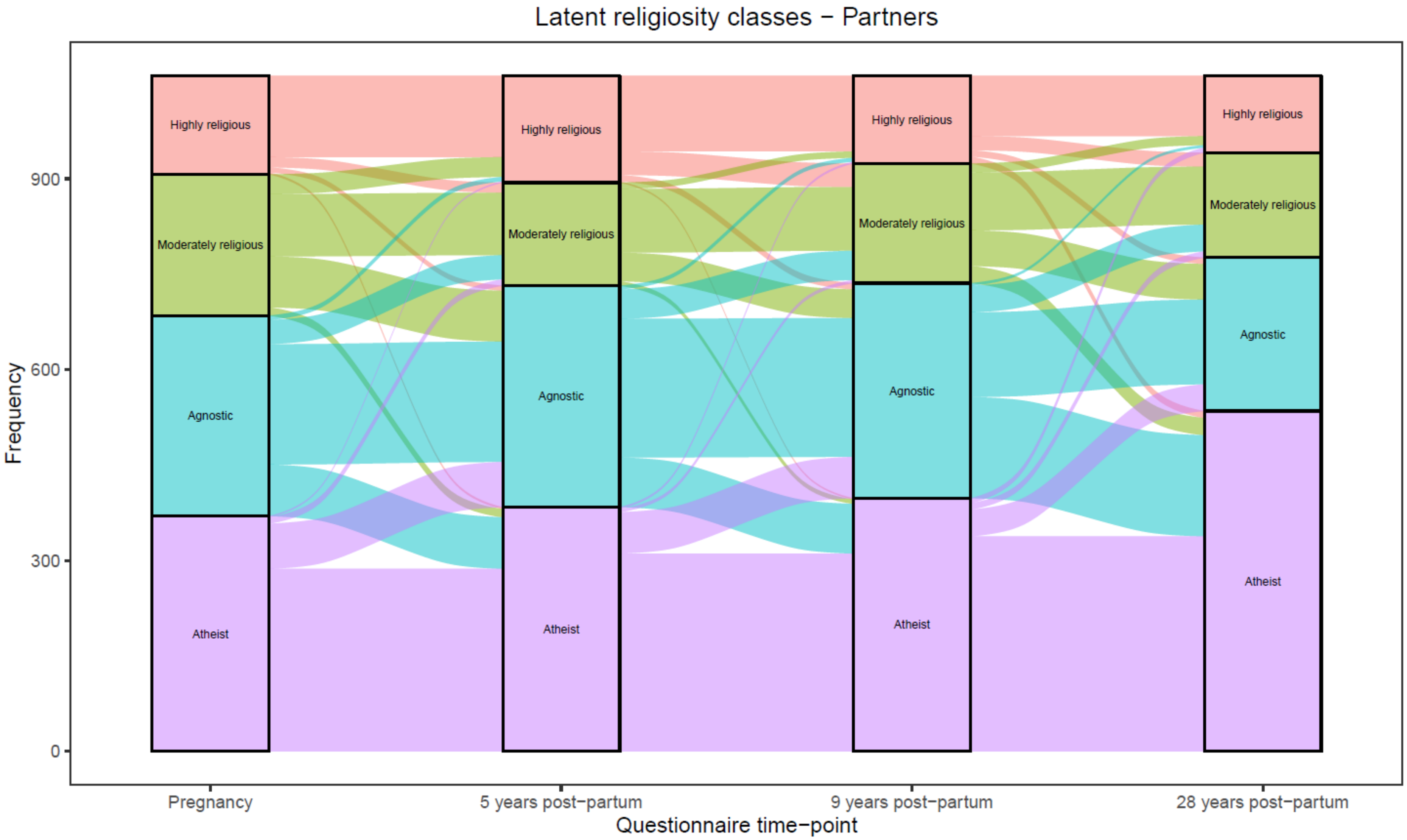

Figure S42: Change in the religiosity latent classes from pregnancy to 28 years post-partum for partners (*n* = 1,062).

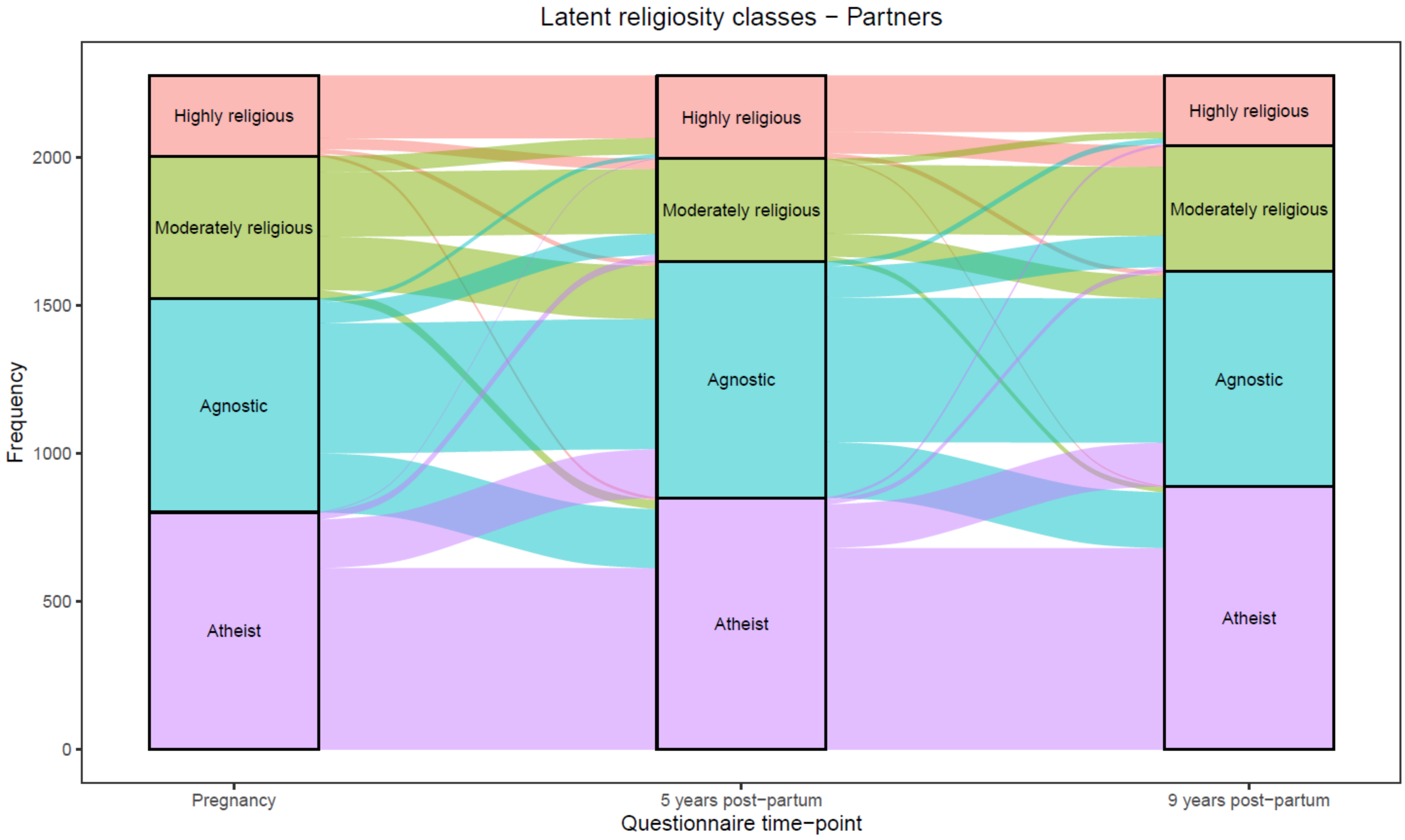

Figure S43: Change in the religiosity latent classes from pregnancy to 9 years post-partum for partners (*n* = 2,227).

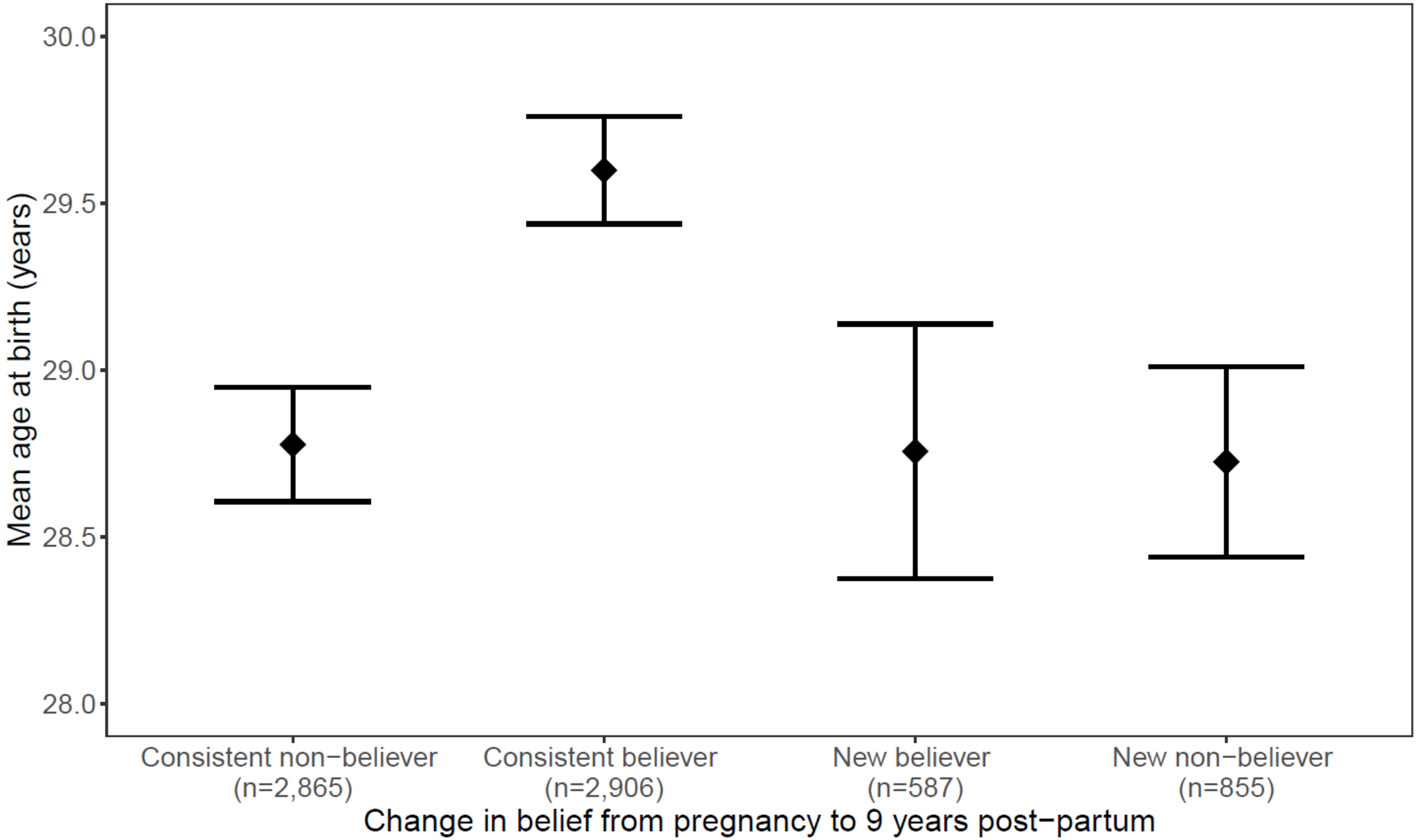

Figure S44: Differences in age at birth between the ‘belief in God/a divine power’ trajectories using method 1 in table 3 (*n* = 7,213). Diamonds denote the mean values, and error bars are 95% confidence intervals.

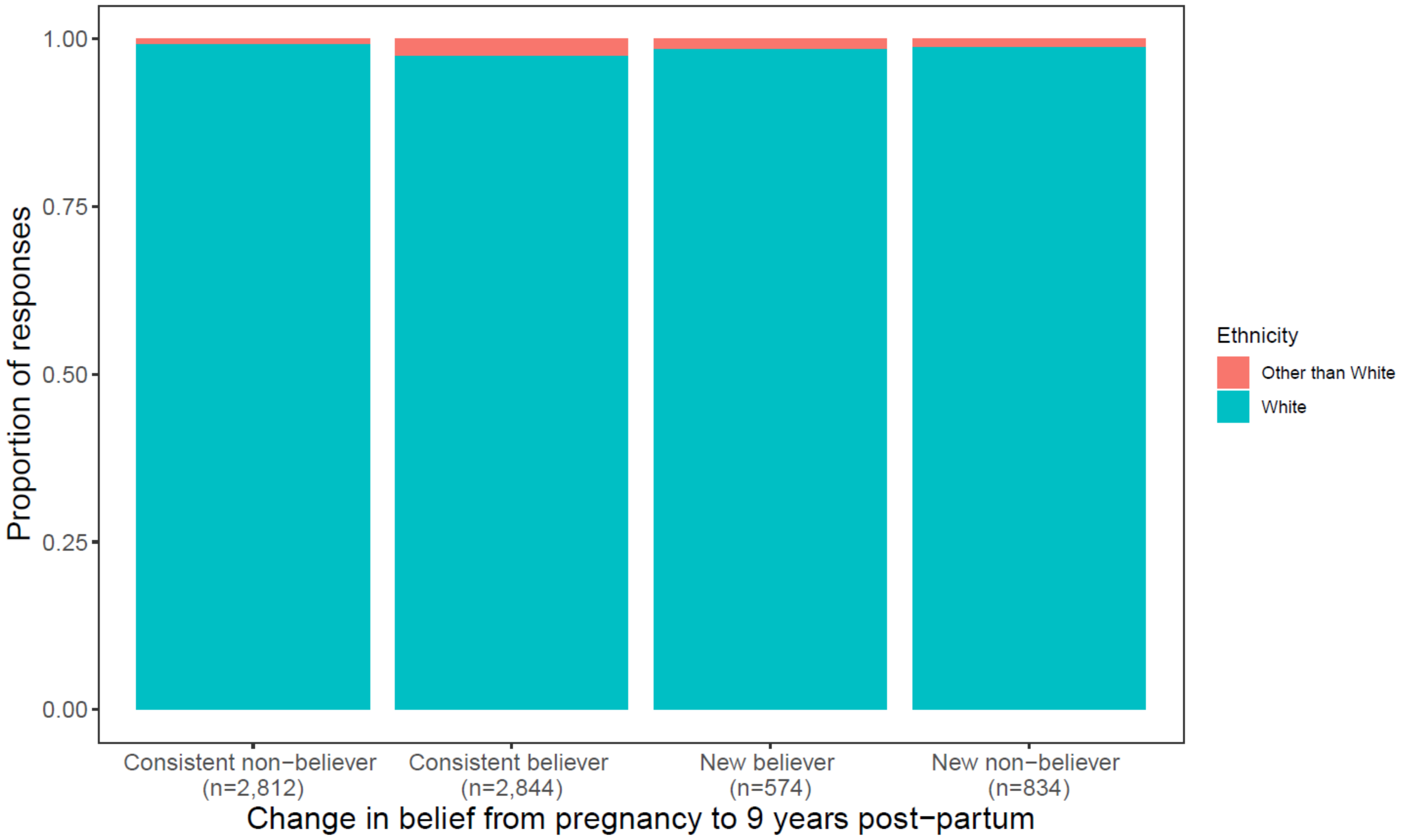

Figure S45: Stacked bar-plot displaying differences in ethnicity between the 'belief in God/a divine power' trajectories using method 1 in table 3 (*n* = 7,064).

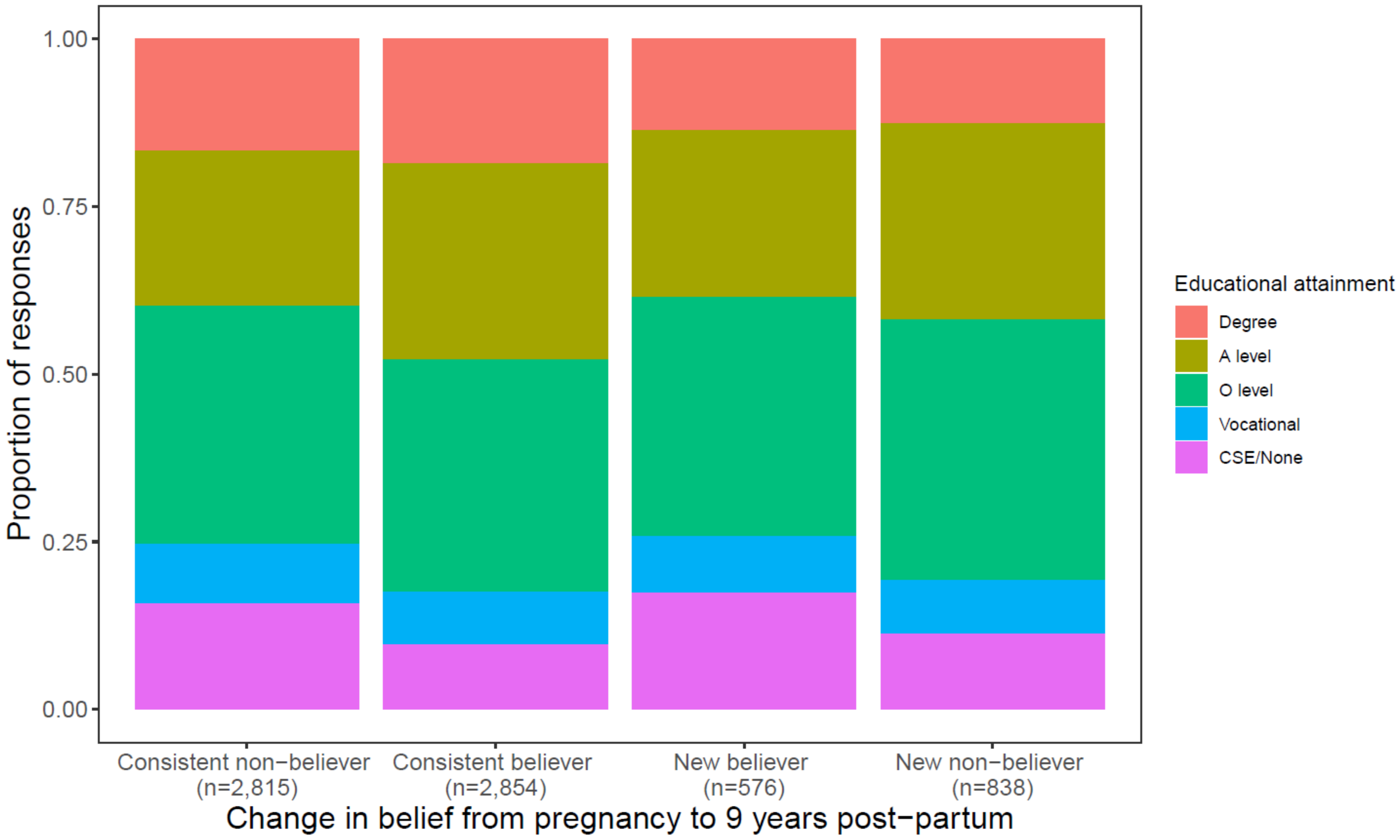

Figure S46: Stacked bar-plot displaying differences in educational attainment between the 'belief in God/a divine power' trajectories using method 1 in table 3 (*n* = 7,083).

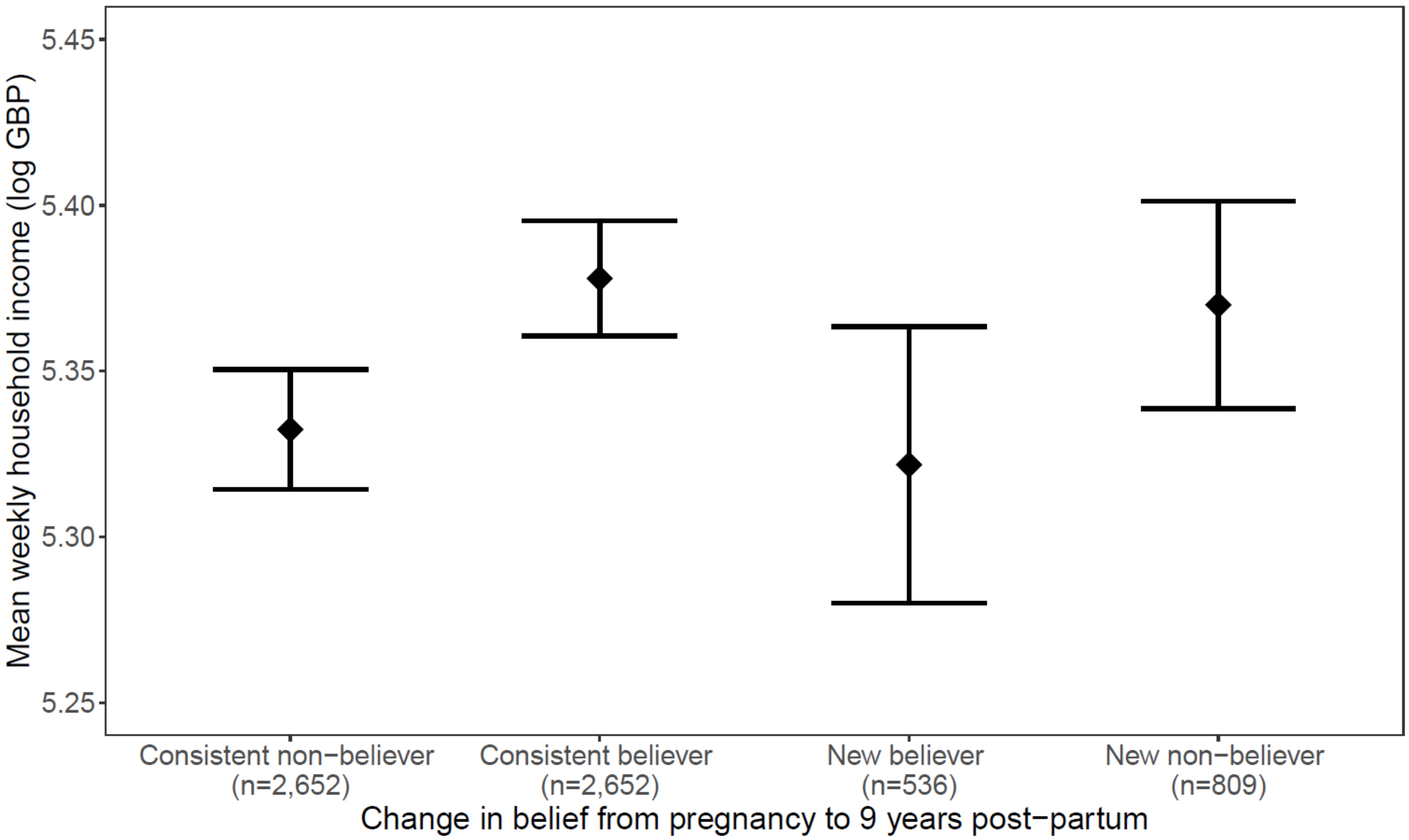

Figure S47: Differences in household income between the 'belief in God/a divine power' trajectories using method 1 in table 3 (*n* = 6,649). Diamonds denote the mean values, and error bars are 95% confidence intervals.

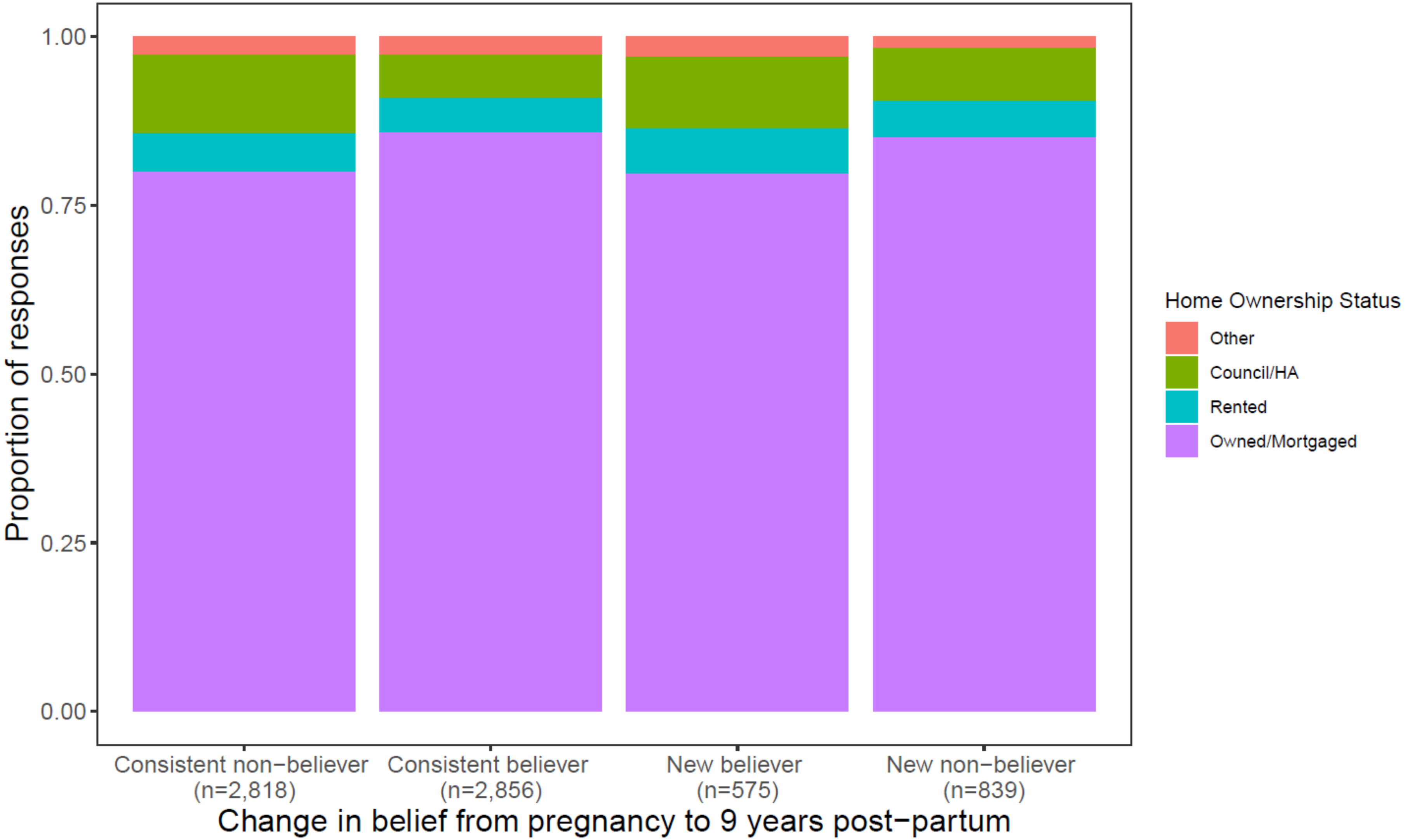

Figure S48: Stacked bar-plot displaying differences in home ownership status between the 'belief in God/a divine power' trajectories using method 1 in table 3 (*n* = 7,088).

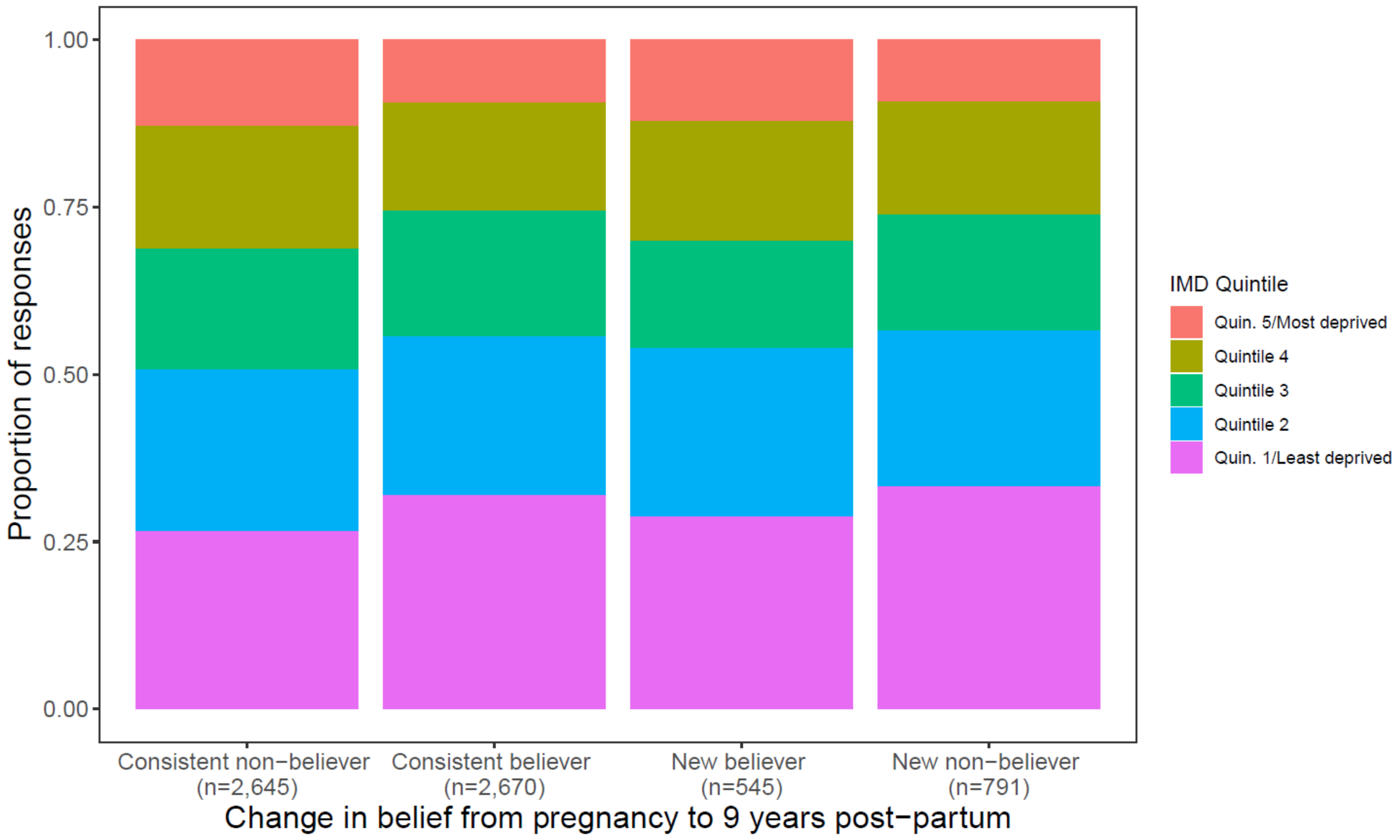

Figure S49: Stacked bar-plot displaying differences in area-level index of multiple deprivation (IMD) status between the 'belief in God/a divine power' trajectories using method 1 in table 3 (*n* = 6,651).

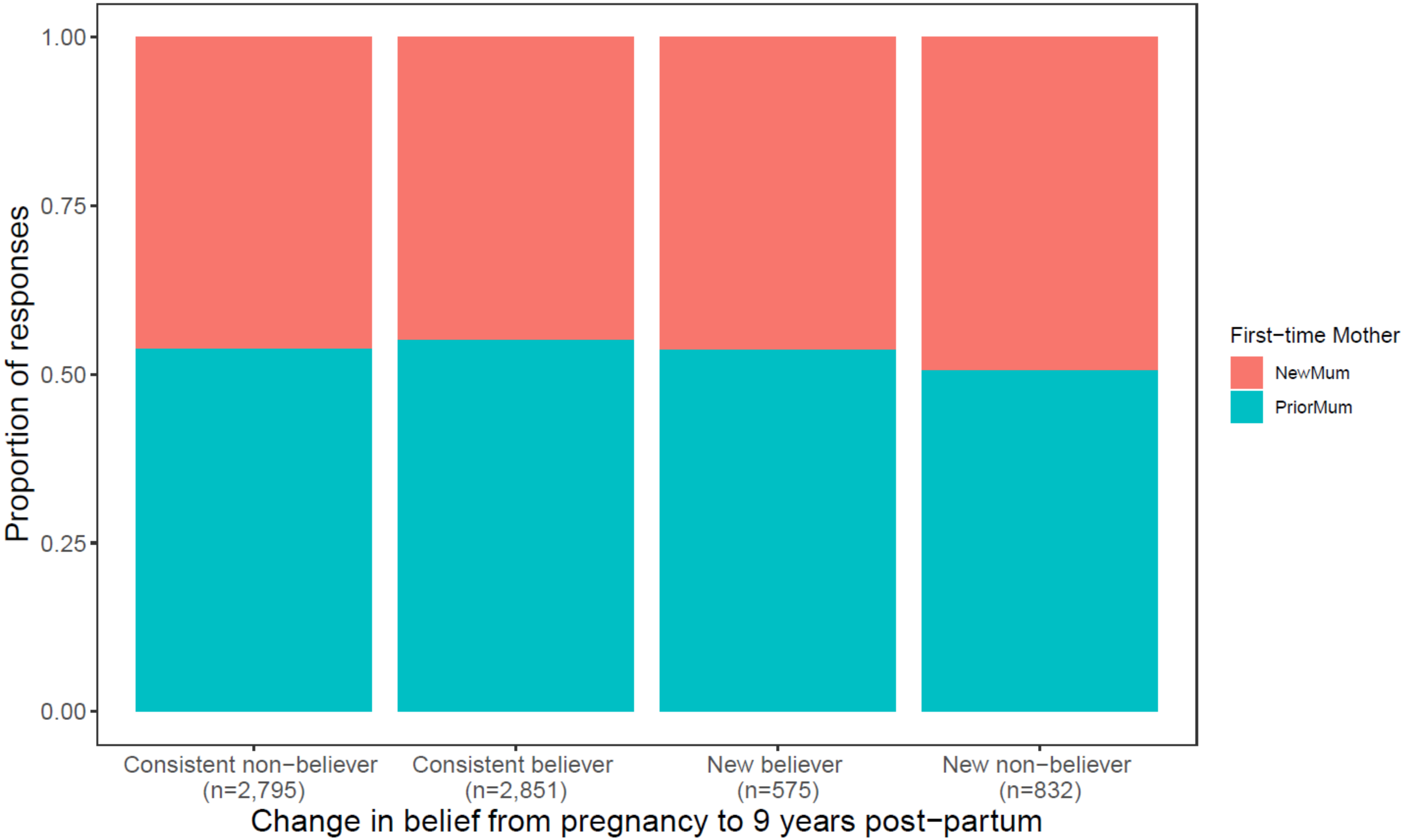

Figure S50: Stacked bar-plot displaying differences in first-time mother status between the ‘belief in God/a divine power’ trajectories using method 1 in table 3 (*n* = 7,053).

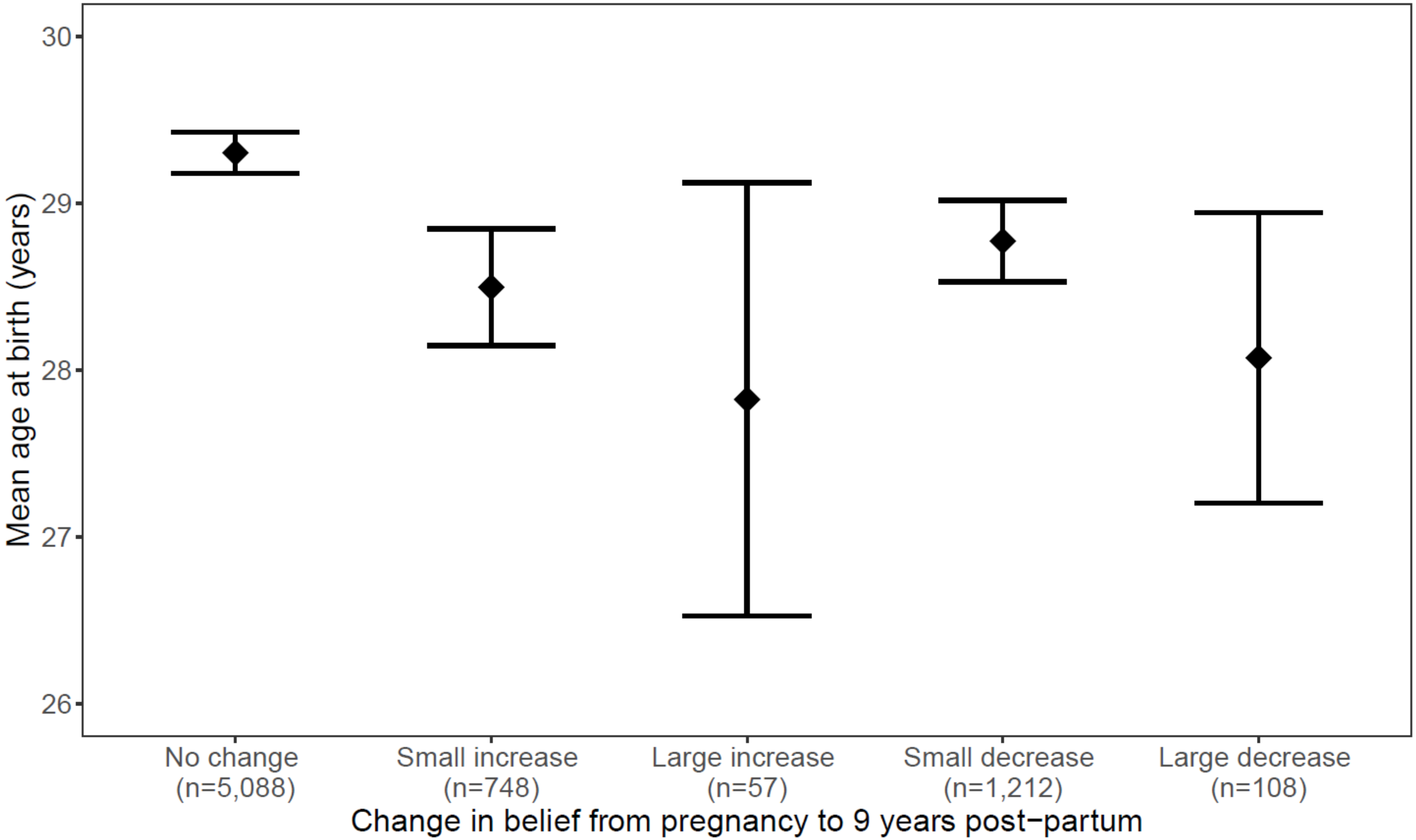

Figure S51: Differences in age at birth between the 'belief in God/a divine power' trajectories using method 2 in table 3 (*n* = 7,213). Diamonds denote the mean values, and error bars are 95% confidence intervals.

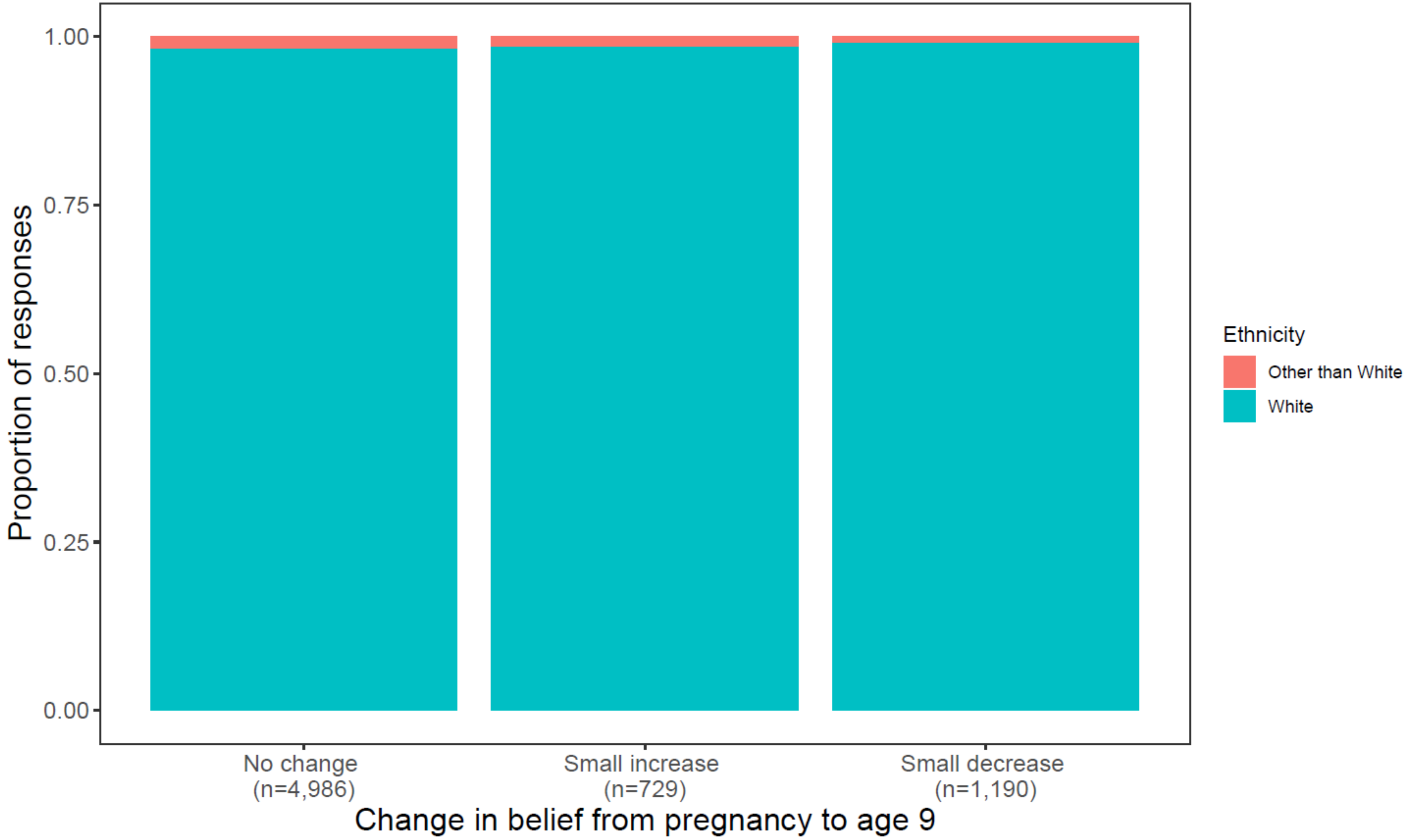

Figure S52: Stacked bar-plot displaying differences in ethnicity between the 'belief in God/a divine power' trajectories using method 2 in table 3 (*n* = 7,064). Note that the 'large increase' and 'large decrease' outcomes have been suppressed due to small and potentially disclosive cell counts (< 5).

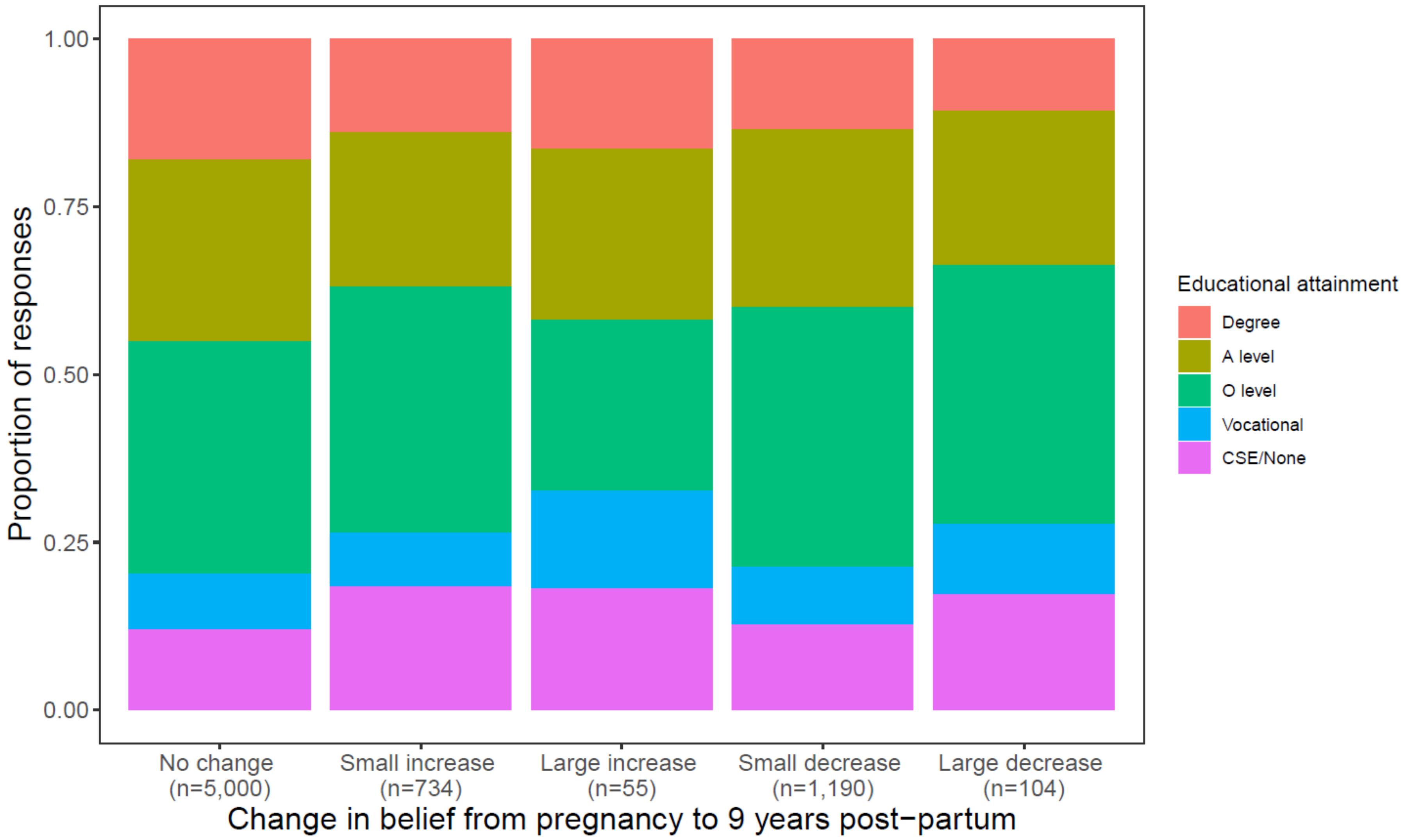

Figure S53: Stacked bar-plot displaying differences in educational attainment between the 'belief in God/a divine power' trajectories using method 2 in table 3 (*n* = 7,083).

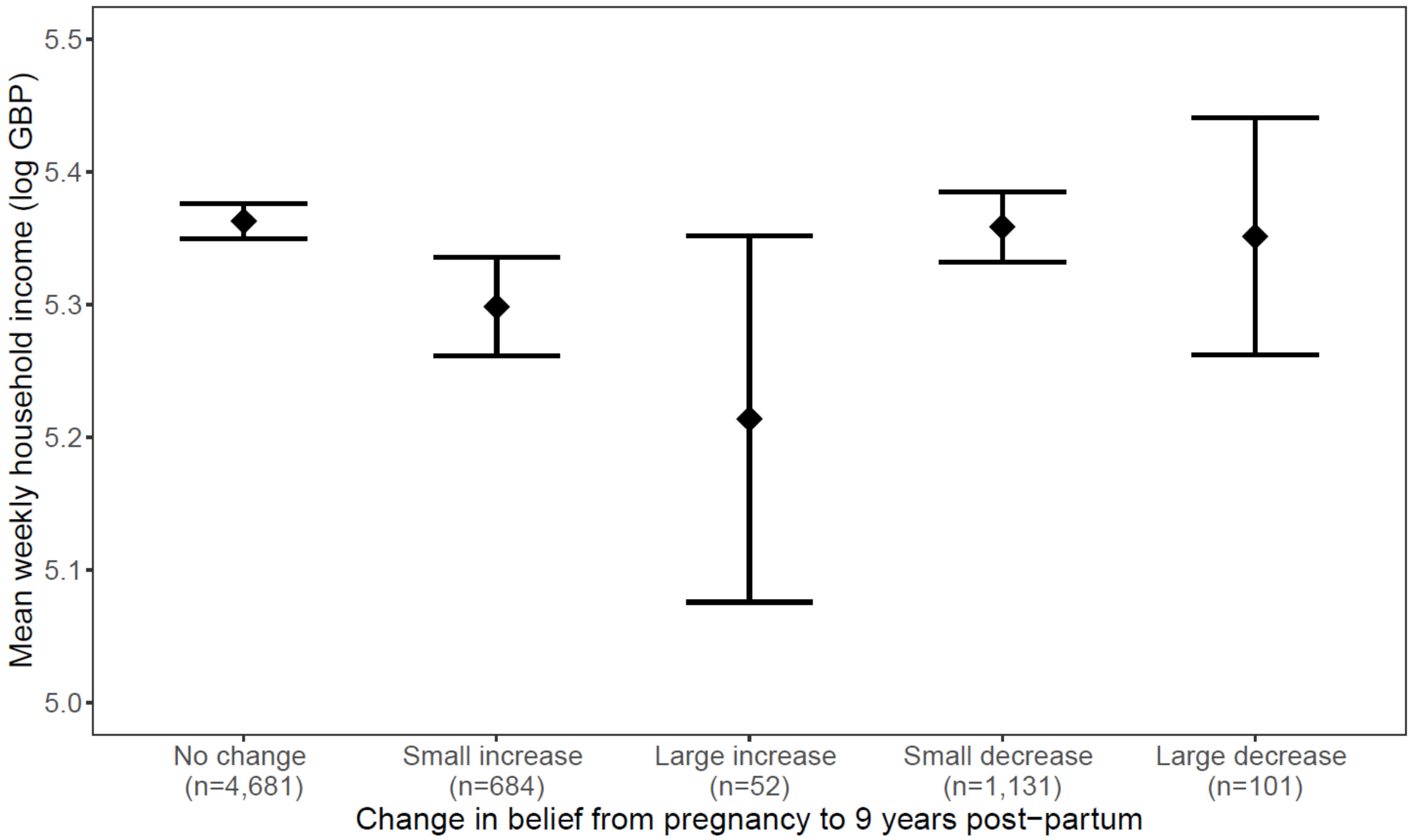

Figure S54: Differences in household income between the 'belief in God/a divine power' trajectories using method 2 in table 3 (*n* = 6,649). Diamonds denote the mean values, and error bars are 95% confidence intervals.

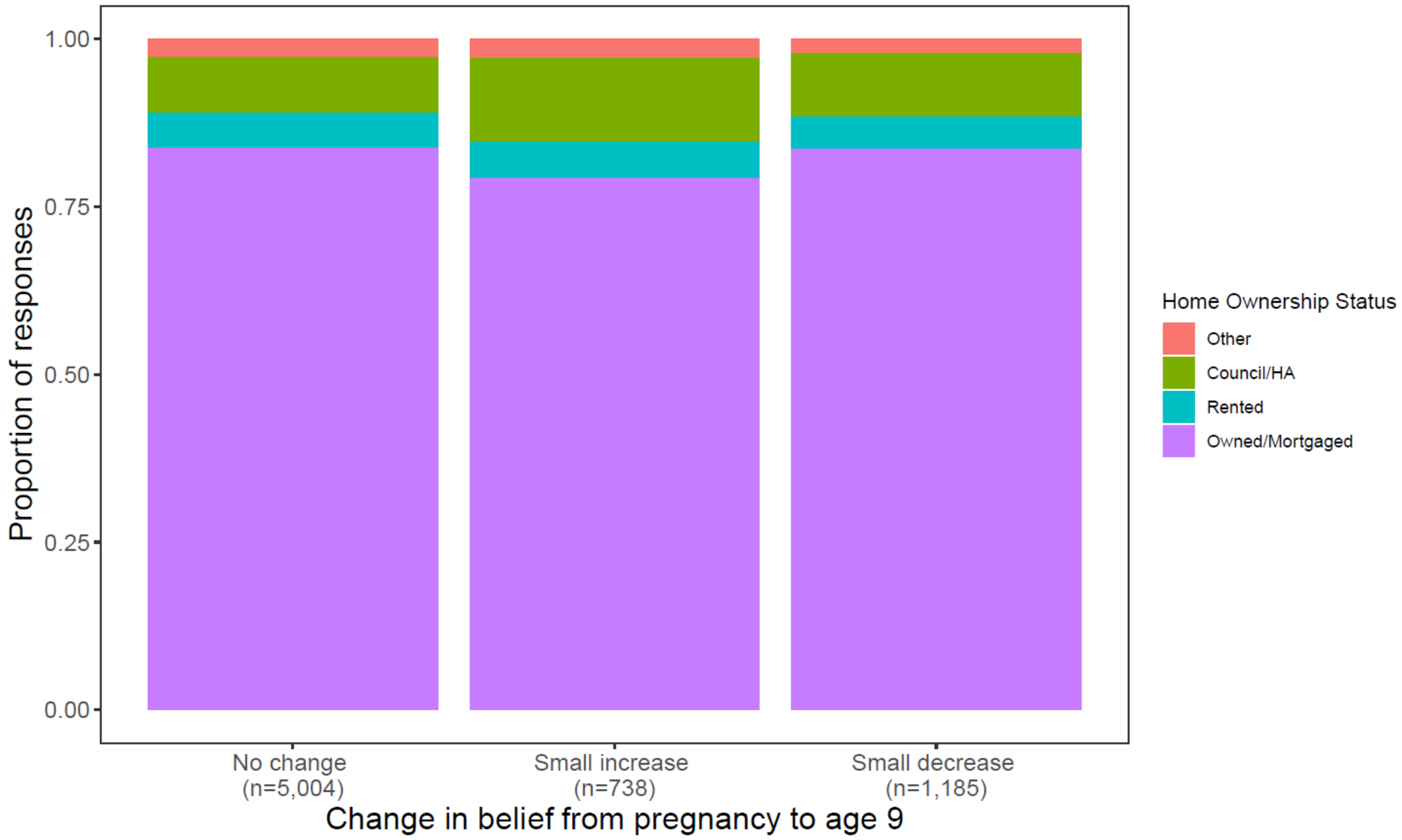

Figure S55: Stacked bar-plot displaying differences in home ownership status between the 'belief in God/a divine power' trajectories using method 2 in table 3 (*n* = 7,088). Note that the 'large increase' and 'large decrease' outcomes have been suppressed due to small and potentially disclosive cell counts (< 5).

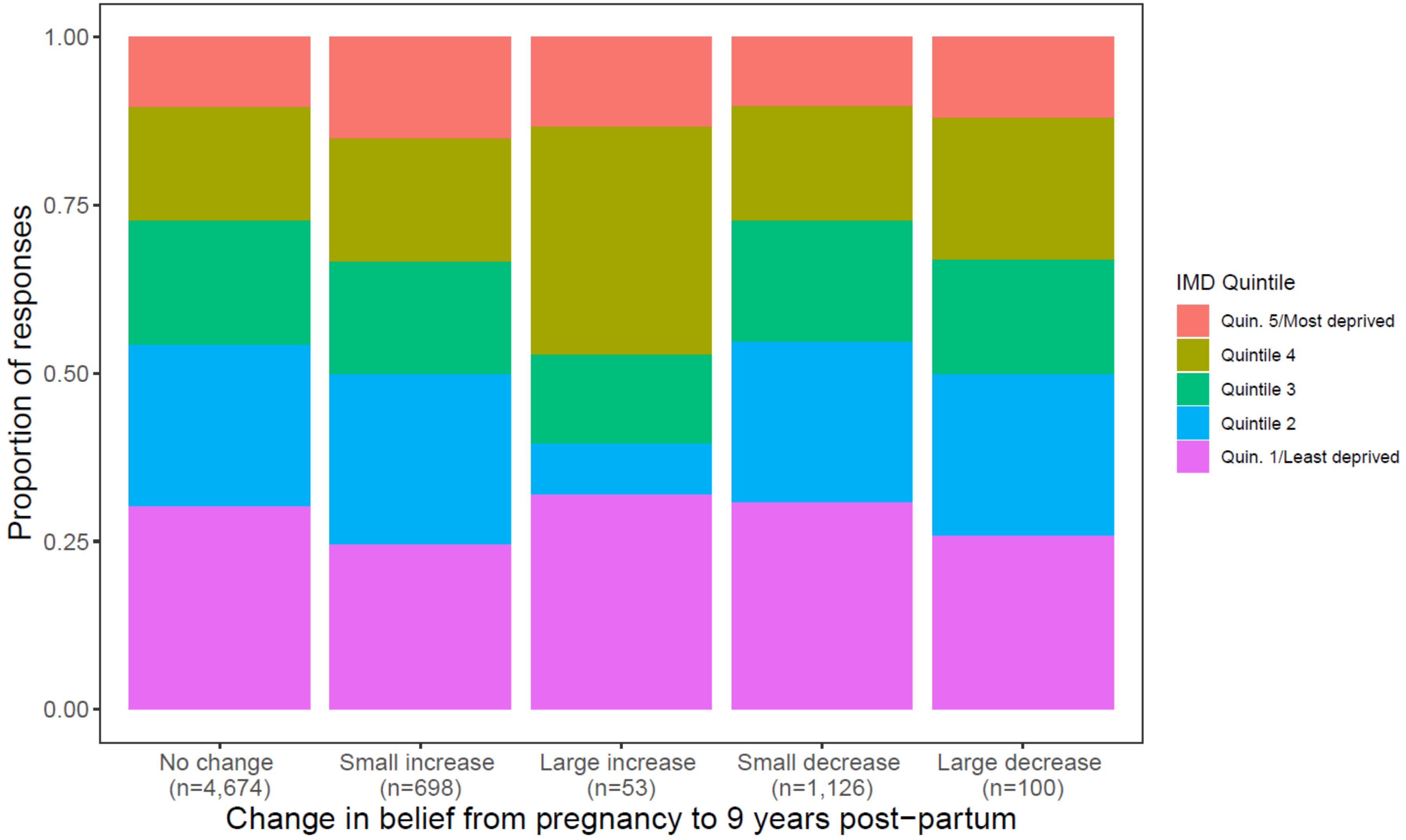

Figure S56: Stacked bar-plot displaying differences in area-level index of multiple deprivation (IMD) status between the 'belief in God/a divine power' trajectories using method 2 in table 3 (*n* = 6,651).

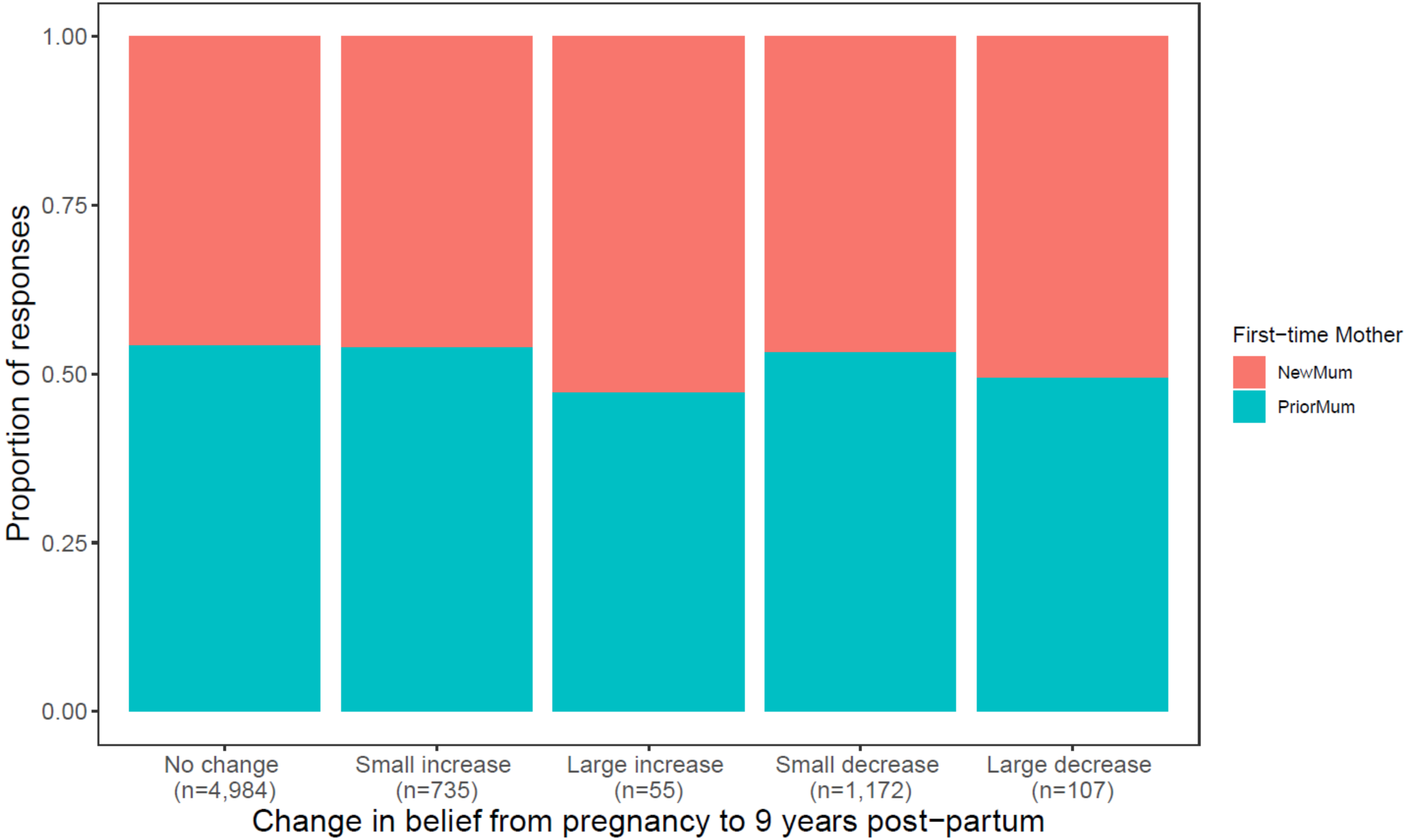

Figure S57: Stacked bar-plot displaying differences in first-time mother status between the 'belief in God/a divine power' trajectories using method 2 in table 3 (*n* = 7,053).
